# 9000 years of change in coral community structure and accretion in Belize reefs, western Atlantic

**DOI:** 10.1038/s41598-023-38118-5

**Published:** 2023-07-13

**Authors:** Eberhard Gischler, J. Harold Hudson, Anton Eisenhauer, Soran Parang, Michael Deveaux

**Affiliations:** 1 Institute of Geosciences, Goethe-University, 60438 Frankfurt am Main, Germany; 2 ReefTech Inc., Miami, FL 33143 USA; 3 GEOMAR Helmholtz Center of Ocean Research, 24148 Kiel, Germany; 4 Department of Earth and Environmental Sciences, University of Ottawa, Ottawa, ON K1N 6N5 Canada; 5 GSI Helmholtz Center of Heavy Ion Research, 64291 Darmstadt, Germany

**Keywords:** Palaeontology, Palaeoecology, Sedimentology

## Abstract

Tropical coral reefs, as prominent marine diversity hotspots, are in decline, and long-term studies help to improve understanding of the effects of global warming, sea-level rise, ocean acidification, deterioration of water quality, and disease. Here, we evaluated relative coral abundance and reef accretion rates over the past 9000 years in Belize barrier and atoll reefs, the largest reef system in the Atlantic Ocean. *Acropora palmata* and *Orbicella* spp. have been the most common corals. The abundance of competitive, fast-growing acroporids was constant over multi-millennial timescales. A decline in *A. cervicornis* abundance, however, and three centennial-scale gaps in *A. palmata* occurrence, suggest that the modern decline in acroporids was not unprecedented. Stress-tolerant corals predominate at the beginning of Holocene successions. Following the improvement of environmental conditions after inundation of the reef pedestal, their abundance has decreased. The abundance of weedy corals has increased during the Holocene underlining the importance of fecundity for the coral community. Reef-accretion rate, as calculated based on 76 new U-series age dates, has decreased over the Holocene and the mean value of 3.36 m kyr^−1^ is at the lower end of global reef growth compilations and predicted future rates of rise in sea level.

## Introduction

The status of tropical coral reefs has been declining due to warming, increase in cyclone strength and frequency, ocean acidification, pollution, and disease^1–6^. In the Caribbean realm, live coral cover has diminished by 80% in three decades^7^, many reefs are no longer dominated by corals but fleshy algae^8^, reef-building acroporid and massive faviid corals are reduced in abundance and have been replaced by weedy, generalistic taxa^9–11^, and reef accretion potential is low (mean of 1.87 ± 2.16 mm yr^−1^)^12^. These dramatic changes threaten ecological functionality balances, may lead to the collapse of habitat complexity with a concomitant loss in biodiversity, a decrease in calcium carbonate production and reef accretion, decline in framework resilience, and eventually compromise environmental service provisions^1–3, 5, 10, 12^. Millions of people are living along tropical coasts and on low-lying reef islands and are therefore dependent on coral reefs socio-economically. The alliance of the 39 small island states (AOSIS) alone, all of them situated adjacent to coral reefs and many of them located just a few meters above sea level, provide the homelands for some 80 million people, i.e., one percent of the world's population. Therefore, the issue of understanding and addressing coral reef decline is not purely scientific.

**Figure 1:**
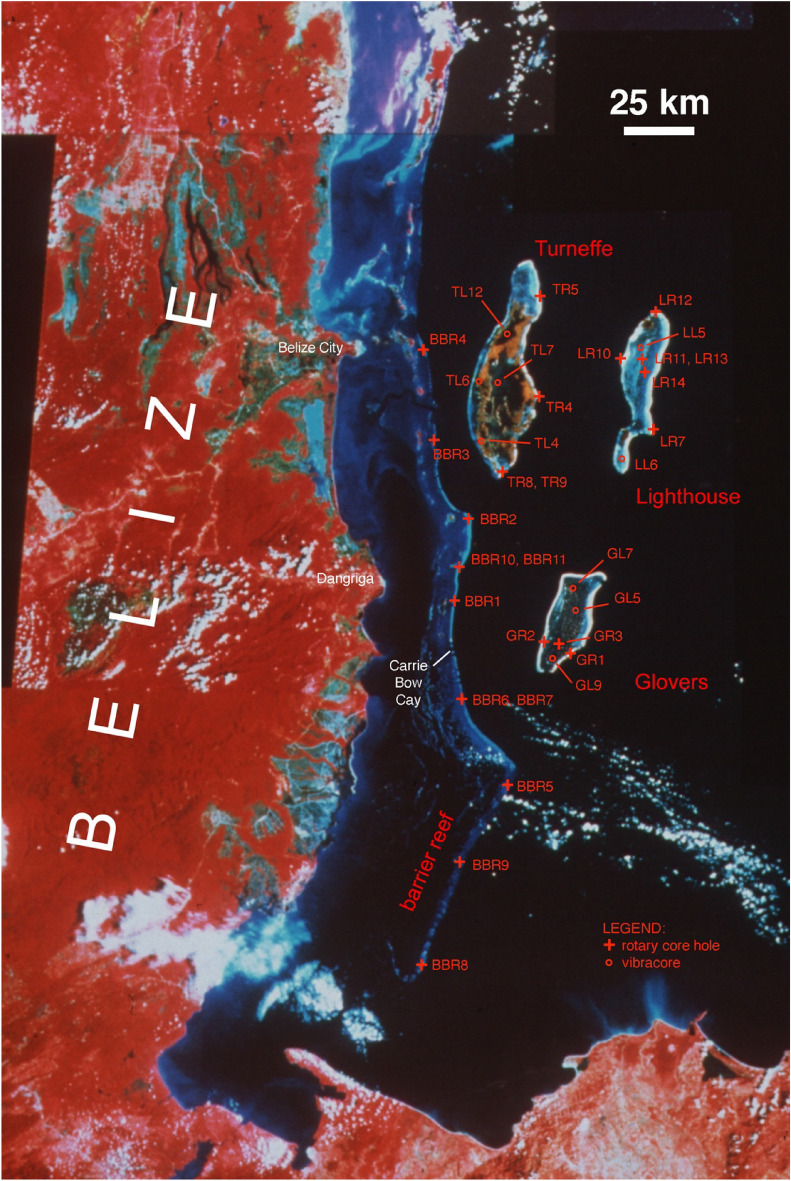
Locations of analyzed drill cores along Belize barrier and atolls reefs on Landsat MSS satellite image (false-color infrared composite; MSS bands 7, 5, 4 combined in red, green, blue colors)^13^. Crosses mark rotary core locations; open circles mark vibracore stations. The latter have been added to analysis to add available basal peat data as sea-level indicator. For latitudes and longitudes of core locations see Supplementary Table 5.

The study of drill core material extracted from modern coral reefs allows detailed and systematic reconstructions of environmental conditions during the Holocene based on sedimentological, paleoecological, and geochronological analyses. Based on these data, previous ecological and environmental changes can be reconstructed that may allow scientists and managers to decide whether or not current declines observed in corals and coral reefs are unprecedented and may serve as a baseline for future predictions. Therefore, we have revisited 22 drill cores extracted from the barrier and atoll reefs of Belize (Fig. 1), which represents the largest coral reef structure in the Atlantic Ocean, to quantify reef-accretion rates and coral community structure as indicator of environmental changes over the past several thousand years based on new and existing absolute age data.

## Study area

The Belize Barrier Reef is a 250 km long, relatively continuous and surface-breaking structure^13^ (Fig. 1). Several channels create narrow interruptions in the reef up to 10 m deep. In the central barrier reef area, there is an up to 6 km wide barrier reef platform, where water depths do not exceed 5 m. To the north and south, the barrier reef platform narrows considerably to less than 2 km. There, surface-breaking coral reefs at the barrier reef proper are less continuous and less abundant than in the central part. Patch reefs and lagoonal atolls are abundant on the southern shelf. Whereas the northern shelf north of Belize City is not deeper than 6 m, the southern shelf deepens to some 50 m at its southern end. The offshore atolls are characterized by well-developed reef margins and interior lagoons the maximum depths of which range from 8 to 18 m. Barrier-reef and atoll margins exhibit zonation from inshore to offshore including a sand apron, a cemented pavement, the reef crest, and the shallow fore-reef slope^14–18^. The width of the fore-reef slope varies considerably from less than 100 m to more than 1 km. Between 10 and 40 m water depth, a drop-off is developed that is located at the top of an almost vertical wall. The base of the wall, at about 100 m depth, is onlapped by a sediment talus slope^19^.

The climate of the area is tropical with average air temperatures from 25 °C in the winter to 29 °C in the summer, as measured on the central barrier reef at the Smithsonian field station on Carrie Bow Cay^20^. Trade winds blow from the east for most of the year. In the winter months, winds generally blow from the north and northwest. Rainfall in Belize is highest from May to December, and increases from 150 cm yr^−1^ in the flat northern part of the country to 400 cm yr^−1^ in the mountainous south. Intra-annual variability of sea-surface temperatures (SST) at the Belize Barrier Reef ranged from 25 (winter) to 32 °C (summer) during 2000–2002 (Smithsonian field station). The offshore Belize area is microtidal. Tropical cyclones have passed the Belize reef system repeatedly during the Common Era and caused considerable damage in the reefs^6, 21^.

Shallow coring on the marginal barrier and atoll reefs during previous studies recovered > 20 m of Holocene reef accretion, which started around 9 kyrs BP^22–25^. Those studies indicated that reef accretion rates average ca. 3 m kyr^−1^ and show a decreasing trend during the Holocene^26^. Several environmental factors have been influencing Holocene reef growth in Belize including sea-level rise, variation in siliciclastic input from the mainland, exposure to waves and currents, and precipitation, as well as differential subsidence^25^.

## Results

### Reef accretion

**Figure 2:**
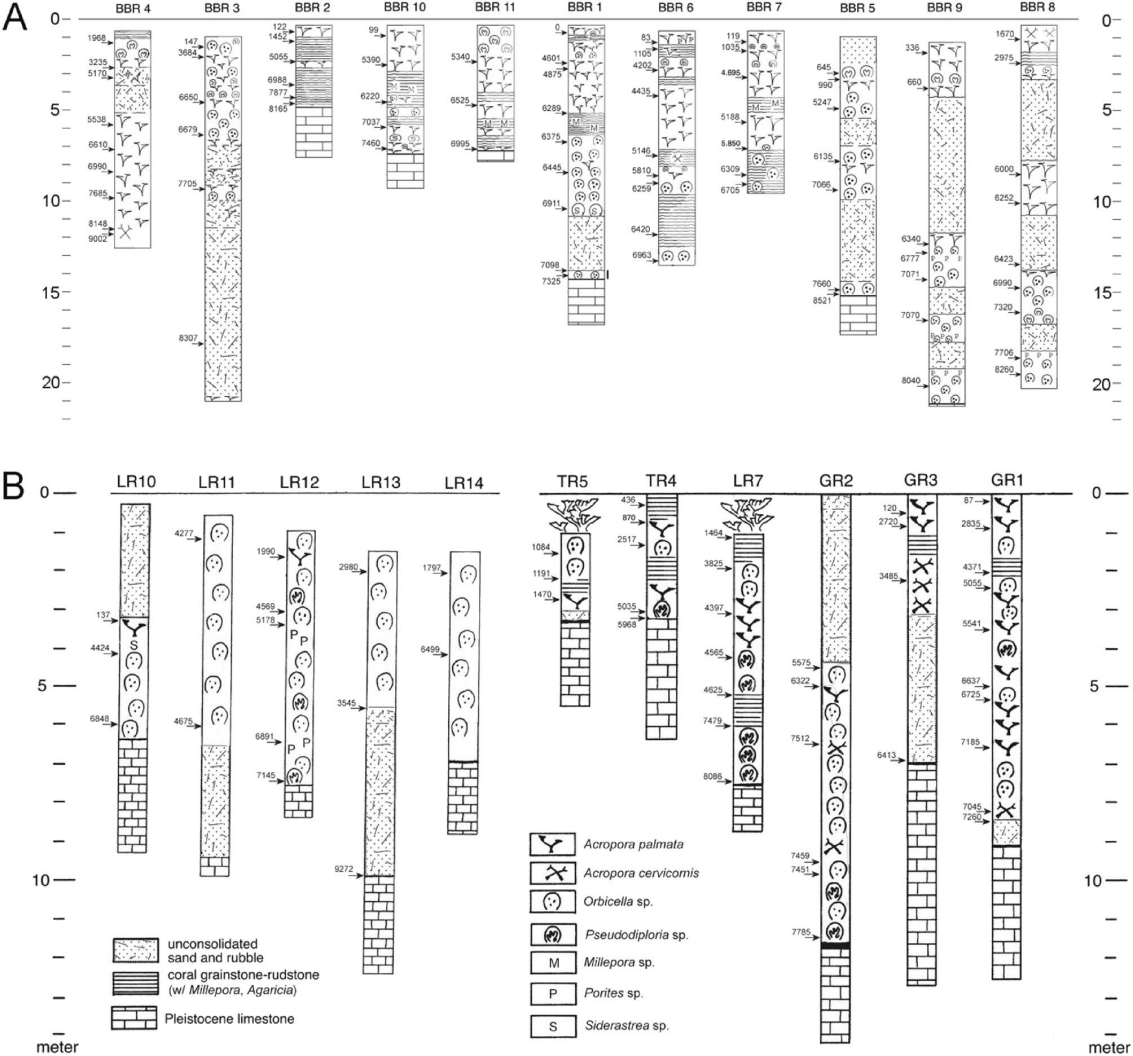
Simplified core logs^27^ including absolute age data from (**A**) the Belize barrier reef and (**B**) the Belize atolls. For detailed core logs also see Supplementary Fig. 4.

**Figure 3:**
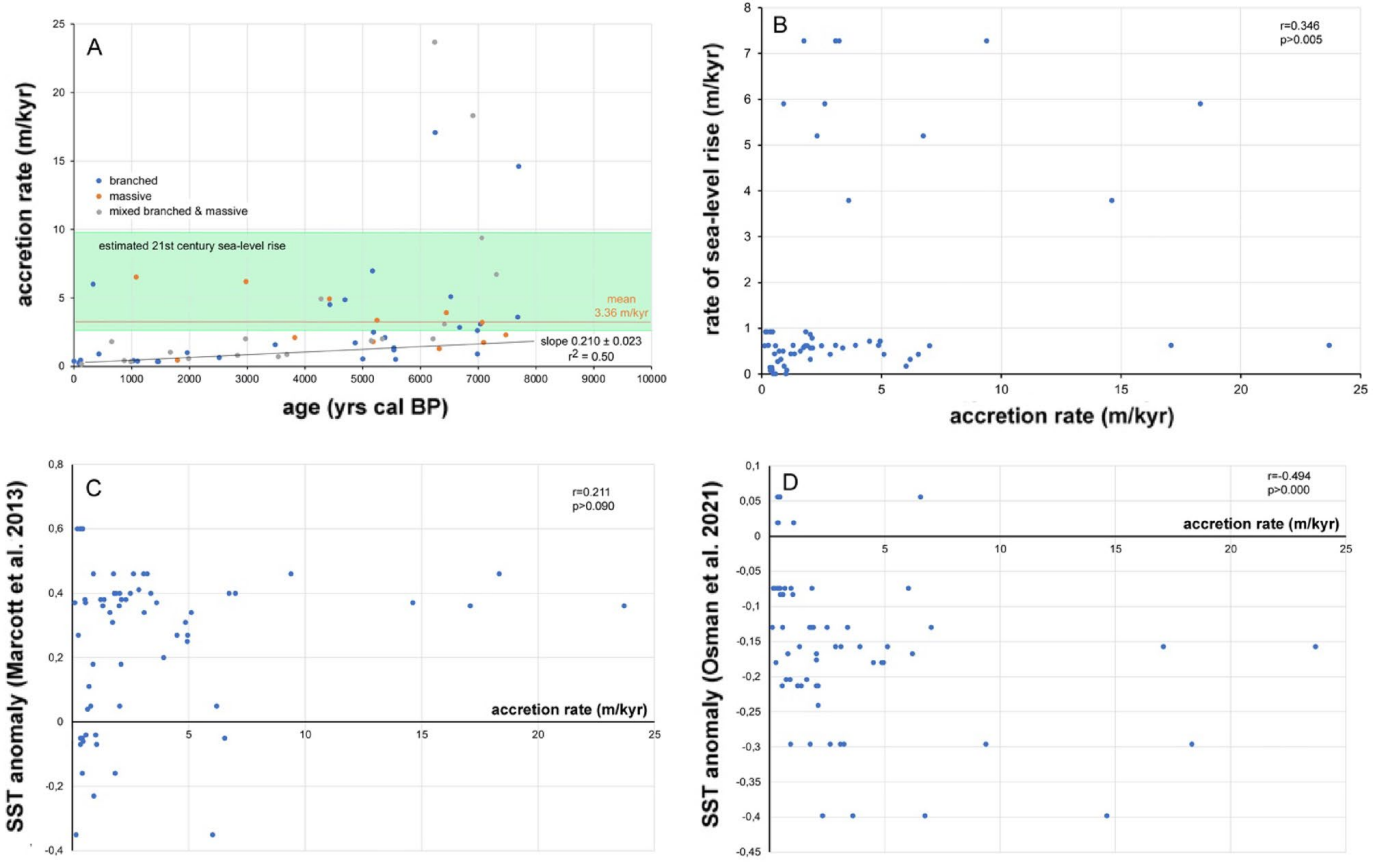
(**A**) Cross-plot of accretion rate versus age, including regression line and uncertainty of slope. Absolute rates of twenty-first century sea-level rise^29^ (0.26–0.98 m per century) have been extrapolated into m kyr^−1^ (green area). Note that reef sections dominated by branched and those by massive corals accrete at comparable rates. ‘Mixed’ includes reef sections where branched and massive corals occur at more or less the same frequency. (**B**) Reef accretion rate versus rate of sea-level rise. Rates of Holocene sea-level rise have been estimated based on ANU_HR_71p230 sea-level data at 6 sites (Supplementary Table 2; Supplementary Figs. 2, 3). Here, mean rates of sea-level rise in sites 1–6 have been used. (**C**, **D**) Accretion rate versus climate anomalies^30, 31^. Reef accretion rates in **A**–**D** have been calculated taking into account error ranges and error propagation^26^.

A total of 76 new U-series ages from corals were produced that range from 9 to 0 ka (Fig. 2; Supplementary Table 1), in addition to the existing 51 age data from the Belize reef drill cores. The absolute age data (n = 127) plotted along the core logs are shown on Fig. 2. Age-depth plots suggest a rapid rise of sea level until 6 ka and a subsequent, slow, asymptotic rise to present level (Supplementary Fig. 1). Sea-level data broadly match model curves, with the ANU_HR_71p230^28^ ice and earth model exhibiting the best overall fit (Supplementary Figs. 2, 3). Reef accretion rates average 3.36 m kyr^−1^ (SD = 4.56) and decrease during the Holocene (r^2^ = 0.50; regression line slope 0.210 ± 0.023 m kyr^−1^) (Fig. 3A; Supplementary Tables 2, 3). Branched (mean = 2.88 m kyr^−1^; SD = 3.94) and massive (mean = 3.16 m/kyr, SD = 1.93) coral reef accretion is in the same range and cannot be distinguished statistically based on a Kruskal–Wallis-test (H = 2.818, *p* > 0.096). Accretion rate correlates with the mean rate of sea-level rise (r = 0.441, *p* > 0.000) based on output from the optimal ANU model (Fig. 3B). The correlation of accretion rate with the Holocene temperature anomaly of Marcott et al.^30^ is insignificant (r = 0.207, *p* > 0.107), but significant in case of the temperature anomaly of Osman et al.^31^ (r = − 0.322, *p* > 0.011) (Fig. 3C,D).

### Coral community structure

**Figure 4:**
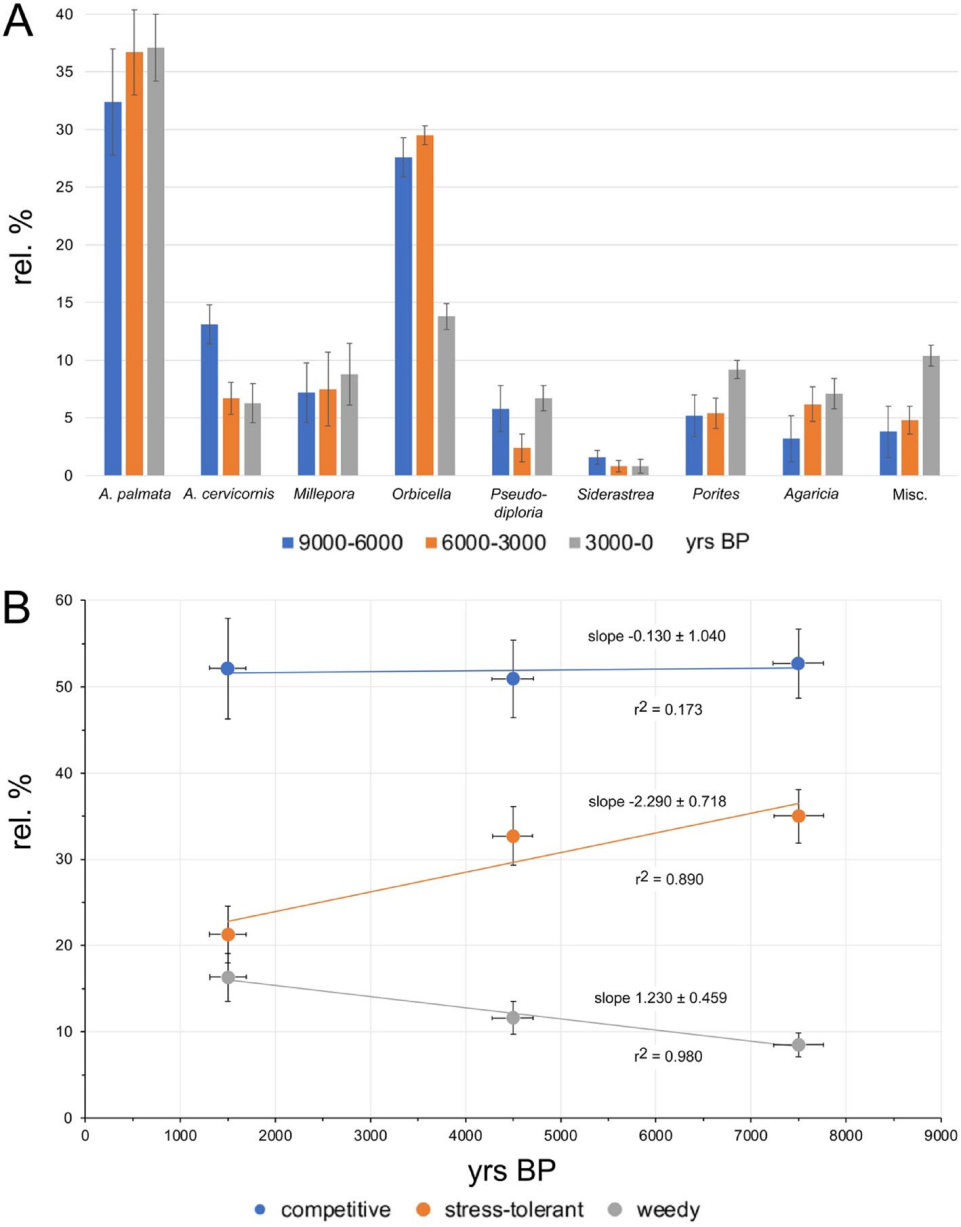
Age versus relative abundance plots of coral taxa (**A**) and of live-history traits^32^ of coral (**B**) in drill cores from Belize barrier and atoll reefs. 'Miscellaneous' includes other corals. The vertical error bars in (**A**) and (**B**) are the square root of N (and considering the number of corals in each time bin) as measure of uncertainty based on a Poisson distribution (reported as 1 standard error). The horizontal bars encompass the maximum error in age data in a time bin.

**Figure 5:**
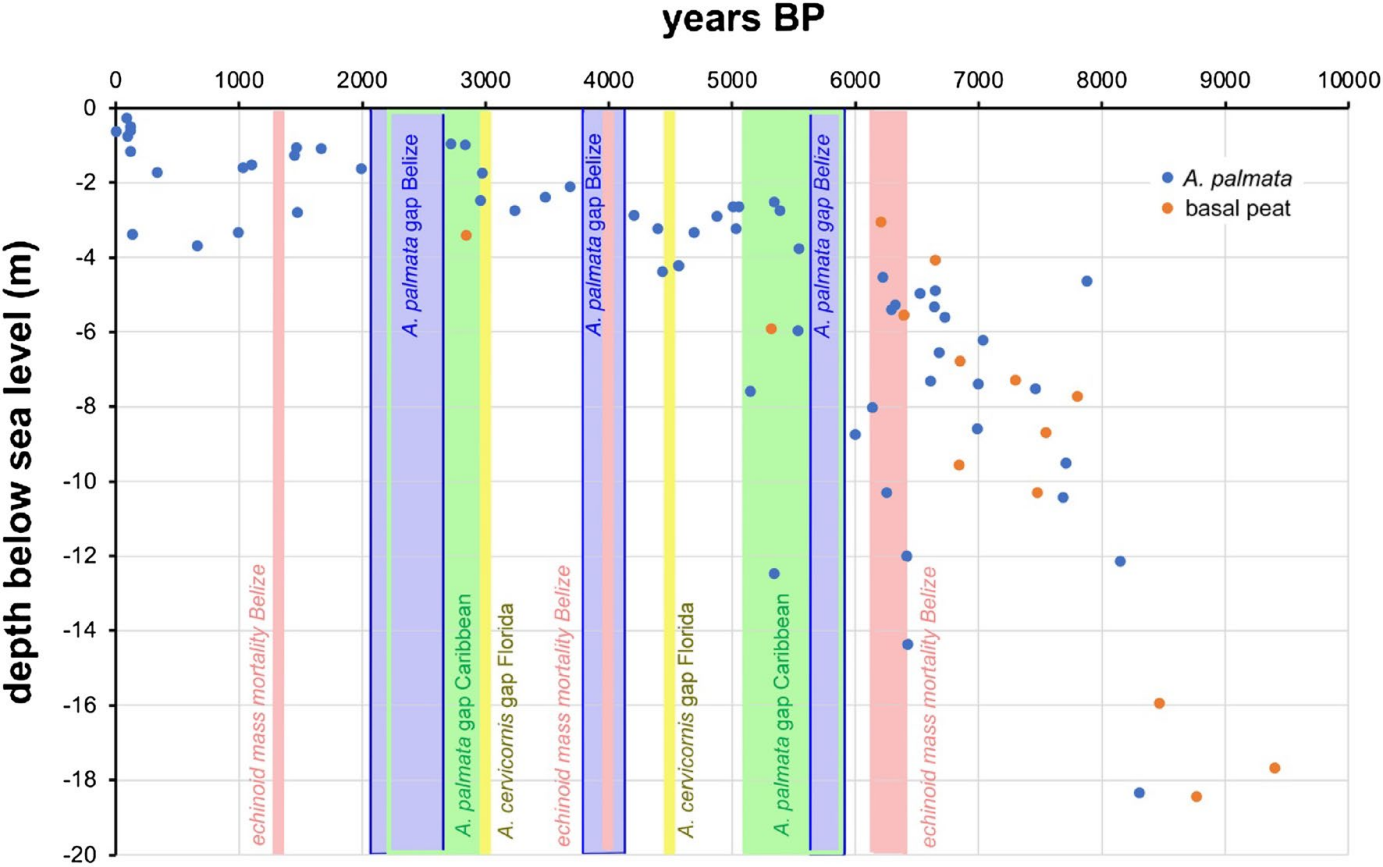
*Acropora palmata* occurrence gaps (blue) observed in the drill cores from Belize barrier and atoll reefs. Age data bounding the three gaps are 1.990 ± 18 to 2.720 ± 170, 3.684 ± 26 to 4.202 ± 30, and 5.541 ± 41 to 6.000 ± 170 ka, respectively (2-sigma probability). Data have been corrected for minimal subsidence. Only sea-level data from *A. palmata* and basal peat shown, which are indicative of shallow (< 5 m) water. Data on Holocene acroporid gaps are from Florida (yellow) and the wider Caribbean (green)^33, 34^. Data on Holocene echinoid mass mortality from Belize atolls (rose)^35^.

The most common Holocene corals include *Acropora palmata* (34.9 ± 2.1% relative abundance ± 1 standard error) and corals from the *Orbicella* group (25.2 ± 1.7%). They are followed in decreasing abundance by *A. cervicornis* (9.5 ± 1.0%), as well as species of *Millepora*, *Porites*, *Agaricia*, *Pseudodiploria*, and *Siderastrea*, with abundances of < 8% (Table 1; Supplementary Table 4). Other corals complete the assemblage with 5.6 ± 0.7% and include species of *Colpophyllia*, *Eusmilia*, *Favia*, *Meandrina*, and *Mussa*, with individual abundances of < 1%. In terms of life-history traits^32^, there are 52.0 ± 2.7% competitive, 31.3 ± 1.9% stress-tolerant, and 11.2 ± 1.1% weedy taxa. When subdividing the data into three time bins, based on the available age data, certain trends become visible (Table 1; Fig. 4). *Acropora palmata* abundance stays relatively constant through time, however, there are three apparent gaps in the Holocene record from 2.0 to 2.7, 3.7 to 4.2, and 5.5 to 6.0 ka (Fig. 5). Age data bounding these gaps are 1.990 ± 18 to 2.720 ± 170, 3.684 ± 26 to 4.202 ± 30, and 5.541 ± 41 to 6.000 ± 170 ka, respectively. *Acropora cervicornis* is most common in the older Holocene sections in that relative abundance in the 9–6 ka time bin is twice as high (13.1 ± 1.7%) as compared to the younger 6–3 and 3–0 ka time bins (6.7 ± 1.4% and 6.3 ± 1.7%, respectively) (Fig. 4A). The abundances of *A. palmata* and of hydrocoral *Millepora* species stay constant over time. At the base of our cores, directly overlying Pleistocene reef limestone, *Pseudodiploria* (n = 6) and *Orbicella* (n = 6) are most common (Supplementary Fig. 1), i.e., members of the stress-tolerant taxa are clearly dominating over acroporids (n = 2). A chi-square test showed that the abundances of coral live-history trait groups are not equally distributed (χ^2^ sum: 32.64; df: 6; *p* > 0.005). The abundance of the competitive group of corals exhibits no statistically significant trend through time (r^2^ = 0.173; regression line slope − 0.130 ± 1.040% kyr^−1^). The abundance of stress-tolerant taxa is generally declining throughout the Holocene (r^2^ = 0.890; regression line slope -2.29 ± 0.718%·kyr^−1^). In this group, *Orbicella* shows a significant decline in abundance from 29.5 ± 3.2% in the 6–3 ka to 13.8 ± 2.6% in the 3–0 ka time bin. *Pseudodiploria* abundance is decreasing at first from 5.8 ± 1.1% to 2.4 ± 0.8% and then increasing to 6.7 ± 1.7% within the three time bins. Conversely, the abundance of weedy taxa such as *Agaricia* and *Porites* is increasing over time (r^2^ = 0.980; regression line slope + 1.230 ± 0.459%·kyr^−1^) (Fig. 4). The trends for the stress-tolerant and weedy groups of corals are robust and statistically significant.

**Table 1:**
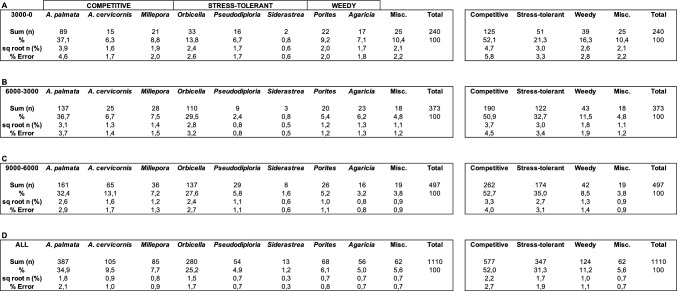
Coral abundances in three time bins (A-C: 3000–0, 6000–3000, 9000–6000 yrs BP) and in total (D). For a listing per core location, see Supplementary Table 4.

## Discussion

The new reef accretion rates from Belize barrier and atoll reefs (average of 3.36 m kyr^−1^) are close to the values calculated based on previous data^26, 27^ and confirm the observation that reefs dominated by branched corals accrete at comparable rates as compared to reefs dominated by massive corals, even though the growth rates of these different morphologies differ by one order of magnitude^36–39^. The latter has been explained by common breakage of branched corals in shallow water and the steady growth of massive corals in somewhat deeper water with lower impacts of physical and biological disturbance^27, 36^. Accretion rates of reef margins in Belize are in the same range as compared to other regions in the western Atlantic (Caribbean average 3.37 ± 1.56 m kyr^-1^), and somewhat lower when compared with data from the Indo-Pacific (Indian Ocean average: 3.82 ± 2.54 m kyr^−1^; Pacific average: 3.90 ± 2.02 m kyr^−1^; Great Barrier Reef average: 4.65 ± 1.10 m kyr^−1^)^40^ and at the lower end of predictions of future sea-level rise by the IPCC (26–98 cm per century or 2.6–9.8 m kyr^−1^)^29^. This would confirm predictions of future reef accretion potential based on carbonate production estimates, which have shown that reefs in Belize, the wider Caribbean, and parts of the Indian Ocean are likely losing the capacity to track predicted rises in future sea level^12^.

The healthy state of coral reefs in the Caribbean has been declining^7, 8^. Phase shifts from stony corals to fleshy algae and from common reef-builders *Orbicella* and *Acropora* to weedy taxa such as *Agaricia* and *Porites* and the increase in abundance of generalists such as *Siderastrea siderea* have been observed^9, 11^. In shelf reefs of Belize, a twentieth century (1980–1990s) phase shift from *A. cervicornis* to *Agaricia tenuifolia* has been documented^41^. White band disease is common in *Acropora*, and the taxon has been shown to be rather vulnerable to anthropogenic stress^42^. Data from the present study suggest that comparable changes have also occurred, at least on longer timescales, during the Holocene. Weedy taxa in Belize reef margins show a statistically significant increase in abundance throughout the Holocene (from 8.5 ± 1.4 to 16.3 ± 2.8%), and stress-tolerant corals exhibit significant decline (from 35.0 ± 3.1 to 21.3 ± 3.3%). No significant change appears to have occurred in the relative abundance of all the competitive coral taxa (Table 1).

At the base of Holocene coral reef successions, lags in reef accretion have been observed that apparently occurred after initial inundation of the reef pedestal. These lag times, which may occur between basal mangrove peat and overlying basal Holocene corals, lasted 2.04–2.19 ka in Belize^26^ and 0.7–2.0 ka in the Great Barrier Reef^37^. A reasonable explanation is the inimical bank water model, based on core data from Lang Bank (St. Croix, NE Caribbean), which underlines the deleterious effect of turbid and nutrient-rich waters created after the initial flooding of subaerially exposed reef banks with soil cover^43, 44^. This effect has been potentially responsible for the fact that carbonate-producing organisms need a certain amount of time to become established on inundated platforms and start what is commonly called the carbonate factory^45, 46^. Observations on St. Croix^47^ have challenged the inimical bank water hypothesis by showing that Holocene reefs had in fact drowned only well after platform inundation and the presumed presence of inimical waters for reasons not entirely clear. But the observation of this study that 86% (12 out of 14 cores) of the basal Holocene corals directly overlying Pleistocene reef pedestal are stress-tolerant, especially to sedimentation (*Pseudodiploria*)^32^, lends strong evidence for the validity of the inimical bank water model for Belize. For comparison, a *Pseudodiploria* sp. coral was also recovered at the very base of the Holocene succession overlying Pleistocene pedestal, recovered at the barrier reef margin at Carrie Bow Cay, Belize^23^. The observation that the relative abundance of weedy taxa has increased throughout the Holocene suggests that fecundity appears to become a more important trait in the high-energy reef margins of Belize as compared to maximized survival rate as in stress-tolerant corals such as *Orbicella*^32, 48^. Likewise, a previous study has shown that the abundance of *Orbicella* sp. in cores from the fore reef at Carrie Bow Cay was relatively high in the older core parts and decreased towards younger (< 2 ka BP) parts where the branched coral *A. cervicornis* predominated^22^. Interestingly, in this study, the relative abundance of the stress-tolerant *Pseudodiploria* has almost tripled (2.4 ± 0.8% to 6.7 ± 1.7%) whereas that of *Orbicella* was divided in half (29.5 ± 3.2% to 13.8 ± 2.6%) from the 6–3 to the 3–0 ka time bins, possibly reflecting a deterioration in water quality as exerted by increased storm-induced and anthropogenic run-off^49^.

Apart from water quality, long-term factors such as the slow-down of sea-level rise and a temperature decrease after the so-called Holocene climate optimum could be responsible for these trends in coral community structure. A decline in the rate of sea-level rise, as observed after 6 ka BP, has diminished accommodation space and, hence, reef accretion. Rate of rise in Holocene sea level and reef accretion rate indeed exhibit a positive correlation (Fig. 3B). Likewise, a mid-to-late Holocene temperature fall has been suggested to be responsible for reef decline in Belize^26^ as well as south Florida^50^. Coral sclerochronology^51^ and vegetation data^52^ from offshore Belize suggest also that warm and wet conditions during early Holocene times were followed by cooler and drier conditions in the mid-late Holocene. There are opposing data and models of the trends in Holocene temperature, described as the Holocene temperature conundrum^53^, such that it is not entirely clear how reef accretion in Belize fits in these patterns. The data of Marcott and others suggest a mid-late temperature fall following the Holocene Climate Optimum^30^, which would fit the vegetation and sclerochronology data from Belize. However, the correlation of our accretion-rate data and the Marcott-anomaly is insignificant (Fig. 3C). The climate model of Osman and others hints to a continuous temperature rise since the last glacial maximum^31^. Reef accretion data of Belize exhibits a significant negative correlation to the Osman-data set (Fig. 3D).

Other than the discussed changes in the abundance of stress-tolerant and weedy corals, competitive taxa, including acroporids and milleporids, do not seem to exhibit considerable change during the Holocene. The abundance of competitive taxa in Belize reef margins was constantly high at approximately 50%. It could be questioned why the abundance has not decreased due to the decrease in accommodation space with lower rates of sea-level rise starting in the mid-late Holocene. Presumably, acroporids and milleporids responded to this by lateral growth, i.e., progradation^26^. However, the data we have assembled also hints to three gaps in the Holocene *A. palmata* record in Belize. Strikingly, two of the *Acropora* gaps in Belize coincide with the two *Acropora* gaps in the Virgin Islands and the wider Caribbean from ca. 5.9–5.1 to 3.0–2.2 ka^34^ (Fig. 5). One *Acropora* gap in Belize is age-equivalent with a potential mass mortality of grazing echinoids in the region around 4 ka (Fig. 5), which might have caused an increase in the abundance of fleshy algae during this time window^35^. It could also be speculated that the mortality was connected to the 4.2 k-event, which likely caused mid-latitude drought in north America and elevated sea surface temperature in tropical oceans^54^. Based on core and outcrop data from the Holocene, studies on the southern Belize shelf and the Enriquillo Valley, Dominican Republic, have provided evidence that the recent well-documented decline in *Acropora cervicornis* was likely unprecedented^41, 55^. The decline in Caribbean acroporids apparently started in the 1950s and 1960s, i.e., before the increase in diseases and bleaching events, most likely due to human population impacts^56^. However, the data from Florida^33^, the Virgin Islands^34^, and Belize barrier and atoll reefs indicate individually and collectively that significant acroporid declines apparently did occur in the pre-Anthropocene era. The results of the present study suggest that there are gaps in the Holocene *A. palmata* record, and, that *A. cervicornis* was twice as abundant during the early as compared to the mid and late Holocene (13.1 ± 1.7% vs. 6.7 ± 1.4% and 6.3 ± 1.7%, respectively), suggesting a deterioration in the environmental conditions for reef development over time, as also indicated by the increase in weedy coral taxa during the Holocene. It cannot be ruled out completely though, that additional age dating would potentially fill the existing acroporid gaps as it did in the case of *A. palmata* in the Florida Reef Tract^57^.

Qualitative observations and anecdotal reports from along the Belize barrier and atoll reef margins clearly suggest a decline in acroporids in general and *A. palmata* in particular during the second half of the 20th Century, exacerbated by the combined effect of the high sea-surface temperatures and the 1998 landfall of Category 5 Hurricane Mitch^58^. A comparison of the trends observed in this study to these recent changes in coral community structure in reef margins of Belize is hampered though, because quantitative monitoring programs either did not collect data on coral composition around the shallow reef margin^59^, or monitored entities such as “branching coral” or “massive coral” that included several undesignated taxa^60^.

In summary, this study has shown that *A. palmata* and *Orbicella* spp. were the most common reef-building corals in Holocene reef margins of Belize barrier and atoll reefs. The abundance of competitive corals including acroporids appears to be relatively constant during the past 9000 years. Stress-tolerant taxa such as *Pseudodiploria* spp. predominate during Holocene reef initiation, and then subsequently decline. Meanwhile, the abundance of weedy taxa including *Agaricia* spp. and *Porites* spp. increases over time. A decline in the abundance of *A. cervicornis* and the existence of repeated gaps in *A. palmata* suggest that the recent demise in acroporids had precedents in the pre-Anthropocene Holocene. Also, reef-accretion rate is comparably low and has been decreasing during the Holocene.

## Methods

The twenty-two cores revisited during this project were collected during four expeditions (1995: 3 cores, 1996: 3 cores, 1998: 5 cores, 2002: 11 cores) using a portable rotary drill with wireline system (Fig. 1). GPS-coordinates for the core locations are listed in Supplementary Table 5. Cores were drilled on reef margins, on or close to the modern reef crest, along the barrier reef and the offshore atolls of Belize in water usually < 1 m deep. The elevation of the core top was estimated relative to mean sea level. Recovery of entire Holocene sections overlying late Pleistocene reef limestone was attempted. Core lengths ranged from 8 to 20 m totaling 215 m of Holocene reef accretion. Core material was stored in NQ core boxes and deposited at the Institute of Geoscience of Goethe University in Frankfurt, Germany.

Cores were analyzed sedimentologically, taphonomically, and rates of reef accretion calculated^26^. Corals in cores were identified to the levels of genus and species (acroporids) using standard guidebooks^61, 62^. Coral abundance was quantified by counting the numbers of drilled coral fragments > 2 cm diameter in core. In total, 1110 coral fragments were analyzed and identified. We excluded fragments with a diameter < 2 cm from our analysis due to the challenge of correctly attributing them to a taxon. Fragments that evidently belonged to the same coral as seen in fitting fracture surfaces were naturally counted as one specimen. This pitfall is usually unproblematic in massive corals. In branched corals, in our case largely *A. cervicornis*, each fragment > 2 cm encountered in core was counted separately. We used nine categories including *A. palmata*, *A. cervicornis*, *Orbicella* spp., *Pseudodiploria* spp., *Porites* spp., *Siderastrea* spp., *Agaricia* spp., *Millepora* spp., and a final catch-all category “miscellaneous coral”. Subsequently, categories were summarized using the live-history-trait concept of Cramer and others including fast-growth competitive (acroporids, milleporids), low-growth and stress-tolerant (*Orbicella*, *Pseudodiploria*, *Siderastrea*), and weedy, high-fecundity (*Agaricia*, *Porites*) corals^32^. The traits were characterized as maximizing growth (competitive), survival (stress-tolerant), and fecundity (weedy). The distribution and availability of age data in the 9 kyr-time period investigated, allowed the definition of three robust time bins during the Holocene (9–6, 6–3, 3–0 kyrs BP) in order to meaningfully estimate temporal change.

Core chronology was established based on U-series and ^14^C-dates of corals (n = 127) and ^14^C-dates of basal mangrove peat (n = 14). Uncertainties are reported as 2-sigma (95% probability). Of the coral data, 69 are from acroporids, 33 from *Orbicella*, 12 from *Pseudodiploria* and 10 from *Porites*, *Millepora*, and *Siderastrea* (Supplementary Table 2). ^14^C-dating was performed by Beta Analytic Inc., Miami, Florida, USA. U-series-dating was done at the GEOMAR, Kiel, Germany, following their published methodology^63^. Reef accretion rate was calculated between dated core sections by considering elevation and age error ranges as well as error propagation^26^. When error propagation exceeded the error range, samples were excluded from further analysis. Accretion rates in core sections dominated by either branched or massive corals, respectively, were compared (Supplementary Table 3). Sections containing both branched and massive corals in similar abundances were excluded from this comparison. Two reconstructions of Holocene SST anomalies^30, 31^ were taken in 500-yr time bins for statistical correlation. Rates of Holocene sea-level rise were estimated in 250-yr time bins based on the ANU_HR_71p230 ice-model^28^ sea-level data (Supplementary Figs. 2, 3). This model was used as it exhibited the best fit to the Belize sea-level data based on a quantitative data-model fit (Supplementary Table 6; Supplementary Figs. 2, 3).

Other statistical methods include correlation analysis as well as Chi-Square and Kruskal–Wallis-tests performed using the PAST software^64^. In order to compare mean values of reef accretion, we used the non-parametric Kruskal–Wallis test (or H-test), because the data were not normally distributed. Regression analysis was done using the OriginPro software package. Correlation analysis was used to compare accretion rates with environmental factors such as rate of sea-level rise and sea-surface temperature anomalies. We applied linear regression to test the temporal trends in reef accretion and in the observed changes in relative abundances of coral taxa composition. Slopes and their uncertainties (m kyr^−1^ and %·kyr^−1^) were calculated based on the principle of least squares to identify significant trends. We used the Poisson distribution as a model for coral count distribution. The uncertainty of the Poisson distribution is given with σ = √µ where µ denotes the mean value of the distribution^65^, which is identified with the number of corals per time bin. The relative abundance of the coral groups was computed and the uncertainties were propagated by Gaussian error propagation accounting for the uncertainty of the number of corals in the given group and of the sum of all corals found in the respective time bin. Uncertainties are reported as 1 standard error (SE). The time uncertainties used, represent the maximum dating errors in the three time bins (9–6 ka: ± 264 yrs; 6–3 ka: ± 220 yrs; 3–0 ka: ± 195 yrs).

## Supplementary Information

Supplementary Information.

## Data Availability

All data generated or analyzed during this study are included in this published article and its supplementary information files.
