# Supplementary material for: 9000 years of change in coral community structure and accretion in Belize reefs, western Atlantic
Source: Sci Rep. 2023 Jul 13;13:11349. doi: 10.1038/s41598-023-38118-5 (PMC10345111; doi:10.1038/s41598-023-38118-5)
Supplement: Supplementary file 1 — Supplementary Information. [file 41598_2023_38118_MOESM1_ESM.pdf]

Supplementary data for

9000 years of change in coral community structure and reef accretion in Belize reefs, western Atlantic

Eberhard Gischler[1*], J. Harold Hudson[2], Anton Eisenhauer[3], Soran Parang[4], Michael Deveaux[5]

[1] Institute of Geosciences, Goethe-University, 60438 Frankfurt am Main, Germany
[2]ReefTech Inc., Miami, Florida 33143, USA
[3]GEOMAR Helmholtz Center of Ocean Research, 24148 Kiel, Germany
[4]University of Ottawa, Department of Earth and Environmental Sciences, Ottawa, ON K1N 6N5, Canada
[5]GSI Helmholtz Center of Heavy Ion Research, 64291 Darmstadt, Germany

*e-mail: gischler@em.uni-frankfurt.de

Supplementary data references

1. Blanchon, P., Eisenhauer, A., Fietzke, J. & Liebetrau, V. Rapid sea-level rise and reef back-stepping at the close of the last interglacial highstand. *Nature* **458**, 881-885 (2009).
2. Gischler, E. & Hudson, J. H. Holocene tropical reef accretion and lagoon sedimentation: a quantitative approach to the influence of sea-level rise, climate, and subsidence (Belize, Maldives, French Polynesia). *The Depositional Record* **5**, 515-539 (2019).
3. Gischler, E. & Hudson, J. H. Holocene development of three isolated carbonate platforms, Belize, Central America. *Marine Geology* **144**, 333-347 (1998).
4. Gischler, E. & Hudson, J. H. Holocene development of the Belize Barrier Reef. *Sedimentary Geology* **164**, 223-236 (2004).
5. Gischler, E. & Lomando, A. J. Isolated carbonate platforms of Belize, Central America: sedimentary facies, late Quaternary history and controlling factors. *Geological Society Special Publication* **178**: 135-146 (2000).
6. Gischler, E. Holocene lagoonal development in isolated carbonate platforms of Belize. *Sedimentary Geology* **159**, 113-132 (2003).
7. Marcott, S. A., Shakun, J. D., Clark, P. U. & Mix, A. C. A reconstruction of regional and global temperature for the past 11,300 years. *Science* **339**, 1198-1201 (2013).
8. Osman, M. B., Tierney, J. E., Zhu, J., Tardif, R., Hakim, G. J., King, J. K. & Poulsen, C. J. Globally resolved surface temperatures since the last glacial maximum. *Nature* **599**, 239-244 (2021).

9. Peltier, W. R., Argus, D. F. & Drummond, R. Space geodesy constrains ice age terminal deglaciation: The global ICE-6G_C (VM5a) model. *Journal of Geophysical Research Solid Earth* **120**, 450-487 (2015). doi:10.1002/2014JB011176
10. Lambeck, K., Rouby, H., Purcell, A., Sun, Y. & Sambridge, M. Sea level and global ice volumes from the last glacial maximum to the Holocene. *PNAS* **111**, 15296-15303 (2014).
11. Milne, G. A. & Peros, M. Data-model comparison of Holocene sea-level change in the circum-Caribbean region. *Global and Planetary Change* **107**, 119-131 (2013). doi:10.1016/j.gloplacha.2013.04.014.
12. Mitrovica, J. X., Forte, A. M. & Simons, M. A reappraisal of postglacial decay times from Richmond Gulf and James Bay, Canada. *Geophysical Journal International* **142** (3), 783e800 (2000). https://doi.org/10.1046/j.1365-246x.2000.00199.x.
13. Gischler, E., Lomando, A. J., Hudson, J. H. & Holmes, C. W. Last interglacial reef growth beneath Belize barrier and isolated platform reefs. *Geology* **28**, 387-390 (2000).
14. Lighty, R. G., Macintyre, I. G. & Stuckenrath, R. *Acropora palmata* reef framework: a reliable indicator of sea level in the western Atlantic for the past 10,000 years. *Coral Reefs* **1**, 125-130 (1982).
15. Wessel, P., Luis, J. F., Uieda, L., Scharroo, R., Wobbe, F., Smith, W. H. F. & Tian, D. The Generic Mapping Tools version 6. *Geochemistry Geophysics Geosystems* **20**, 5556-5564 (2019). https://doi.org/10.1029/2019GC008515

Supplementary Table 1. New U-series age data from Belize barrier and atoll reefs. Delta notation for liability criterion[1].

core	core depth cm	± range cm	material / coral	age BP ky	± ky	min-age ky	max-age ky	U238 ppm	± ppm	Th232 ppb	± ppb	Th230 ppt	± ppt	Th230/Th232 dpm/dpm	± dpm/dpm	U238/Th232 dpm/dpm	± dpm/dpm	Th230/U238 dpm/dpm	± dpm/dpm	Th230exc./U238 dpm/dpm	± dpm/dpm	U234/U238 dpm/dpm	± dpm/dpm	U234/U238initial dpm/dpm	± dpm/dpm	% detrital correction on 230Th/234U act. ratio used for age calculation	U234/U238initial dpm/dpm	± dpm/dpm
**GR 1**	**0,275**	**0,275**	***A. palmata***	**0,087**	**0,008**	**0,079**	**0,095**	**3,119**	**0,013**	**0,737**	**0,004**	**0,050**	**0,004**	**12,62**	**1,11**	**12982,8**	**94,2**	**0,0010**	**0,0001**	**0,0009**	**0,0001**	**1,1456**	**0,0016**	**1,1456**	**0,0016**	5,9	145,6	1,6
**GR 1**	**2,1**	**0,4**	***Porites***	**4,371**	**0,033**	**4,338**	**4,404**	**3,611**	**0,015**	**1,138**	**0,006**	**2,666**	**0,016**	**439,57**	**3,63**	**9735,1**	**68,2**	**0,0452**	**0,0003**	**0,0451**	**0,0003**	**1,1463**	**0,0016**	**1,1481**	**0,0016**	0,2	148,1	1,6
**GR 1**	**3,55**	**0,55**	***A. palmata***	**5,541**	**0,041**	**5,500**	**5,583**	**4,616**	**0,019**	**6,592**	**0,034**	**4,286**	**0,023**	**122,01**	**0,94**	**2148,5**	**14,9**	**0,0568**	**0,0004**	**0,0564**	**0,0004**	**1,1379**	**0,0022**	**1,1401**	**0,0022**	0,6	140,1	2,2
**GR 1**	**5,05**	**0,35**	***A. palmata***	**6,637**	**0,048**	**6,590**	**6,685**	**3,258**	**0,013**	**1,833**	**0,009**	**3,603**	**0,020**	**368,77**	**2,86**	**5451,7**	**37,6**	**0,0676**	**0,0005**	**0,0675**	**0,0005**	**1,1418**	**0,0016**	**1,1445**	**0,0016**	0,2	144,5	1,6
**GR 1**	**6,675**	**0,375**	***Orbicella***	**7,185**	**0,053**	**7,132**	**7,238**	**2,586**	**0,011**	**2,360**	**0,012**	**3,089**	**0,018**	**245,55**	**1,94**	**3361,1**	**23,0**	**0,0731**	**0,0005**	**0,0728**	**0,0005**	**1,1408**	**0,0014**	**1,1437**	**0,0015**	0,3	143,7	1,5
**GR 1**	**8,275**	**0,725**	***A. cervicornis***	**7,260**	**0,052**	**7,209**	**7,312**	**3,428**	**0,014**	**1,468**	**0,008**	**4,138**	**0,022**	**528,95**	**4,10**	**7164,2**	**49,3**	**0,0738**	**0,0005**	**0,0737**	**0,0005**	**1,1432**	**0,0012**	**1,1461**	**0,0012**	0,1	146,1	1,2
**GR 2**	**5,025**	**0,125**	***A. palmata***	**6,322**	**0,045**	**6,277**	**6,367**	**3,530**	**0,014**	**3,279**	**0,017**	**3,740**	**0,020**	**214,04**	**1,63**	**3303,1**	**22,5**	**0,0648**	**0,0004**	**0,0645**	**0,0004**	**1,1451**	**0,0012**	**1,1477**	**0,0013**	0,4	147,7	1,3
**GR 2**	**6,575**	**0,525**	***A. cervicornis***	**7,512**	**0,058**	**7,454**	**7,570**	**3,598**	**0,015**	**12,518**	**0,063**	**4,541**	**0,024**	**68,07**	**0,52**	**881,8**	**6,0**	**0,0772**	**0,0005**	**0,0762**	**0,0005**	**1,1453**	**0,0014**	**1,1484**	**0,0015**	1,1	148,4	1,5
**GR 2**	**9,55**	**0,55**	***A. cervicornis***	**7,459**	**0,054**	**7,406**	**7,513**	**3,344**	**0,014**	**1,748**	**0,009**	**4,139**	**0,022**	**444,24**	**3,42**	**5868,0**	**40,1**	**0,0757**	**0,0005**	**0,0756**	**0,0005**	**1,1416**	**0,0017**	**1,1446**	**0,0017**	0,2	144,6	1,7
**GR 2**	**9,8**	**0,55**	***Pseudodiploria***	**7,451**	**0,054**	**7,396**	**7,505**	**3,035**	**0,012**	**0,578**	**0,003**	**3,744**	**0,021**	**1215,14**	**9,92**	**16101,5**	**116,1**	**0,0755**	**0,0005**	**0,0754**	**0,0005**	**1,1405**	**0,0016**	**1,1435**	**0,0016**	0,1	143,5	1,6
**GR 3**	**0,5**	**0,45**	***A. palmata***	**0,120**	**0,007**	**0,113**	**0,127**	**3,169**	**0,013**	**0,450**	**0,004**	**0,067**	**0,004**	**28,09**	**1,67**	**21622,5**	**217,8**	**0,0013**	**0,0001**	**0,0013**	**0,0001**	**1,1468**	**0,0013**	**1,1469**	**0,0013**	2,7	146,9	1,3
**GR 3**	**6,95**	**0**	**gastropod**	**6,413**	**0,264**	**6,149**	**6,677**	**0,042**	**0,000**	**1,614**	**0,008**	**0,051**	**0,001**	**5,97**	**0,07**	**79,6**	**0,5**	**0,0750**	**0,0008**	**0,0640**	**0,0008**	**1,1471**	**0,0024**	**1,1498**	**0,0025**	12,6	149,8	2,5
**TR 4**	**0,25**	**0,05**	***Millepora***	**0,436**	**0,017**	**0,420**	**0,453**	**1,479**	**0,006**	**0,762**	**0,005**	**0,114**	**0,004**	**28,04**	**1,05**	**5954,8**	**49,2**	**0,0047**	**0,0002**	**0,0046**	**0,0002**	**1,1482**	**0,0015**	**1,1483**	**0,0015**	2,7	148,3	1,5
**TR 4**	**1,35**	**0,05**	***Millepora***	**2,517**	**0,025**	**2,492**	**2,543**	**1,774**	**0,007**	**2,816**	**0,015**	**0,769**	**0,006**	**51,25**	**0,51**	**1932,2**	**13,4**	**0,0265**	**0,0002**	**0,0261**	**0,0002**	**1,1449**	**0,0014**	**1,1459**	**0,0014**	1,5	145,9	1,4
**TR 4**	**3,125**	**0,025**	***Pseudodiploria***	**5,968**	**0,052**	**5,916**	**6,021**	**2,649**	**0,011**	**10,596**	**0,054**	**2,687**	**0,016**	**47,58**	**0,38**	**766,9**	**5,2**	**0,0620**	**0,0004**	**0,0609**	**0,0004**	**1,1453**	**0,0016**	**1,1478**	**0,0016**	1,6	147,8	1,6
**TR 5**	**0,45**	**0,25**	***Orbicella***	**1,084**	**0,014**	**1,069**	**1,098**	**2,484**	**0,010**	**0,174**	**0,004**	**0,460**	**0,006**	**496,08**	**12,69**	**43781,1**	**995,4**	**0,0113**	**0,0001**	**0,0113**	**0,0001**	**1,1439**	**0,0019**	**1,1443**	**0,0019**	0,2	144,3	1,9
**TR 5**	**1,15**	**0,25**	***Orbicella***	**1,191**	**0,014**	**1,177**	**1,205**	**2,545**	**0,011**	**0,233**	**0,004**	**0,517**	**0,006**	**415,63**	**8,34**	**33466,7**	**580,7**	**0,0124**	**0,0001**	**0,0124**	**0,0001**	**1,1406**	**0,0015**	**1,1411**	**0,0015**	0,2	141,1	1,5
**LR 7**	**0,1**	**0,05**	***A. palmata***	**1,464**	**0,014**	**1,450**	**1,478**	**3,115**	**0,013**	**1,902**	**0,010**	**0,787**	**0,006**	**77,61**	**0,75**	**5024,9**	**35,4**	**0,0154**	**0,0001**	**0,0153**	**0,0001**	**1,1465**	**0,0018**	**1,1471**	**0,0018**	1,0	147,1	1,8
**LR 7**	**2,15**	**0,35**	***A. palmata***	**4,397**	**0,035**	**4,362**	**4,432**	**2,856**	**0,012**	**4,234**	**0,022**	**2,142**	**0,013**	**94,92**	**0,79**	**2069,0**	**14,3**	**0,0459**	**0,0003**	**0,0455**	**0,0003**	**1,1507**	**0,0016**	**1,1526**	**0,0017**	0,8	152,6	1,7
**LR 7**	**4,9**	**0**	***Millepora***	**7,479**	**0,085**	**7,393**	**7,564**	**1,098**	**0,004**	**9,837**	**0,050**	**1,401**	**0,009**	**26,73**	**0,23**	**342,4**	**2,3**	**0,0781**	**0,0006**	**0,0755**	**0,0006**	**1,1434**	**0,0015**	**1,1464**	**0,0015**	2,8	146,4	1,5
**LR 7**	**6,3**	**0**	***Pseudodiploria***	**8,086**	**0,073**	**8,013**	**8,158**	**2,456**	**0,010**	**15,913**	**0,080**	**3,346**	**0,017**	**39,45**	**0,30**	**473,5**	**3,2**	**0,0833**	**0,0005**	**0,0815**	**0,0005**	**1,1421**	**0,0025**	**1,1454**	**0,0026**	1,9	145,4	2,6
**LR 10**	**3,075**	**0,075**	***A. palmata***	**0,137**	**0,001**	**0,136**	**0,139**	**3,743**	**0,015**	**0,243**	**0,001**	**0,089**	**0,001**	**68,69**	**0,68**	**47158,0**	**328,6**	**0,0015**	**0,0000**	**0,0014**	**0,0000**	**1,1453**	**0,0016**	**1,1453**	**0,0016**	1,1	145,3	1,6
**LR 10**	**3,775**	**0,075**	***A. cervicornis***	**4,424**	**0,031**	**4,392**	**4,455**	**3,931**	**0,016**	**2,581**	**0,013**	**2,934**	**0,016**	**213,37**	**1,63**	**4673,6**	**31,9**	**0,0457**	**0,0003**	**0,0455**	**0,0003**	**1,1434**	**0,0016**	**1,1452**	**0,0016**	0,4	145,2	1,6
**LR 11**	**0,6**	**0,6**	***Orbicella***	**4,277**	**0,041**	**4,236**	**4,318**	**3,033**	**0,012**	**12,655**	**0,064**	**2,240**	**0,013**	**33,21**	**0,26**	**735,3**	**5,0**	**0,0452**	**0,0003**	**0,0440**	**0,0003**	**1,1469**	**0,0015**	**1,1487**	**0,0015**	2,3	148,7	1,5
**LR 11**	**5,4**	**0,6**	***Orbicella***	**5,250**	**0,040**	**5,210**	**5,290**	**3,433**	**0,014**	**5,863**	**0,030**	**3,046**	**0,016**	**97,46**	**0,75**	**1796,1**	**12,2**	**0,0543**	**0,0004**	**0,0538**	**0,0004**	**1,1446**	**0,0027**	**1,1467**	**0,0027**	0,8	146,7	2,7
**LR 12**	**0,65**	**0,25**	***A. palmata***	**1,990**	**0,018**	**1,972**	**2,008**	**3,775**	**0,016**	**6,727**	**0,034**	**1,305**	**0,007**	**36,41**	**0,28**	**1721,3**	**11,8**	**0,0212**	**0,0001**	**0,0206**	**0,0001**	**1,1453**	**0,0018**	**1,1461**	**0,0018**	2,1	146,1	1,8
**LR 12**	**2,125**	**0,625**	***Pseudodiploria***	**4,569**	**0,033**	**4,536**	**4,603**	**5,532**	**0,023**	**5,379**	**0,027**	**4,267**	**0,023**	**148,85**	**1,15**	**3154,8**	**21,5**	**0,0472**	**0,0003**	**0,0469**	**0,0003**	**1,1430**	**0,0018**	**1,1449**	**0,0018**	0,5	144,9	1,8
**LR 12**	**2,375**	**0,625**	***Porites***	**5,178**	**0,037**	**5,141**	**5,215**	**3,245**	**0,013**	**3,583**	**0,018**	**2,829**	**0,015**	**148,16**	**1,13**	**2778,2**	**19,0**	**0,0533**	**0,0004**	**0,0530**	**0,0004**	**1,1431**	**0,0016**	**1,1452**	**0,0016**	0,5	145,2	1,6
**LR 12**	**5,475**	**0,525**	***Porites***	**6,891**	**0,050**	**6,841**	**6,941**	**3,370**	**0,014**	**5,013**	**0,025**	**3,882**	**0,021**	**145,33**	**1,11**	**2062,5**	**14,0**	**0,0705**	**0,0005**	**0,0700**	**0,0005**	**1,1433**	**0,0014**	**1,1461**	**0,0015**	0,5	146,1	1,5
**LR 13**	**0,45**	**0,35**	***A. palmata***	**2,980**	**0,042**	**2,939**	**3,022**	**4,502**	**0,019**	**25,442**	**0,128**	**2,370**	**0,013**	**17,48**	**0,13**	**542,9**	**3,7**	**0,0322**	**0,0002**	**0,0306**	**0,0002**	**1,1427**	**0,0014**	**1,1439**	**0,0014**	4,3	143,9	1,4
**LR 13**	**8,05**	**0,55**	***gastropod***	**9,272**	**0,093**	**9,179**	**9,366**	**4,034**	**0,017**	**39,301**	**0,200**	**6,315**	**0,032**	**30,15**	**0,23**	**314,9**	**2,2**	**0,0957**	**0,0006**	**0,0930**	**0,0006**	**1,1434**	**0,0020**	**1,1472**	**0,0020**	2,5	147,2	2,0
**LR 14**	**0,475**	**0,475**	***Orbicella***	**1,797**	**0,016**	**1,781**	**1,813**	**5,195**	**0,021**	**7,123**	**0,036**	**1,617**	**0,010**	**42,60**	**0,35**	**2237,6**	**15,3**	**0,0190**	**0,0001**	**0,0186**	**0,0001**	**1,1439**	**0,0019**	**1,1447**	**0,0019**	1,8	144,7	1,9
**LR 14**	**2,675**	**0,325**	***Orbicella***	**6,499**	**0,064**	**6,435**	**6,564**	**2,726**	**0,011**	**19,200**	**0,097**	**3,034**	**0,016**	**29,65**	**0,22**	**435,5**	**3,0**	**0,0681**	**0,0005**	**0,0661**	**0,0004**	**1,1457**	**0,0013**	**1,1484**	**0,0013**	2,5	148,4	1,3
**BBR 1**	**0,325**	**0,325**	***A. palmata***	**-0,008**	**-0,010**	**0,001**	**-0,018**	**2,976**	**0,012**	**1,477**	**0,008**	**0,002**	**0,001**	**0,20**	**0,13**	**6182,1**	**42,4**	**0,0000**	**0,0000**	**-0,0001**	**0,0000**	**1,1432**	**0,0014**	**1,1432**	**0,0014**	366,5	143,2	1,4
**BBR 1**	**2,1**	**0,4**	***Orbicella***	**4,601**	**0,035**	**4,566**	**4,636**	**3,150**	**0,013**	**6,648**	**0,034**	**2,462**	**0,013**	**69,48**	**0,53**	**1453,5**	**9,9**	**0,0478**	**0,0003**	**0,0472**	**0,0003**	**1,1435**	**0,0013**	**1,1454**	**0,0013**	1,1	145,4	1,3
**BBR 1**	**4,85**	**0,15**	***A. palmata***	**6,289**	**0,057**	**6,232**	**6,345**	**2,940**	**0,012**	**16,062**	**0,081**	**3,155**	**0,017**	**36,86**	**0,28**	**561,5**	**3,8**	**0,0656**	**0,0004**	**0,0641**	**0,0004**	**1,1462**	**0,0014**	**1,1488**	**0,0014**	2,0	148,8	1,4
**BBR 1**	**6,425**	**0,425**	***Orbicella***	**6,375**	**0,046**	**6,329**	**6,422**	**2,928**	**0,012**	**4,382**	**0,022**	**3,124**	**0,017**	**133,79**	**1,03**	**2050,0**	**14,0**	**0,0653**	**0,0004**	**0,0648**	**0,0004**	**1,1415**	**0,0014**	**1,1441**	**0,0014**	0,6	144,1	1,4
**BBR 1**	**10,075**	**0,425**	***Siderastrea***	**6,911**	**0,049**	**6,862**	**6,960**	**2,982**	**0,012**	**1,453**	**0,007**	**3,430**	**0,018**	**442,89**	**3,40**	**6296,1**	**42,9**	**0,0703**	**0,0005**	**0,0702**	**0,0005**	**1,1422**	**0,0013**	**1,1450**	**0,0013**	0,2	145,0	1,3
**BBR 1**	**13,5**	**0**	***M. cavernosa***	**7,098**	**0,051**	**7,047**	**7,148**	**2,197**	**0,009**	**0,208**	**0,001**	**2,594**	**0,014**	**2342,79**	**18,59**	**32442,7**	**231,2**	**0,0722**	**0,0005**	**0,0722**	**0,0005**	**1,1442**	**0,0015**	**1,1471**	**0,0015**	0,0	147,1	1,5
**BBR 2**	**0,3**	**0,3**	***A. palmata***	**0,122**	**0,002**	**0,120**	**0,124**	**3,064**	**0,013**	**0,794**	**0,004**	**0,067**	**0,001**	**15,90**	**0,18**	**11838,7**	**80,8**	**0,0013**	**0,0000**	**0,0013**	**0,0000**	**1,1442**	**0,0015**	**1,1442**	**0,0015**	4,7	144,2	1,5
**BBR 2**	**0,9**	**0,3**	***A. palmata***	**1,452**	**0,041**	**1,411**	**1,493**	**1,543**	**0,006**	**9,635**	**0,049**	**0,420**	**0,003**	**8,19**	**0,07**	**491,4**	**3,3**	**0,0167**	**0,0001**	**0,0149**	**0,0001**	**1,1441**	**0,0014**	**1,1447**	**0,0014**	9,2	144,7	1,4
**BBR 2**	**3,275**	**0,275**	***Millepora***	**6,988**	**0,073**	**6,916**	**7,061**	**1,462**	**0,006**	**9,449**	**0,048**	**1,738**	**0,012**	**34,51**	**0,31**	**474,6**	**3,3**	**0,0727**	**0,0006**	**0,0709**	**0,0006**	**1,1445**	**0,0015**	**1,1474**	**0,0015**	2,2	147,4	1,5
**BBR 2**	**4,075**	**0,275**	***A. palmata***	**7,877**	**0,074**	**7,803**	**7,951**	**2,869**	**0,012**	**19,856**	**0,100**	**3,824**	**0,020**	**36,14**	**0,27**	**443,3**	**3,0**	**0,0815**	**0,0005**	**0,0795**	**0,0005**	**1,1439**	**0,0027**	**1,1471**	**0,0027**	2,1	147,1	2,7
**BBR 2**	**4,225**	**0,275**	***Pseudodiploria***	**8,165**	**0,076**	**8,089**	**8,242**	**2,591**	**0,011**	**18,880**	**0,095**	**3,574**	**0,019**	**35,52**	**0,27**	**421,0**	**2,9**	**0,0844**	**0,0006**	**0,0823**	**0,0006**	**1,1430**	**0,0019**	**1,1464**	**0,0020**	2,1	146,4	2,0
**BBR 3**	**0,425**	**0,425**	***Orbicella***	**0,147**	**0,008**	**0,138**	**0,155**	**2,684**	**0,011**	**3,479**	**0,018**	**0,082**	**0,001**	**4,40**	**0,08**	**2366,8**	**16,1**	**0,0019**	**0,0000**	**0,0015**	**0,0000**	**1,1474**	**0,0016**	**1,1474**	**0,0016**	17,0	147,4	1,6
**BBR 3**	**1,075**	**0,425**	***A. palmata***	**3,684**	**0,026**	**3,658**	**3,710**	**4,317**	**0,018**	**1,495**	**0,008**	**2,693**	**0,015**	**337,84**	**2,59**	**8856,8**	**60,3**	**0,0381**	**0,0003**	**0,0380**	**0,0003**	**1,1449**	**0,0016**	**1,1464**	**0,0016**	0,2	146,4	1,6
**BBR 3**	**5,375**	**0,525**	***A. palmata***	**6,679**	**0,048**	**6,631**	**6,727**	**3,572**	**0,015**	**2,423**	**0,012**	**3,996**	**0,022**	**309,44**	**2,38**	**4522,4**	**30,8**	**0,0684**	**0,0005**	**0,0682**	**0,0005**	**1,1475**	**0,0017**	**1,1503**	**0,0017**	0,2	150,3	1,7
**BBR 3**	**17,1**	**0,6**	***A. palmata***	**8,307**	**0,064**	**8,244**	**8,371**	**3,033**	**0,012**	**10,914**	**0,055**	**4,213**	**0,022**	**72,43**	**0,55**	**852,6**	**5,8**	**0,0850**	**0,0006**	**0,0839**	**0,0006**	**1,1445**	**0,0016**	**1,1480**	**0,0016**	1,0	148,0	1,6
**BBR 4**	**0,75**	**0,45**	***Pseudodiploria***	**1,968**	**0,022**	**1,946**	**1,990**	**0,290**	**0,001**	**0,749**	**0,004**	**0,100**	**0,001**	**25,10**	**0,20**	**1187,4**	**8,1**	**0,0211**	**0,0002**	**0,0204**	**0,0002**	**1,1464**	**0,0015**	**1,1472**	**0,0016**	3,0	147,2	1,6
**BBR 4**	**2,575**	**0,425**	***Orbicella***	**5,170**	**0,043**	**5,128**	**5,213**	**2,429**	**0,010**	**6,544**	**0,033**	**2,137**	**0,013**	**61,28**	**0,50**	**1138,6**	**7,8**	**0,0538**	**0,0004**	**0,0531**	**0,0004**	**1,1470**	**0,0012**	**1,1492**	**0,0012**	1,2	149,2	1,2
**BBR 4**	**5,15**	**0,65**	***A. palmata***	**5,538**	**0,048**	**5,490**	**5,586**	**2,813**	**0,011**	**12,296**	**0,063**	**2,660**	**0,014**	**40,59**	**0,31**	**701,7**	**4,8**	**0,0578**	**0,0004**	**0,0566**	**0,0004**	**1,1454**	**0,0016**	**1,1477**	**0,0016**	1,8	147,7	1,6
**BBR 4**	**7,7**	**0,2**	***A. palmata***	**6,990**	**0,049**	**6,941**	**7,039**	**2,965**	**0,012**	**1,313**	**0,007**	**3,452**	**0,018**	**493,17**	**3,75**	**6926,1**	**47,3**	**0,0712**	**0,0005**	**0,0711**	**0,0005**	**1,1437**	**0,0014**	**1,1465**	**0,0015**	0,2	146,5	1,5
**BBR 4**	**11,6**	**0,4**	***A. cervicornis***	**9,002**	**0,074**	**8,928**	**9,076**	**3,404**	**0,014**	**19,288**	**0,098**	**5,127**	**0,027**	**49,88**	**0,38**	**541,4**	**3,7**	**0,0921**	**0,0006**	**0,0905**	**0,0006**	**1,1434**	**0,0014**	**1,1471**	**0,0014**	1,5	147,1	1,4
**BBR 5**	**2**	**0,5**	***Pseudodiploria***	**0,645**	**0,008**	**0,637**	**0,652**	**2,740**	**0,011**	**2,442**	**0,012**	**0,313**	**0,002**	**24,03**	**0,21**	**3442,1**	**23,4**	**0,0070**	**0,0001**	**0,0067**	**0,0001**	**1,1473**	**0,0014**	**1,1476**	**0,0014**	3,1	147,6	1,4
**BBR 5**	**3,875**	**0,625**	***Orbicella***	**5,247**	**0,041**	**5,206**	**5,288**	**4,130**	**0,017**	**11,341**	**0,057**	**3,682**	**0,020**	**60,92**	**0,46**	**1117,1**	**7,6**	**0,0545**	**0,0004**	**0,0538**	**0,0004**	**1,1455**	**0,0015**	**1,1477**	**0,0015**	1,2	147,7	1,5
**BBR 5**	**8,25**	**0,725**	***Orbicella***	**7,066**	**0,057**	**7,009**	**7,123**	**2,784**	**0,011**	**9,911**	**0,050**	**3,312**	**0,019**	**62,69**	**0,49**	**861,6**	**5,9**	**0,0728**	**0,0005**	**0,0717**	**0,0005**	**1,1444**	**0,0014**	**1,1473**	**0,0015**	1,2	147,3	1,5
**BBR 5**	**13,925**	**0,325**	***Pseudodiploria***	**8,521**	**0,080**	**8,441**	**8,601**	**2,994**	**0,012**	**22,922**	**0,117**	**4,311**	**0,023**	**35,29**	**0,27**	**400,7**	**2,7**	**0,0881**	**0,0006**	**0,0859**	**0,0006**	**1,1452**	**0,0015**	**1,1487**	**0,0015**	2,1	148,7	1,5
**BBR 6**	**0,625**	**0,525**	***Porites***	**0,083**	**0,004**	**0,080**	**0,087**	**3,191**	**0,013**	**1,614**	**0,008**	**0,052**	**0,001**	**6,06**	**0,14**	**6067,0**	**41,2**	**0,0010**	**0,0000**	**0,0009**	**0,0000**	**1,1443**	**0,0013**	**1,1443**	**0,0013**	12,4	144,3	1,3
**BBR 6**	**0,875**	**0,525**	***A. palmata***	**1,105**	**0,020**	**1,085**	**1,125**	**2,589**	**0,011**	**7,422**	**0,037**	**0,518**	**0,004**	**13,11**	**0,12**	**1070,2**	**7,3**	**0,0122**	**0,0001**	**0,0114**	**0,0001**	**1,1452**	**0,0014**	**1,1457**	**0,0014**	5,7	145,7	1,4
**BBR 6**	**2,1**	**0,4**	***A. palmata***	**4,202**	**0,030**	**4,172**	**4,233**	**3,012**	**0,012**	**3,636**	**0,019**	**2,148**	**0,012**	**110,85**	**0,85**	**2541,8**	**17,4**	**0,0436**	**0,0003**	**0,0433**	**0,0003**	**1,1450**	**0,0014**	**1,1467**	**0,0014**	0,7	146,7	1,4
**BBR 6**	**6,8**	**0,4**	***A. palmata***	**5,146**	**0,040**	**5,106**	**5,186**	**3,699**	**0,015**	**7,540**	**0,038**	**3,221**	**0,018**	**80,15**	**0,63**	**1504,9**	**10,2**	**0,0533**	**0,0004**	**0,0527**	**0,0004**	**1,1435**	**0,0014**	**1,1456**	**0,0014**	0,9	145,6	1,4
**BBR 6**	**11,15**	**0,65**	***A. palmata***	**6,420**	**0,048**	**6,372**	**6,468**	**3,465**	**0,014**	**7,650**	**0,039**	**3,733**	**0,020**	**91,57**	**0,70**	**1389,6**	**9,5**	**0,0659**	**0,0004**	**0,0653**	**0,0004**	**1,1418**	**0,0016**	**1,1444**	**0,0017**	0,8	144,4	1,7
**BBR 7**	**0,55**	**0,55**	***A. palmata***	**0,119**	**0,005**	**0,114**	**0,125**	**3,154**	**0,013**	**2,312**	**0,012**	**0,074**	**0,002**	**5,98**	**0,15**	**4185,3**	**28,7**	**0,0014**	**0,0000**	**0,0012**	**0,0000**	**1,1441**	**0,0012**	**1,1442**	**0,0012**	12,5	144,2	1,2
**BBR 7**	**0,95**	**0,55**	***A. palmata***	**1,035**	**0,008**	**1,028**	**1,043**	**3,168**	**0,013**	**0,304**	**0,002**	**0,561**	**0,003**	**345,88**	**2,80**	**31929,1**	**221,0**	**0,0108**	**0,0001**	**0,0108**	**0,0001**	**1,1437**	**0,0014**	**1,1442**	**0,0014**	0,2	144,2	1,4
**BBR 7**	**4,95**	**0,45**	***Agaricia***	**5,188**	**0,047**	**5,141**	**5,236**	**3,363**	**0,014**	**15,643**	**0,079**	**2,984**	**0,016**	**35,79**	**0,27**	**659,6**	**4,5**	**0,0543**	**0,0004**	**0,0529**	**0,0004**	**1,1424**	**0,0014**	**1,1445**	**0,0015**	2,1	144,5	1,5
**BBR 7**	**8**	**0,5**	***A. cervicornis***	**6,309**	**0,046**	**6,263**	**6,356**	**3,348**	**0,014**	**4,446**	**0,023**	**3,543**	**0,020**	**149,51**	**1,16**	**2309,9**	**15,8**	**0,0647**	**0,0004**	**0,0643**	**0,0004**	**1,1443**	**0,0016**	**1,1468**	**0,0016**	0,5	146,8	1,6
**BBR 8**	**0,725**	**0,575**	***A. palmata***	**1,670**	**0,013**	**1,657**	**1,682**	**3,162**	**0,013**	**1,626**	**0,008**	**0,907**	**0,005**	**104,69**	**0,84**	**5966,6**	**40,9**	**0,0175**	**0,0001**	**0,0174**	**0,0001**	**1,1460**	**0,0015**	**1,1467**	**0,0015**	0,7	146,7	1,5
**BBR 8**	**9,75**	**0,7**	***A. palmata***	**6,252**	**0,044**	**6,208**	**6,297**	**2,926**	**0,012**	**0,629**	**0,003**	**3,054**	**0,017**	**911,25**	**7,04**	**14275,4**	**97,8**	**0,0638**	**0,0004**	**0,0638**	**0,0004**	**1,1434**	**0,0015**	**1,1459**	**0,0015**	0,1	145,9	1,5
**BBR 8**	**13,8**	**0,3**	***A. palmata***	**6,423**	**0,045**	**6,378**	**6,468**	**3,184**	**0,013**	**2,511**	**0,013**	**3,409**	**0,018**	**254,71**	**1,92**	**3889,4**	**26,5**	**0,0655**	**0,0004**	**0,0653**	**0,0004**	**1,1403**	**0,0013**	**1,1429**	**0,0013**	0,3	142,9	1,3
**BBR 8**	**15,9**	**0,6**	***Pseudodiploria***	**7,320**	**0,065**	**7,255**	**7,385**	**2,667**	**0,011**	**16,066**	**0,081**	**3,303**	**0,017**	**38,58**	**0,29**	**509,2**	**3,5**	**0,0758**	**0,0005**	**0,0740**	**0,0005**	**1,1428**	**0,0013**	**1,1458**	**0,0013**	1,9	145,8	1,3
**BBR 8**	**18,5**	**0,5**	***A. cervicornis***	**7,706**	**0,055**	**7,651**	**7,762**	**3,105**	**0,013**	**5,278**	**0,027**	**3,992**	**0,021**	**141,93**	**1,07**	**1804,9**	**12,3**	**0,0786**	**0,0005**	**0,0781**	**0,0005**	**1,1448**	**0,0016**	**1,1480**	**0,0016**	0,5	148,0	1,6
**BBR 9**	**0,525**	**0,525**	***A. palmata***	**0,336**	**0,004**	**0,332**	**0,341**	**3,387**	**0,014**	**1,490**	**0,008**	**0,201**	**0,002**	**25,35**	**0,27**	**6975,3**	**47,5**	**0,0036**	**0,0000**	**0,0035**	**0,0000**	**1,1451**	**0,0014**	**1,1452**	**0,0014**	3,0	145,2	1,4
**BBR 9**	**11,45**	**0,55**	***Porites***	**6,777**	**0,050**	**6,727**	**6,827**	**3,310**	**0,014**	**8,567**	**0,043**	**3,766**	**0,019**	**82,48**	**0,62**	**1185,3**	**8,1**	**0,0696**	**0,0005**	**0,0688**	**0,0005**	**1,1429**	**0,0015**	**1,1457**	**0,0015**	0,9	145,7	1,5
**BBR 9**	**13**	**0,5**	***Orbicella***	**7,071**	**0,050**	**7,022**	**7,121**	**2,692**	**0,011**	**1,956**	**0,010**	**3,164**	**0,017**	**303,46**	**2,31**	**4221,0**	**28,7**	**0,0719**	**0,0005**	**0,0717**	**0,0005**	**1,1407**	**0,0013**	**1,1436**	**0,0014**	0,2	143,6	1,4
**BBR 10**	**0,45**	**0,4**	***A. palmata***	**0,099**	**0,002**	**0,097**	**0,101**	**3,135**	**0,013**	**0,393**	**0,002**	**0,055**	**0,001**	**26,26**	**0,42**	**24466,6**	**168,6**	**0,0011**	**0,0000**	**0,0010**	**0,0000**	**1,1467**	**0,0014**	**1,1467**	**0,0014**	2,9	146,7	1,4
**BBR 10**	**5,625**	**0,375**	***A. palmata***	**7,037**	**0,050**	**6,986**	**7,087**	**3,223**	**0,013**	**1,199**	**0,006**	**3,773**	**0,020**	**590,50**	**4,56**	**8247,4**	**56,5**	**0,0716**	**0,0005**	**0,0715**	**0,0005**	**1,1429**	**0,0013**	**1,1458**	**0,0013**	0,1	145,8	1,3

Supplementary Table 2. Compilation ^{14}C- and U-series age dates of corals and basal peats from Belize barrier and atoll reefs, including sea-level data.

core	*coral*	core depth (m)	error (m)	age yrs cal BP	error yrs	min. subs. (m)	corr. depth (m)	age yrs cal BP	final depth (m)	depth of core top	depth below SL (m)	acrr.-rate (m/kyr)	error prop.
GR 1	*Acropora*	0,275	0,275	87	8	0,003	0,278	87	-0,278	0	**-0,278**	0,218	0,212
GR 1	*Acropora*	0,875	0,275	2835	135	0,113	0,988	2835	-0,988	0	**-0,988**	0,798	0,527
GR 1	*Porites*	2,1	0,4	4371	33	0,175	2,275	4371	-2,275	0	**-2,275**	0,512	1,355
GR 1	*Acropora*	2,45	0,4	5055	215	0,202	2,652	5055	-2,652	0	**-2,652**	2,263	3,147
GR 1	*Acropora*	3,55	0,55	5541	41	0,222	3,772	5541	-3,772	0	**-3,772**	1,369	0,932
GR 1	*Acropora*	5,05	0,35	6637	48	0,265	5,315	6637	-5,315	0	**-5,315**	3,409	16,593
GR 1	*Acropora*	5,35	0,35	6725	175	0,269	5,619	6725	-5,619	0	**-5,619**	2,88	3,004
GR 1	*Orbicella*	6,675	0,375	7185	53	0,287	6,962	7185	-6,962	0	**-6,962**		
GR 1	*Orbicella*	8,225	0,725	7045	170	0,282	8,507	7045	-8,507	0	**-8,507**	0,233	6,984
GR 1	*A. cervicornis*	8,275	0,725	7260	52	0,29	8,565	7260	-8,565	0	**-8,565**		
GR 2	*Orbicella*	4,625	0,125	5575	135	0,223	4,848	5575	-4,848	0	**-4,848**	0,535	0,464
GR 2	*Acropora*	5,025	0,125	6322	45	0,253	5,278	6322	-5,278	0	**-5,278**	1,303	0,659
GR 2	*A. cervicornis*	6,575	0,525	7512	58	0,3	6,875	7512	-6,875	0	**-6,875**		
GR 2	*A. cervicornis*	9,55	0,55	7459	54	0,298	9,848	7459	-9,848	0	**-9,848**		
GR 2	*Pseudodiploria*	9,8	0,55	7451	54	0,298	10,098	7451	-10,098	0	**-10,098**	4,94	5,265
GR 2	*Pseudodiploria*	11,45	0,25	7785	140	0,311	11,761	7785	-11,761	0	**-11,761**		
GR 3	*Acropora*	0,5	0,45	120	7	0,005	0,505	120	-0,505	0	**-0,505**	0,135	0,355
GR 3	*Acropora*	0,85	0,45	2720	170	0,109	0,959	2720	-0,959	0	**-0,959**	1,83	2,263
GR 3	*Acropora*	2,25	0,65	3485	175	0,139	2,389	3485	-2,389	0	**-2,389**	1,605	0,463
GR 3	gastropod	6,95	0	6413	264	0,257	7,207	6413	-7,207	0	**-7,207**		
TR 4	*Millepora*	0,25	0,05	436	17	0,017	0,267	436	-0,267	0	**-0,267**	0,922	0,436
TR 4	*Orbicella*	0,65	0,05	870	80	0,035	0,685	870	-0,685	0	**-0,685**	0,425	0,088
TR 4	*Millepora*	1,35	0,05	2517	25	0,101	1,451	2517	-1,451	0	**-1,451**	0,665	0,095
TR 4	*Acropora*	3,025	0,025	5035	220	0,201	3,226	5035	-3,226	0	**-3,226**	0,107	0,085
TR 4	*Pseudodiploria*	3,125	0,025	5968	52	0,239	3,364	5968	-3,364	0	**-3,364**		
TR 5	*Orbicella*	0,45	0,25	1084	14	0,043	0,493	1084	-0,493	-0,9	**-1,393**	6,542	6,385
TR 5	*Orbicella*	1,15	0,25	1191	14	0,048	1,198	1191	-1,198	-0,9	**-2,098**	2,509	3,132
TR 5	*Acropora*	1,85	0,25	1470	135	0,059	1,909	1470	-1,909	-0,9	**-2,809**		
LR 7	*Acropora*	0,1	0,05	1464	14	0,059	0,159	1464	-0,159	-0,9	**-1,059**	0,36	0,065
LR 7	*Orbicella*	0,95	0,05	3825	135	0,153	1,103	3825	-1,103	-0,9	**-2,003**	2,098	1,323
LR 7	*Acropora*	2,15	0,35	4397	35	0,176	2,326	4397	-2,326	-0,9	**-3,226**	5,952	11,062
LR 7	*Acropora*	3,15	0,05	4565	210	0,183	3,333	4565	-3,333	-0,9	**-4,233**	16,667	111,389
LR 7	*Pseudodiploria*	4,15	0,05	4625	185	0,185	4,335	4625	-4,335	-0,9	**-5,235**	0,263	0,042
LR 7	*Millepora*	4,9	0	7479	85	0,299	5,199	7479	-5,199	-0,9	**-6,099**	2,306	0,6
LR 7	*Pseudodiploria*	6,3	0	8086	73	0,323	6,623	6885	-6,623	-0,9	**-7,523**		
LR 10	*Acropora*	3,075	0,075	137	1	0,005	3,08	137	-3,08	-0,3	**-3,38**	0,163	0,036
LR 10	*A. cervicornis*	3,775	0,075	4424	31	0,177	3,952	4424	-3,952	-0,3	**-4,252**	4,949	1,778
LR 10	*Orbicella*	5,7	0,3	4813	33	0,193	5,893	4813	-5,893	-0,3	**-6,193**		
LR 11	*Orbicella*	0,6	0,6	4277	41	0,171	0,771	4277	-0,771	-0,6	**-1,371**	4,933	1,644
LR 11	*Orbicella*	5,4	0,6	5250	40	0,21	5,61	4675	-5,61	-0,6	**-6,21**		
LR 12	*Acropora*	0,65	0,25	1990	18	0,08	0,73	1990	-0,73	-0,9	**-1,63**	0,572	0,351
LR 12	*Pseudodiploria*	2,125	0,625	4569	33	0,183	2,308	4569	-2,308	-0,9	**-3,208**	0,411	2,1
LR 12	*Porites*	2,375	0,625	5178	37	0,207	2,582	5178	-2,582	-0,9	**-3,482**	1,81	0,763
LR 12	*Porites*	5,475	0,525	6891	50	0,276	5,751	6891	-5,751	-0,9	**-6,651**	4,035	4,526
LR 12	*Pseudodiploria*	6,5	0,1	7145	80	0,286	6,786	7145	-6,786	-0,9	**-7,686**		
LR 13	*Acropora*	0,45	0,35	2980	42	0,119	0,569	2980	-0,569	-1,2	**-1,769**	6,195	3,972
LR 13	*Orbicella*	3,95	0,55	3545	175	0,142	4,092	3545	-4,092	-1,2	**-5,292**	0,716	0,226
LR 13	gastropod	8,05	0,55	9272	93	0,371	8,421	9272	-8,421	-1,2	**-9,621**		
LR 14	*Orbicella*	0,475	0,475	1797	16	0,072	0,547	1797	-0,547	-1,2	**-1,747**	0,468	0,178
LR 14	*Orbicella*	2,675	0,325	6499	64	0,26	2,935	6499	-2,935	-1,2	**-4,135**		
BBR 1	*Acropora*	0,325	0,325	0	0	0	0,325	0	-0,325	-0,3	**-0,625**	0,386	0,161
BBR 1	*Orbicella*	2,1	0,4	4601	35	0,184	2,284	4601	-2,284	-0,3	**-2,584**	1,095	3,719
BBR 1	*Acropora*	2,4	0,4	4875	165	0,195	2,595	4875	-2,595	-0,3	**-2,895**	1,733	0,661
BBR 1	*Acropora*	4,85	0,15	6289	57	0,252	5,102	6289	-5,102	-0,3	**-5,402**	18,314	28,62
BBR 1	*Orbicella*	6,425	0,425	6375	46	0,255	6,68	6375	-6,68	-0,3	**-6,98**	26,071	78,638
BBR 1	*Orbicella*	8,25	0,1	6445	145	0,258	8,508	6445	-8,508	-0,3	**-8,808**	3,916	2,757
BBR 1	*Siderastrea*	10,075	0,425	6911	49	0,276	10,351	6911	-10,351	-0,3	**-10,651**	18,316	12,067
BBR 1	*Orbicella*	13,5	0	7098	51	0,284	13,784	7098	-13,784	-0,3	**-14,084**	1,762	1,133
BBR 1	*Orbicella*	13,9	0	7325	95	0,293	14,193	7325	-14,193	-0,3	**-14,493**		
BBR 2	*Acropora*	0,3	0,3	122	2	0,005	0,305	122	-0,305	-0,3	**-0,605**	0,451	0,466
BBR 2	*Acropora*	0,9	0,3	1452	41	0,058	0,958	1452	-0,958	-0,3	**-1,258**	0,352	0,194
BBR 2	*Acropora*	2,15	0,3	5005	215	0,2	2,35	5005	-2,35	-0,3	**-2,65**	0,567	0,372
BBR 2	*Millepora*	3,275	0,275	6988	73	0,28	3,555	6988	-3,555	-0,3	**-3,855**	0,9	0,767
BBR 2	*Acropora*	4,075	0,275	7877	74	0,315	4,39	6675	-4,39	-0,3	**-4,69**	0,54	2,181
BBR 2	*Pseudodiploria*	4,225	0,275	8165	76	0,327	4,552	8165	-4,552	-0,3	**-4,852**		
BBR 3	*Orbicella*	0,425	0,425	147	8	0,006	0,431	147	-0,431	-0,9	**-1,331**	0,184	0,242
BBR 3	*Acropora*	1,075	0,425	3684	26	0,147	1,222	3684	-1,222	-0,9	**-2,122**	0,893	0,385
BBR 3	*Acropora*	3,725	0,525	6650	190	0,266	3,991	6650	-3,991	-0,9	**-4,891**	56,897	503,151
BBR 3	*Acropora*	5,375	0,525	6679	48	0,267	5,642	6679	-5,642	-0,9	**-6,542**	2,851	1,563
BBR 3	*Acropora*	8,3	0,5	7705	155	0,308	8,608	7705	-8,608	-0,9	**-9,508**	14,62	7,145
BBR 3	*Acropora*	17,1	0,6	8307	64	0,332	17,432	8307	-17,432	-0,9	**-18,332**		
BBR 4	*Pseudodiploria*	0,75	0,45	1968	22	0,079	0,829	1968	-0,829	-0,6	**-1,429**	1,006	0,839
BBR 4	*Acropora*	2,025	0,425	3235	165	0,129	2,154	3235	-2,154	-0,6	**-2,754**	0,284	0,47
BBR 4	*Orbicella*	2,575	0,425	5170	43	0,207	2,782	5170	-2,782	-0,6	**-3,382**	6,997	4,652
BBR 4	*Acropora*	5,15	0,65	5538	48	0,222	5,372	5538	-5,372	-0,6	**-5,972**	1,213	1,284
BBR 4	*Acropora*	6,45	0,45	6610	180	0,264	6,714	6610	-6,714	-0,6	**-7,314**	3,289	3,693
BBR 4	*Acropora*	7,7	0,2	6990	49	0,28	7,98	6990	-7,98	-0,6	**-8,58**	2,626	1,409
BBR 4	*Acropora*	9,525	0,375	7685	105	0,307	9,832	7685	-9,832	-0,6	**-10,432**	3,618	2,901
BBR 4	*Acropora*	11,2	0,4	8148	52	0,326	11,526	8148	-11,526	-0,6	**-12,126**	0,468	1,006
BBR 4	*A. cervicornis*	11,6	0,4	9002	74	0,36	11,96	9002	-11,96	-0,6	**-12,56**		
BBR 5	*Pseudodiploria*	2	0,5	645	8	0,026	2,026	645	-2,026	-0,9	**-2,926**	1,159	3,295
BBR 5	*Acropora*	2,4	0,5	990	110	0,04	2,44	990	-2,44	-0,9	**-3,34**	0,346	0,277
BBR 5	*Orbicella*	3,875	0,625	5247	41	0,21	4,085	5247	-4,085	-0,9	**-4,985**	3,378	2,153
BBR 5	*Acropora*	6,875	0,625	6135	155	0,245	7,12	6135	-7,12	-0,9	**-8,02**	1,477	1,786
BBR 5	*Orbicella*	8,25	0,725	7066	57	0,283	8,533	7066	-8,533	-0,9	**-9,433**	9,386	5,196
BBR 5	*Orbicella*	13,825	0,325	7660	160	0,306	14,131	7660	-14,131	-0,9	**-15,031**	0,116	0,787
BBR 5	*Pseudodiploria*	13,925	0,325	8521	80	0,341	14,266	8521	-14,266	-0,9	**-15,166**		
BBR 6	*Porites*	0,625	0,525	83	4	0,003	0,628	83	-0,628	-0,6	**-1,228**	0,245	1,033
BBR 6	*Acropora*	0,875	0,525	1105	20	0,044	0,919	1105	-0,919	-0,6	**-1,519**	0,396	0,305
BBR 6	*Acropora*	2,1	0,4	4202	30	0,168	2,268	4202	-2,268	-0,6	**-2,868**	6,438	9,803
BBR 6	*Acropora*	3,6	0,5	4435	185	0,177	3,777	4435	-3,777	-0,6	**-4,377**	4,501	2,69
BBR 6	*Acropora*	6,8	0,4	5146	40	0,206	7,006	5146	-7,006	-0,6	**-7,606**	1,883	1,932
BBR 6	*Orbicella*	8,05	0,45	5810	190	0,232	8,282	5810	-8,282	-0,6	**-8,882**	0,78	2,418
BBR 6	*Orbicella*	8,4	0,45	6259	48	0,25	8,65	6259	-8,65	-0,6	**-9,25**	17,081	17,017
BBR 6	*Acropora*	11,15	0,65	6420	48	0,257	11,407	6420	-11,407	-0,6	**-12,007**	3,085	3,088
BBR 6	*Orbicella*	12,825	0,675	6963	66	0,279	13,104	6963	-13,104	-0,6	**-13,704**		
BBR 7	*Acropora*	0,55	0,55	119	5	0,005	0,555	119	-0,555	-0,6	**-1,155**	0,437	1,207
BBR 7	*Acropora*	0,95	0,55	1035	8	0,041	0,991	1035	-0,991	-0,6	**-1,591**	0,437	0,294
BBR 7	*Acropora*	2,55	0,45	4695	165	0,188	2,738	4695	-2,738	-0,6	**-3,338**	4,868	3,919
BBR 7	*Agaricia*	4,95	0,45	5188	47	0,208	5,158	5188	-5,158	-0,6	**-5,758**	2,492	1,95
BBR 7	*Orbicella*	6,6	0,3	5850	170	0,234	6,834	5850	-6,834	-0,6	**-7,434**	3,05	3,178
BBR 7	*A. cervicornis*	8	0,5	6309	46	0,252	8,252	6309	-8,252	-0,6	**-8,852**	1,01	2,87
BBR 7	*Orbicella*	8,4	0,5	6705	89	0,268	8,668	6705	-8,668	-0,6	**-9,268**		
BBR 8	*Acropora*	0,725	0.575	1670	13	0,067	0,792	1670	-0,792	-0,3	**-1,092**	1,034	0,99
BBR 8	*Acropora*	2,075	0,575	2975	195	0,119	2,194	2975	-2,194	-0,3	**-2,494**	2,025	0,666
BBR 8	*Acropora*	8,2	0,7	6000	170	0,24	8,44	6000	-8,44	-0,3	**-8,74**	6,151	10,779
BBR 8	*Acropora*	9,75	0,7	6252	44	0,25	10	6252	-10	-0,3	**-10,3**	23,684	18,175
BBR 8	*Acropora*	13,8	0,3	6423	45	0,257	14,057	6423	-14,057	-0,3	**-14,357**	1,587	1,716
BBR 8	*Orbicella*	14,7	0,3	6990	190	0,28	14,98	6990	-14,98	-0,3	**-15,28**	3,636	5,537
BBR 8	*Pseudodiploria*	15,9	0,6	7320	65	0,293	16,193	7320	-16,193	-0,3	**-16,493**	6,736	4,944
BBR 8	*A. cervicornis*	18,5	0,5	7706	55	0,308	18,808	7706	-18,808	-0,3	**-19,108**	0,903	2,074
BBR 8	*Orbicella*	19	0,5	8260	110	0,33	19,33	8260	-19,33	-0,3	**-19,63**		
BBR 9	*Acropora*	0,525	0,525	336	4	0,013	0,538	336	-0,538	-1,2	**-1,738**	6,019	5,173
BBR 9	*Acropora*	2,475	0,525	660	100	0,026	2,501	660	-2,501	-1,2	**-3,701**	1,832	0,351
BBR 9	*Acropora*	11,05	0,55	5340	210	0,214	11,264	5340	-11,264	-1,2	**-12,464**	0,278	0,816
BBR 9	*Porites*	11,45	0,55	6777	50	0,271	11,721	6777	-11,721	-1,2	**-12,921**	5,272	5,365
BBR 9	*Orbicella*	13	0,5	7071	50	0,283	13,283	7071	-13,283	-1,2	**-14,483**		
BBR 9	*Orbicella*	15,625	0,425	7070	170	0,283	15,908	7070	-15,908	-1,2	**-17,108**	3,222	2,02
BBR 9	*Orbicella*	18,75	0,6	8040	120	0,322	19,072	8040	-19,072	-1,2	**-20,272**		
BBR 10	*Acropora*	0,45	0,4	99	2	0,004	0,454	99	-0,454	-0,3	**-0,754**	0,335	0,196
BBR 10	*Acropora*	2,225	0,575	5390	180	0,216	2,441	5390	-2,441	-0,3	**-2,741**	2,108	1,913
BBR 10	*Acropora*	3,975	0,275	6220	170	0,249	4,224	6220	-4,224	-0,3	**-4,524**	2,02	1,339
BBR 10	*Acropora*	5,625	0,375	7037	50	0,281	5,906	7037	-5,906	-0,3	**-6,206**	3,073	2,508
BBR 10	*Acropora*	6,925	0,225	7460	100	0,298	7,223	7460	-7,223	-0,3	**-7,523**		
BBR 11	*Acropora*	2	0,55	5340	110	0,214	2,214	5340	-2,214	-0,3	**-2,514**	2,025	1,22
BBR 11	*Acropora*	4,4	0,4	6525	135	0,261	4,661	6525	-4,661	-0,3	**-4,961**	5,106	4,693
BBR 11	*Acropora*	6,8	0,325	6995	155	0,28	7,08	6995	-7,08	-0,3	**-7,38**		
		below SL:					below SL:						
TR 8	basal peat	3,8	0	6650	125	0,266	4,066	6650	-4,066	0	**-4,066**		
TR 9	basal peat	2,8	0	6208	98	0,248	3,048	6208	-3,048	0	**-3,048**		
LR 10	basal peat	6,5	0	6848	133	0,274	6,774	6848	-6,774	0	**-6,774**		
LR 13	basal peat	10	0	7478	78	0,299	10,299	7478	-10,299	0	**-10,299**		
LR 14	basal peat	7	0	7295	90	0,292	7,292	7295	-7,292	0	**-7,292**		
GL 5	basal peat	18,1	0	8765	235	0,351	18,451	8765	-18,451	0	**-18,451**		
GL 7	basal peat	15,6	0	8470	140	0,339	15,939	8470	-15,939	0	**-15,939**		
GL 9	basal peat	17,3	0	9400	280	0,376	17,676	9400	-17,676	0	**-17,676**		
TL 4	basal peat	5,3	0	6395	105	0,256	5,556	6395	-5,556	0	**-5,556**		
TL 6	basal peat	5,7	0	5315	255	0,213	5,913	5315	-5,913	0	**-5,913**		
TL 7	basal peat	9,3	0	6840	180	0,274	9,574	6840	-9,574	0	**-9,574**		
TL 12	basal peat	3,3	0	2840	110	0,114	3,414	2840	-3,414	0	**-3,414**		
LL 5	basal peat	7,4	0	7800	140	0,312	7,712	7800	-7,712	0	**-7,712**		
LL 6	basal peat	8,4	0	7545	125	0,302	8,702	7545	-8,702	0	**-8,702**		

red — ***new U/Th date***
blue — ***older C14 date***
older U/Th date
unreliable rate

Accretion rates were calculated taking into account error ranges and error propagation[2].
Errors in ages are reported as 2 sigma.
Values were considered unreliable and discarded when error propagation exceeded reef accretion rate. New U/Th-data from this study.
Older ^{14}C-data and older U/Th-data from previous core studies[3-6].

Supplementary Table 3. Reliable reef accretion rates, rates of sea-level rise, and published temperature anomaly data[7, 8].
Rates of sea-level rise have been estimated in 250-year bins based on ANU_HR_71p230 sea-level data (see Supplementary Figure 2).

age (yr BP)	accretion rate (m/kyr)	branched accretion rate	massive accretion rate	mixed accretion rate	rate of SL-rise ANU_HR_71p230	SST anomaly Marcott 2013	SST anomaly Osman 2021
87	0,218	0,218			0,922	0,6	-0,074
2835	0,798			0,798	0,325	0,05	-0,167
5541	1,369	1,369			0,439	0,38	-0,213
5575	0,535	0,535			0,439	0,38	-0,213
6322	1,303		1,303		0,632	0,36	-0,157
3485	1,605	1,605			0,497	0,34	-0,204
436	0,922	0,922			0,17	-0,23	-0,074
870	0,425			0,425	0,149	-0,16	0,056
2517	0,665	0,665			0,273	0,04	-0,074
1084	6,542		6,542		0,43	-0,05	0,056
1464	0,36	0,36			0,079	-0,07	0,019
3825	2,098		2,098		0,787	0,18	-0,241
7479	2,306		2,306		5,199	0,38	-0,398
137	0,163			0,163	0,922	-0,35	-0,074
4424	4,949		4,949		0,722	0,27	-0,18
4277	4,933			4,933	0,722	0,25	-0,18
1990	0,572			0,572	0,011	-0,04	-0,083
5178	1,81		1,81		0,615	0,4	-0,13
2980	6,195		6,195		0,325	0,05	-0,167
3545	0,716			0,716	0,497	0,11	-0,204
1797	0,468		0,468		0,011	-0,06	-0,083
0	0,386	0,386			0,922	0,6	-0,074
4875	1,733	1,733			0,581	0,31	-0,13
6445	3,916		3,916		0,632	0,2	-0,157
6911	18,316			18,316	5,9	0,46	-0,296
7098	1,762		1,762		7,278	0,46	-0,296
122	0,451	0,451			0,922	0,6	-0,074
1452	0,352	0,352			0,079	-0,07	0,019
5005	0,567	0,567			0,615	0,37	-0,13
6988	0,9	0,9			5,9	0,46	-0,296
3684	0,893			0,893	0,497	0,18	-0,204
6679	2,851	2,851			0,43	0,41	-0,157
7705	14,62	14,62			3,789	0,37	-0,398
1968	1,006	1,006			0,011	-0,04	-0,083
5170	6,997	6,997			0,615	0,4	-0,13
5538	1,213	1,213			0,439	0,38	-0,213
6990	2,626	2,626			5,9	0,46	-0,296
7685	3,618	3,618			3,789	0,37	-0,398
990	0,346			0,346	0,149	-0,05	0,056
5247	3,378		3,378		0,569	0,4	-0,13
7066	9,386			9,386	7,278	0,46	-0,296
1105	0,396	0,396			0,097	-0,05	0,056
4435	4,501	4,501			0,722	0,27	-0,18
5146	1,883			1,883	0,615	0,4	-0,13
6259	17,081	17,081			0,632	0,36	-0,157
6420	3,085			3,085	0,632	0,34	-0,157
1035	0,437	0,437			0,097	-0,05	0,056
4695	4,868	4,868			0,631	0,31	-0,18
5188	2,492	2,492			0,615	0,4	-0,13
1670	1,034			1,034	0,087	-0,07	0,019
2975	2,025			2,025	0,325	0,05	-0,167
6252	23,684			23,684	0,632	0,36	-0,157
7320	6,736			6,736	5,199	0,4	-0,398
336	6,019	6,019			0,17	-0,35	-0,074
660	1,832			1,822	0,922	-0,16	-0,074
7070	3,222		3,222		7,278	0,46	-0,296
99	0,335	0,335			0,922	0,6	-0,074
5390	2,108	2,108			0,569	0,38	-0,213
6220	2,02			2,02	0,869	0,36	-0,176
7037	3,073	3,073			7,278	0,46	-0,296
5340	2,025			2,015	0,569	0,4	-0,213
6525	5,106	5,106			0,43	0,34	-0,157

**mean**	**3,359**	**2,884**	**3,162**	**4,255**
**SD**	**4,564**	**3,936**	**1,926**	**6,422**

Supplementary Table 4. Coral abundances per core location in three time bins (A-C: 3000-0, 3000-6000, 6000-9000 yrs BP) and in total (D).

A

	COMPETITIVE			STRESS-TOLERANT			WEEDY			
3000-0	***A. palmata***	***A. cervicornis***	***Millepora***	***Orbicella***	***Pseudodiploria***	***Siderastrea***	***Porites***	***Agaricia***	**Misc.**	**Total**
**BBR1**	10	2	0	2	1	0	4	0	2	21
**BBR2**	6	1	8	1	0	0	3	1	0	20
**BBR3**	2	0	0	2	0	0	0	0	1	5
**BBR4**	9	0	1	0	3	0	0	0	2	15
**BBR5**	7	0	0	0	2	0	0	0	1	10
**BBR6**	6	2	2	0	0	0	7	0	0	17
**BBR7**	6	0	0	0	2	0	0	0	0	8
**BBR8**	2	2	0	0	0	0	5	4	5	18
**BBR9**	5	0	1	0	2	0	0	0	2	10
**BBR10**	10	1	1	2	0	0	0	1	0	15
**BBR11**	4	0	0	0	0	0	0	0	0	4
**GR1**	5	2	0	0	0	0	1	0	4	12
**GR2**	0	0	0	0	0	0	0	0	0	0
**GR3**	6	3	0	0	0	0	1	0	0	10
**LR7**	0	0	2	0	0	0	0	6	1	9
**LR10**	1	0	0	0	0	2	0	0	4	7
**LR11**	0	0	0	1	0	0	0	0	0	1
**LR12**	3	2	0	6	3	0	0	1	1	16
**LR13**	2	0	0	0	0	0	0	0	0	2
**LR14**	0	0	0	5	0	0	1	0	1	7
**TR4**	4	0	6	3	1	0	0	3	0	17
**TR5**	1	0	0	11	2	0	0	1	1	16
**Sum (n)**	**89**	**15**	**21**	**33**	**16**	**2**	**22**	**17**	**25**	**240**
**%**	**37,1**	**6,3**	**8,8**	**13,8**	**6,7**	**0,8**	**9,2**	**7,1**	**10,4**	**100**
**sq root n (%)**	**3,9**	**1,6**	**1,9**	**2,4**	**1,7**	**0,6**	**2,0**	**1,7**	**2,1**	
**% Error**	**4,6**	**1,7**	**2,0**	**2,6**	**1,7**	**0,6**	**2,0**	**1,8**	**2,2**	

**Competitive**	**Stress-tolerant**	**Weedy**	**Misc.**	**Total**
12	3	4	2	21
15	1	4	0	20
2	2	0	1	5
10	3	0	2	15
7	2	0	1	10
10	0	7	0	17
6	2	0	0	8
4	0	9	5	18
6	2	0	2	10
12	2	1	0	15
4	0	0	0	4
7	0	1	4	12
0	0	0	0	0
9	0	1	0	10
2	0	6	1	9
1	2	0	4	7
0	1	0	0	1
5	9	1	1	16
2	0	0	0	2
0	5	1	1	7
10	4	3	0	17
1	13	1	1	16
**125**	**51**	**39**	**25**	**240**
**52,1**	**21,3**	**16,3**	**10,4**	**100**
**4,7**	**3,0**	**2,6**	**2,1**	
**5,8**	**3,3**	**2,8**	**2,2**	

B

6000-3000	***A. palmata***	***A. cervicornis***	***Millepora***	***Orbicella***	***Pseudodiploria***	***Siderastrea***	***Porites***	***Agaricia***	**Misc.**	**Total**
**BBR1**	21	1	0	2	0	0	1	0	0	25
**BBR2**	6	1	8	0	0	0	0	0	1	16
**BBR3**	8	0	0	7	0	0	2	0	0	17
**BBR4**	12	0	1	1	0	0	0	0	0	14
**BBR5**	0	1	0	6	0	0	0	0	0	7
**BBR6**	31	6	1	4	1	0	1	1	0	45
**BBR7**	25	4	10	2	0	0	1	8	0	50
**BBR8**	1	1	0	2	0	0	1	2	1	8
**BBR9**	1	0	0	0	0	1	0	0	0	2
**BBR10**	5	3	3	0	0	0	0	0	3	14
**BBR11**	8	0	3	0	1	0	2	0	1	15
**GR1**	7	6	0	6	2	0	2	1	6	30
**GR2**	1	0	0	3	0	0	0	0	0	4
**GR3**	2	1	0	0	0	0	0	0	0	3
**LR7**	4	0	0	9	4	0	4	7	1	29
**LR10**	0	1	0	21	0	2	0	1	2	27
**LR11**	0	0	0	11	0	0	4	1	3	19
**LR12**	0	0	0	4	0	0	2	0	0	6
**LR13**	0	0	0	21	0	0	0	0	0	21
**LR14**	0	0	0	11	0	0	0	0	0	11
**TR4**	5	0	2	0	1	0	0	2	0	10
**TR5**	0	0	0	0	0	0	0	0	0	0
**Sum (n)**	**137**	**25**	**28**	**110**	**9**	**3**	**20**	**23**	**18**	**373**
**%**	**36,7**	**6,7**	**7,5**	**29,5**	**2,4**	**0,8**	**5,4**	**6,2**	**4,8**	**100**
**sq root n (%)**	**3,1**	**1,3**	**1,4**	**2,8**	**0,8**	**0,5**	**1,2**	**1,3**	**1,1**	
**% Error**	**3,7**	**1,4**	**1,5**	**3,2**	**0,8**	**0,5**	**1,2**	**1,3**	**1,2**	

**Competitive**	**Stress-tolerant**	**Weedy**	**Misc.**	**Total**
22	2	1	0	25
15	0	0	1	16
8	7	2	0	17
13	1	0	0	14
1	6	0	0	7
38	5	2	0	45
39	2	9	0	50
2	2	3	1	8
1	1	0	0	2
11	0	0	3	14
11	1	2	1	15
13	8	3	6	30
1	3	0	0	4
3	0	0	0	3
4	13	11	1	29
1	23	1	2	27
0	11	5	3	19
0	4	2	0	6
0	21	0	0	21
0	11	0	0	11
7	1	2	0	10
0	0	0	0	0
**190**	**122**	**43**	**18**	**373**
**50,9**	**32,7**	**11,5**	**4,8**	**100**
**3,7**	**3,0**	**1,8**	**1,1**	
**4,5**	**3,4**	**1,9**	**1,2**	

C

9000-6000	***A. palmata***	***A. cervicornis***	***Millepora***	***Orbicella***	***Pseudodiploria***	***Siderastrea***	***Porites***	***Agaricia***	**Misc.**	**Total**
**BBR1**	7	5	7	25	0	4	3	8	5	64
**BBR2**	4	0	13	0	1	0	0	0	3	21
**BBR3**	13	13	0	4	3	0	0	0	0	33
**BBR4**	54	14	2	0	0	0	0	2	0	72
**BBR5**	2	0	0	3	0	0	0	0	0	5
**BBR6**	5	5	0	2	0	0	4	1	0	17
**BBR7**	0	2	0	11	0	0	0	1	0	14
**BBR8**	3	2	0	8	1	1	2	1	0	18
**BBR9**	0	0	0	19	0	0	4	0	0	23
**BBR10**	20	2	1	4	3	1	1	0	2	34
**BBR11**	16	2	8	1	2	0	4	0	3	36
**GR1**	33	13	0	9	1	0	1	0	6	63
**GR2**	4	6	0	24	5	0	6	1	0	46
**GR3**	0	0	0	0	0	0	0	0	0	0
**LR7**	0	1	3	0	10	0	0	2	0	16
**LR10**	0	0	0	0	0	0	0	0	0	0
**LR11**	0	0	0	0	0	0	0	0	0	0
**LR12**	0	0	2	4	3	2	1	0	0	12
**LR13**	0	0	0	0	0	0	0	0	0	0
**LR14**	0	0	0	23	0	0	0	0	0	23
**TR4**	0	0	0	0	0	0	0	0	0	0
**TR5**	0	0	0	0	0	0	0	0	0	0
**Sum (n)**	**161**	**65**	**36**	**137**	**29**	**8**	**26**	**16**	**19**	**497**
**%**	**32,4**	**13,1**	**7,2**	**27,6**	**5,8**	**1,6**	**5,2**	**3,2**	**3,8**	**100**
**sq root n (%)**	**2,6**	**1,6**	**1,2**	**2,4**	**1,1**	**0,6**	**1,0**	**0,8**	**0,9**	
**% Error**	**2,9**	**1,7**	**1,3**	**2,7**	**1,1**	**0,6**	**1,1**	**0,8**	**0,9**	

**Competitive**	**Stress-tolerant**	**Weedy**	**Misc.**	**Total**
19	29	11	5	64
17	1	0	3	21
26	7	0	0	33
70	0	2	0	72
2	3	0	0	5
10	2	5	0	17
2	11	1	0	14
5	10	3	0	18
0	19	4	0	23
23	8	1	2	34
26	3	4	3	36
46	10	1	6	63
10	29	7	0	46
0	0	0	0	0
4	10	2	0	16
0	0	0	0	0
0	0	0	0	0
2	9	1	0	12
0	0	0	0	0
0	23	0	0	23
0	0	0	0	0
0	0	0	0	0
**262**	**174**	**42**	**19**	**497**
**52,7**	**35**	**8,5**	**3,8**	**100**
**3,3**	**2,7**	**1,3**	**0,9**	
**4,0**	**3,1**	**1,4**	**0,9**	

D

ALL	***A. palmata***	***A. cervicornis***	***Millepora***	***Orbicella***	***Pseudodiploria***	***Siderastrea***	***Porites***	***Agaricia***	**Misc.**	**Total**
**BBR1**	38	8	7	29	1	4	8	8	7	110
**BBR2**	16	2	29	1	1	0	3	1	4	57
**BBR3**	23	13	0	13	3	0	2	0	1	55
**BBR4**	75	14	4	1	3	0	0	2	2	101
**BBR5**	9	1	0	9	2	0	0	0	1	22
**BBR6**	42	13	3	6	1	0	12	2	0	79
**BBR7**	31	6	10	13	2	0	1	9	0	72
**BBR8**	6	5	0	10	1	1	8	7	6	44
**BBR9**	6	0	1	19	2	1	4	0	2	35
**BBR10**	35	6	5	6	3	1	1	1	5	63
**BBR11**	28	2	11	1	3	0	6	0	4	55
**GR1**	45	21	0	15	3	0	4	1	16	105
**GR2**	5	6	0	27	5	0	6	1	0	50
**GR3**	8	4	0	0	0	0	1	0	0	13
**LR7**	4	1	5	9	14	0	4	15	2	54
**LR10**	1	1	0	21	0	4	0	1	6	34
**LR11**	0	0	0	12	0	0	4	1	3	20
**LR12**	3	2	2	14	6	2	3	1	1	34
**LR13**	2	0	0	21	0	0	0	0	0	23
**LR14**	0	0	0	39	0	0	1	0	1	41
**TR4**	9	0	8	3	2	0	0	5	0	27
**TR5**	1	0	0	11	2	0	0	1	1	16
**Sum (n)**	**387**	**105**	**85**	**280**	**54**	**13**	**68**	**56**	**62**	**1110**
**%**	**34,9**	**9,5**	**7,7**	**25,2**	**4,9**	**1,2**	**6,1**	**5**	**5,6**	**100**
**sq root n (%)**	**1,8**	**0,9**	**0,8**	**1,5**	**0,7**	**0,3**	**0,7**	**0,7**	**0,7**	
**% Error**	**2,1**	**1,0**	**0,9**	**1,7**	**0,7**	**0,3**	**0,8**	**0,7**	**0,7**	

**Competitive**	**Stress-tolerant**	**Weedy**	**Misc.**	**Total**
53	34	16	7	110
47	2	4	4	57
36	16	2	1	55
93	4	2	2	101
10	11	0	1	22
58	7	14	0	79
47	15	10	0	72
11	12	15	6	44
7	22	4	2	35
46	10	2	5	63
41	4	6	4	55
66	18	5	16	105
11	32	7	0	50
12	0	1	0	13
10	23	19	2	54
2	25	1	6	34
0	12	5	3	20
7	22	4	1	34
2	21	0	0	23
0	39	1	1	41
17	5	5	0	27
1	13	1	1	16
**577**	**347**	**124**	**62**	**1110**
**52**	**31,3**	**11,2**	**5,6**	**100**
**2,2**	**1,7**	**1,0**	**0,7**	
**2,7**	**1,9**	**1,1**	**0,7**	

Supplementary Table 5. Latitudes and longitudes of 22 rotary core holes and 14 vibracores from which basal peat data were used.

core	LAT		LONG	
	degrees N	min	degrees W	min
**Reef cores**				
GR 1	16	44,6960	87	48,1000
GR 2	16	47,1860	87	51,7380
GR 3	16	47,1020	87	49,8780
TR 4	17	19,6660	87	47,2211
TR 5	17	32,6004	87	47,8398
LR 7	17	13,5057	87	31,7226
LR 10	17	22,6300	87	34,5600
LR 11	17	21,9500	87	31,7700
LR 12	17	28,2300	87	29,2300
LR 13	17	22,4000	87	32,0000
LR 14	17	20,3800	87	31,3900
BBR 1	16	54,5900	88	4,8000
BBR 2	17	4,1600	87	59,5700
BBR 3	17	13,5700	88	2,8600
BBR 4	17	28,7600	88	2,5200
BBR 5	16	29,1000	88	0,1200
BBR 6	16	41,7300	88	4,4900
BBR 7	16	41,7300	88	4,4900
BBR 8	16	6,8800	88	15,7000
BBR 9	16	17,9800	88	9,3000
BBR 10	16	58,7400	88	1,7100
BBR 11	16	58,7400	88	1,7100
**Cores with basal peat**				
TR 8	17	10,2088	87	53,8684
TR 9	17	10,2088	87	53,8684
LR 10	17	22,6300	87	34,5600
LR 13	17	22,4000	87	32,0000
LR 14	17	20,3800	87	31,3900
GL 5	16	50,4000	87	45,1800
GL 7	16	53,2500	87	46,3400
GL 9	16	44,4100	87	50,4100
TL 4	17	14,5500	87	56,7000
TL 6	17	22,7000	87	55,3000
TL 7	17	22,3500	87	52,4800
TL 12	17	28,3700	87	50,9000
LL 5	17	23,1100	87	32,1000
LL 6	17	9,3900	87	36,4500

Supplementary Table 6. The data-model misfit parameter for the ice-Earth model parameter sets considered in this study. Note that, other than for the Earth model VM5a[9], the Earth viscosity parameters are indicated by a triplet of numbers that give the lithosphere thickness (km), upper mantle viscosity (×10^{21} Pas), and lower mantle viscosity (×10^{21} Pas).

**Earth-ice model**	ANU_71-0.2-30	ICE6G_120-5-50	ANU_120-5-50	ICE6G_VM5a
**Misfit parameter (δ)**	0.1419598	0.1428470	0.1573298	0.2267989

We considered four different model parameter sets comprising two ice history reconstructions: the model developed by colleagues at the Australia National University (henceforth referred to as the 'ANU' model[10]) and the ICE-6G model[9]. We partnered these ice models with their optimal Earth viscosity models: VM5a for ICE-6G[9] and a model defined by lithosphere thickness of 71 km, an upper mantle viscosity of 2×10^{20} Pas, and lower mantle viscosity of 3×10^{22} Pas[10]. We also considered an Earth model found to give optimal fits to a circum-Caribbean RSL data set: lithosphere thickness of 120 km, upper mantle viscosity of 5×10^{21} Pas, and lower mantle viscosity of 5×10^{22} Pas[11]. In order to determine which of these four parameter sets is most compatible with the observations, we quantify the data-model misfit using the coral samples and the equation[12].

$$\delta = \frac{1}{N}\sqrt{\sum_{i=1}^{N}\left(\frac{RSL_i^{data} - RSL_i^{model}}{\Delta_{rsl,i}}\right)^2 + \left(\frac{t_i^{data} - t_i^{model}}{\Delta_{t,i}}\right)^2}$$

Where RSL_i^{data} and RSL_i^{model} are the observed and predicted RSL elevation for the ith coral sample; t_i^{data} and t_i^{model} are the observed and predicted RSL age for the ith coral sample; $\Delta_{t,i}$ and $\Delta_{rsl,i}$ are 1σ age and elevation uncertainties of the ith coral sample; and N is the total number of coral samples. It should be noted that the RSL elevation uncertainty includes the uncertainty associated with drilling and estimating the depth of the coral sample in the core as well as the living depth range (5 m) to define a plausible range for RSL. The model RSL curve is calculated at each core location and the closest point on this curve to the reconstructed RSL value is estimated through the norm-2 distance or Euclidean approach[12]. The results indicate that the ANU ice model with the Earth model defined by a 71 km thick lithosphere, upper mantle viscosity of 2×10^{20} Pas, and lower mantle viscosity of 3×10^{22} Pas provides the minimum misfit (optimal fit) among the considered model parameters.

Supplementary Figure 1. Age-depth cross-plot of complete sea-level data of Belize. Data have been corrected for minimal subsidence, i.e., 0.04 - 0.11 m/kyr, based on age data and elevation of Pleistocene corals[13].

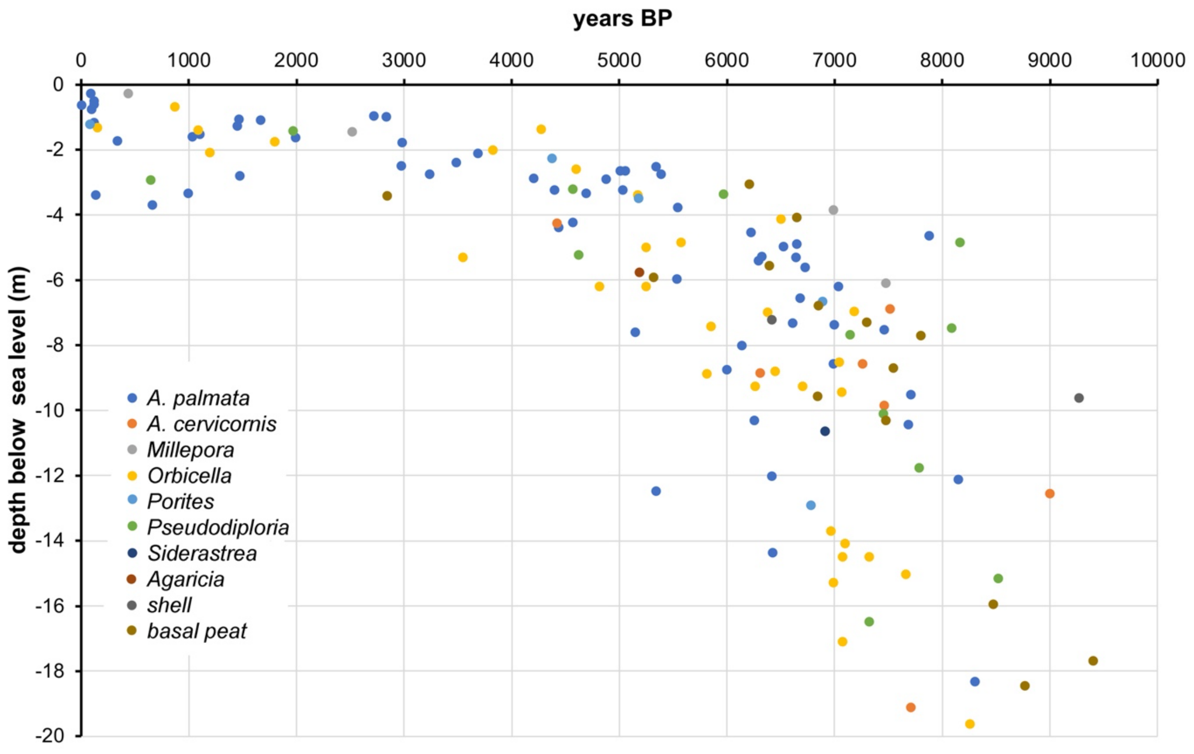

Supplementary Figure 2. The modelled relative sea level (RSL) curve for each ice-Earth model parameter set (see suppl.-table 6). The blue line (ANU_71p230) shows the optimal modelled RSL estimated from the data-model misfit analysis on *Acropora palmata* coral samples[14]. RSL curves are plotted at the average position of all samples within each site (see the white diamonds in Supplementary Figure 3). The horizontal bars show ±1σ age uncertainty and the vertical bars of coral samples indicate the reconstructed RSL based on a living depth range of 0-5 m below low tide level.

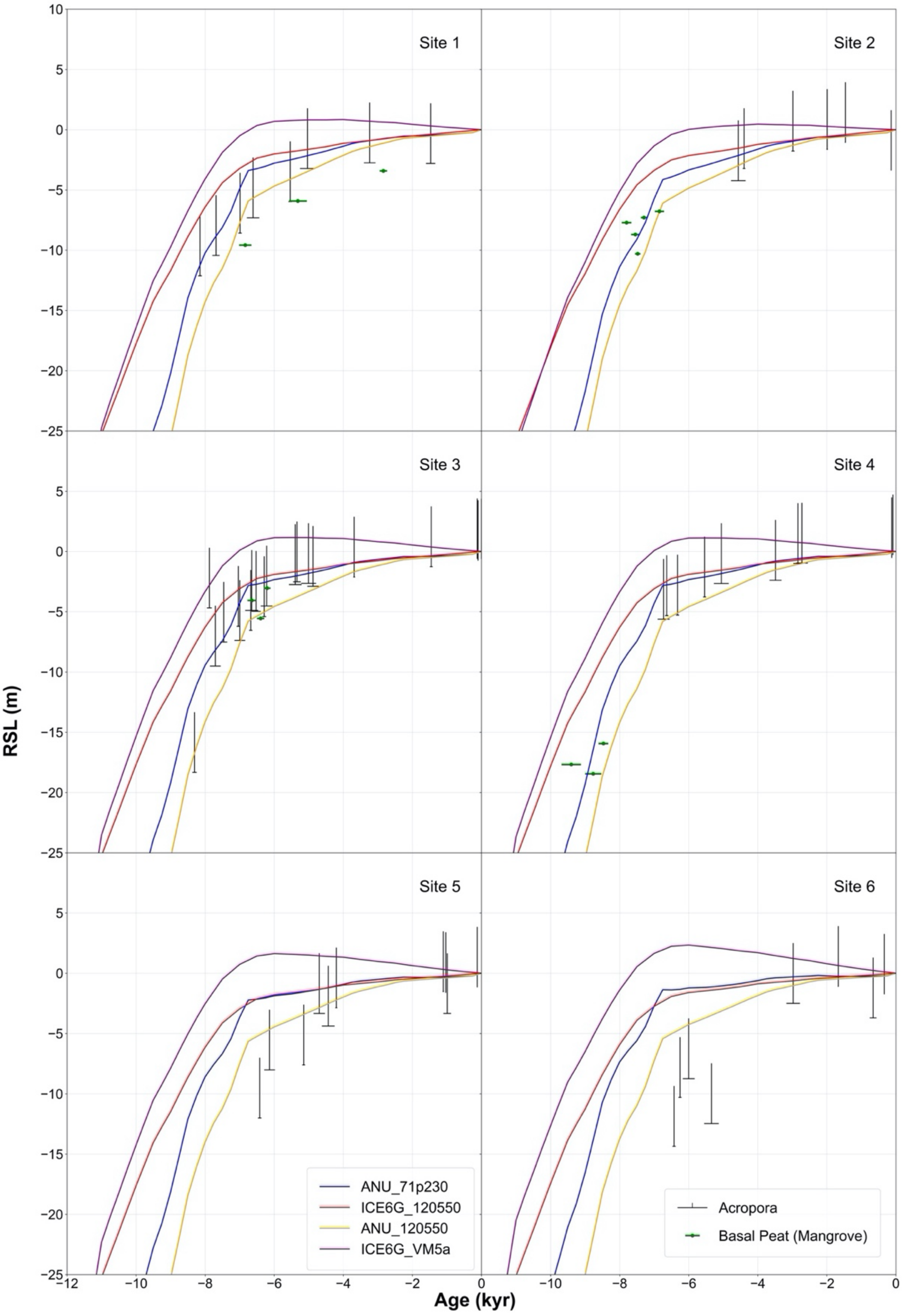

Supplementary Figure 3. Map showing the spatial variability of RSL at 6 kyr BP estimated from the best-fitting model as well as spatial distribution of coral samples over 6 sites in this study. The locations of *Acropora* and basal peat samples are indicated by red and blue circles, respectively. The white diamond in each site gives the average position of all coral samples; these points are the locations at which data-model curves are generated for visual comparison between model curves and observations (see Supplementary Figure 2). The map was drawn using the GMT (version 6) software[15].

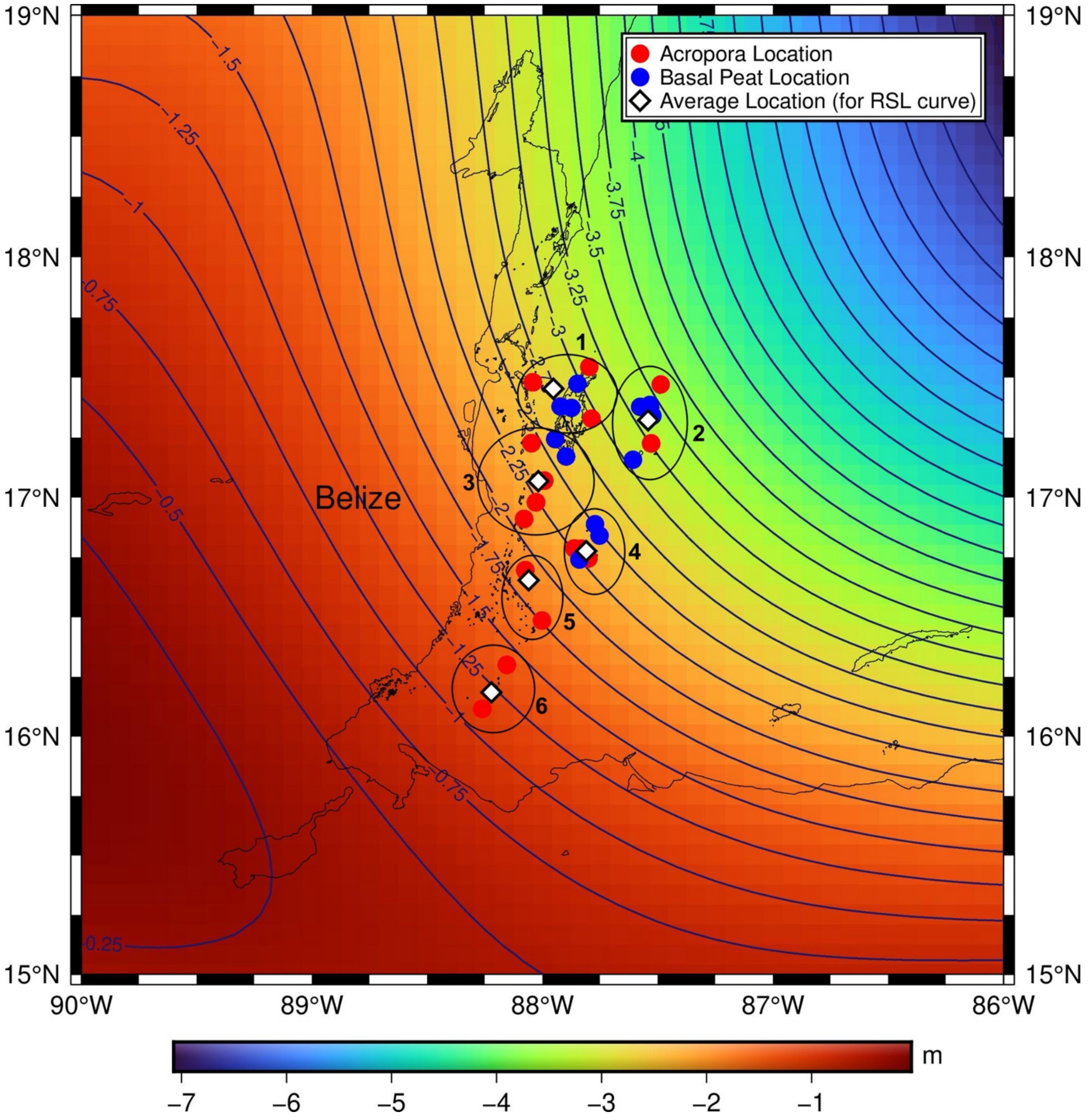

Supplementary Figure 4. Detailed descriptions of coral occurrence in the 22 drill cores from Belize barrier and atoll reefs. Abbreviations of dated corals include Ac=*Acropora cervicornis*, Ap=*Acropora palmata*, M=*Millepora* sp., O=*Orbicella* sp., P=*Porites* sp., Ps=*Pseudodiploria* sp., s=mollusk shell.

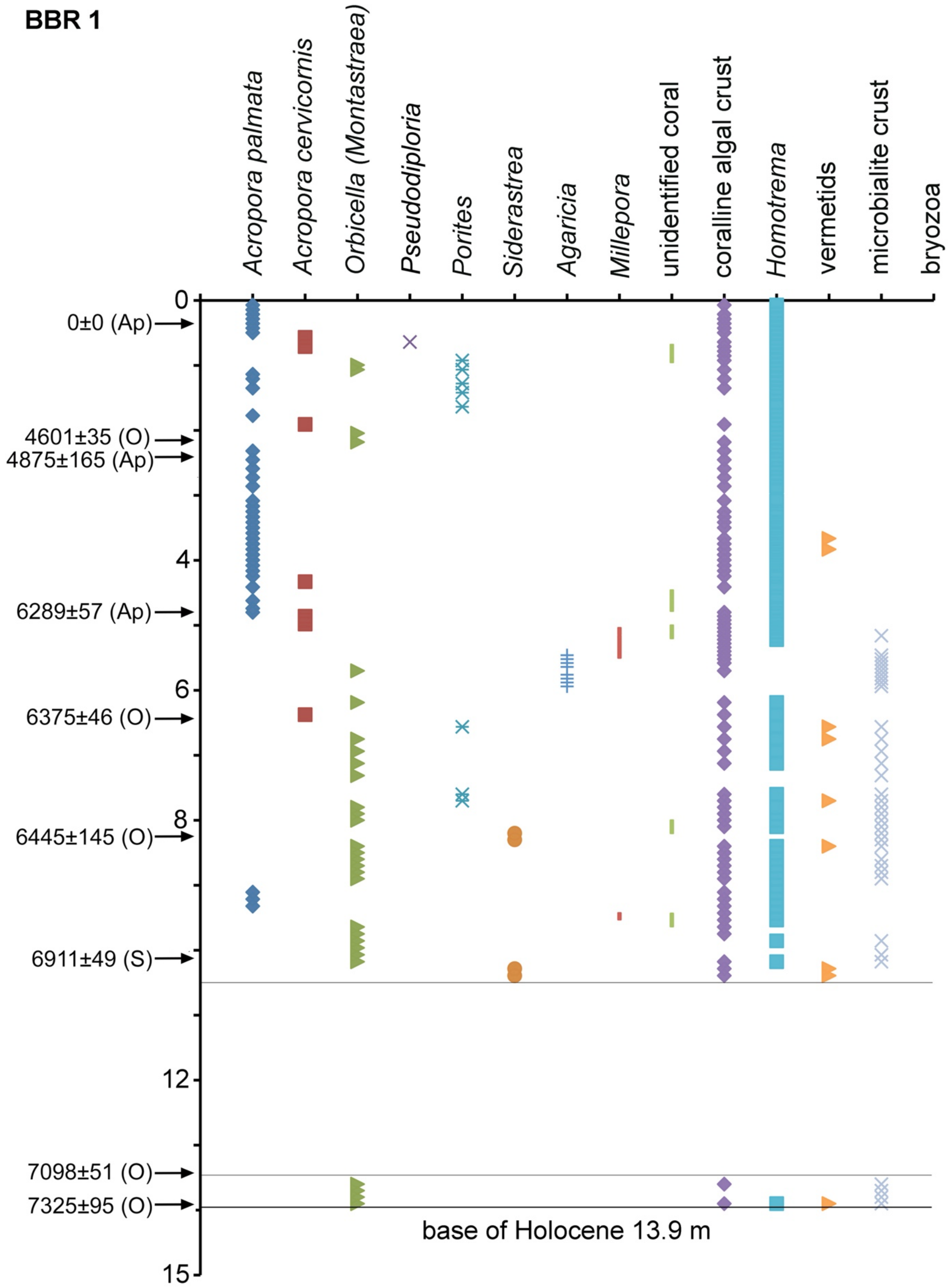

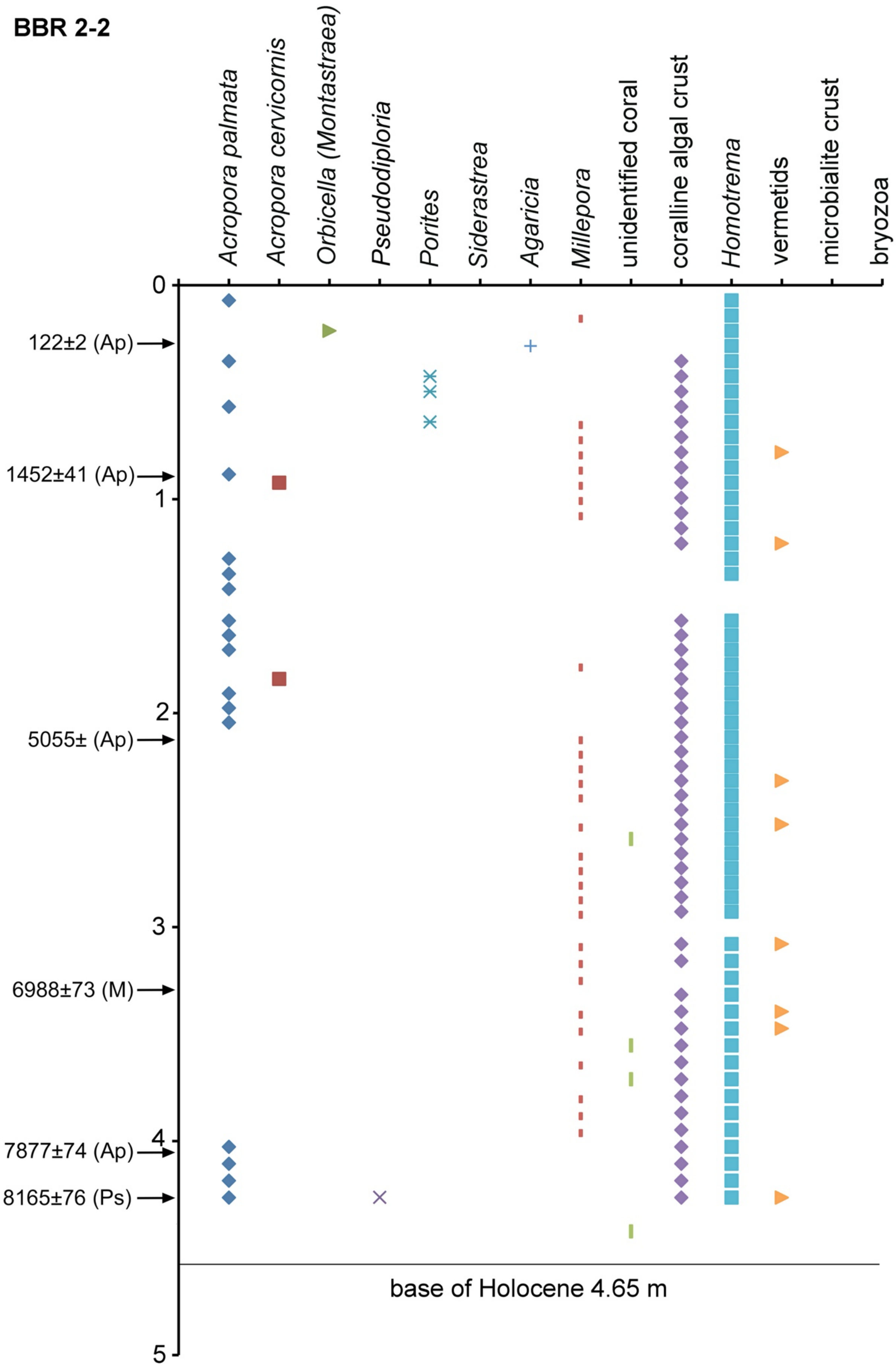
BBR 2-2
Acropora palmata
Acropora cervicornis
Orbicella (Montastraea)
Pseudodiploria
Porites
Siderastrea
Agaricia
Millepora
unidentified coral
coralline algal crust
Homotrema
vermetids
microbialite crust
bryozoa
0
1
2
3
4
5
122±2 (Ap)
1452±41 (Ap)
5055± (Ap)
6988±73 (M)
7877±74 (Ap)
8165±76 (Ps)
base of Holocene 4.65 m

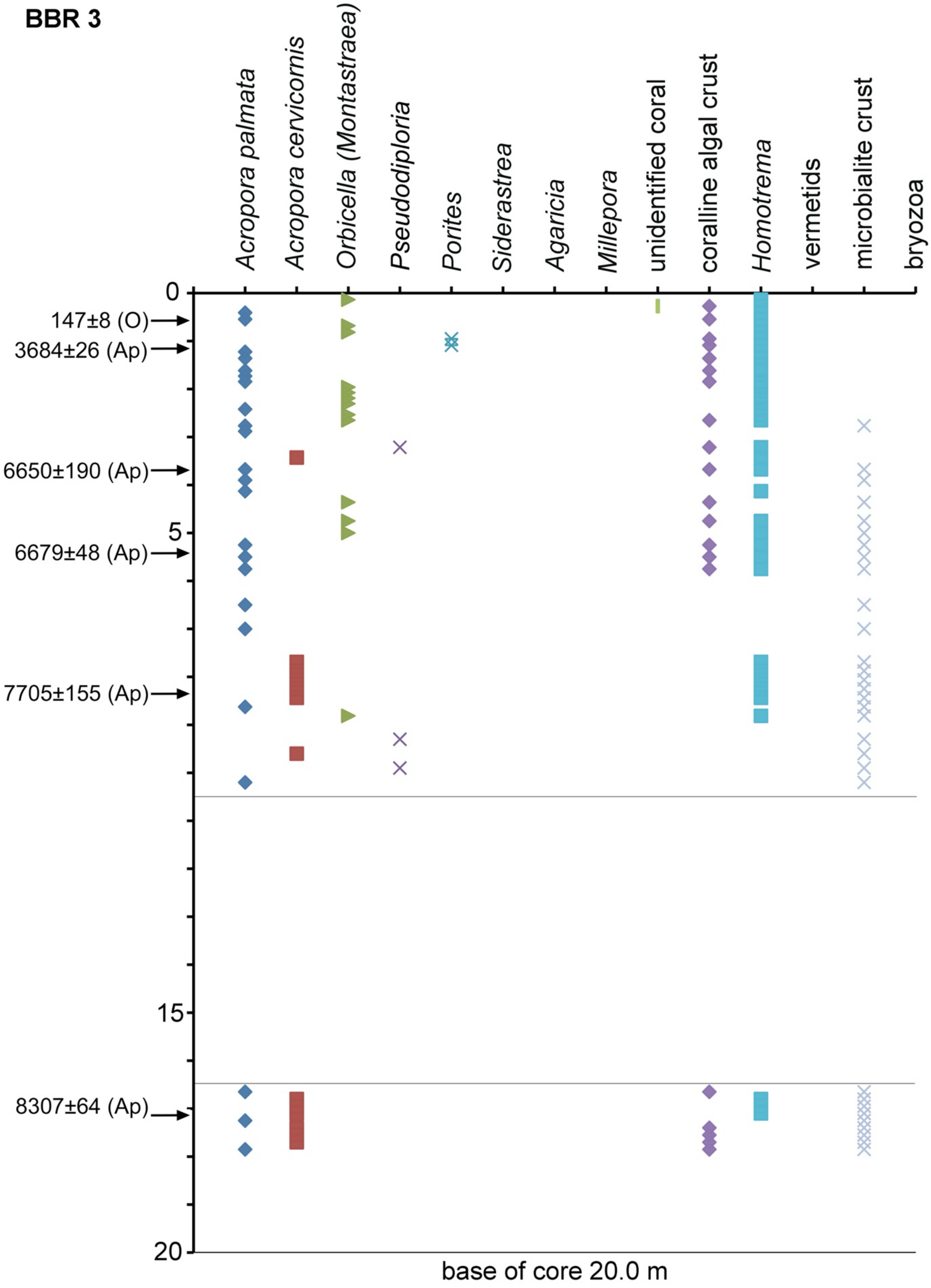
BBR 3
Acropora palmata
Acropora cervicornis
Orbicella (Montastraea)
Pseudodiploria
Porites
Siderastrea
Agaricia
Millepora
unidentified coral
coralline algal crust
Homotrema
vermetids
microbialite crust
bryozoa
0
5
15
20
147±8 (O)
3684±26 (Ap)
6650±190 (Ap)
6679±48 (Ap)
7705±155 (Ap)
8307±64 (Ap)
base of core 20.0 m

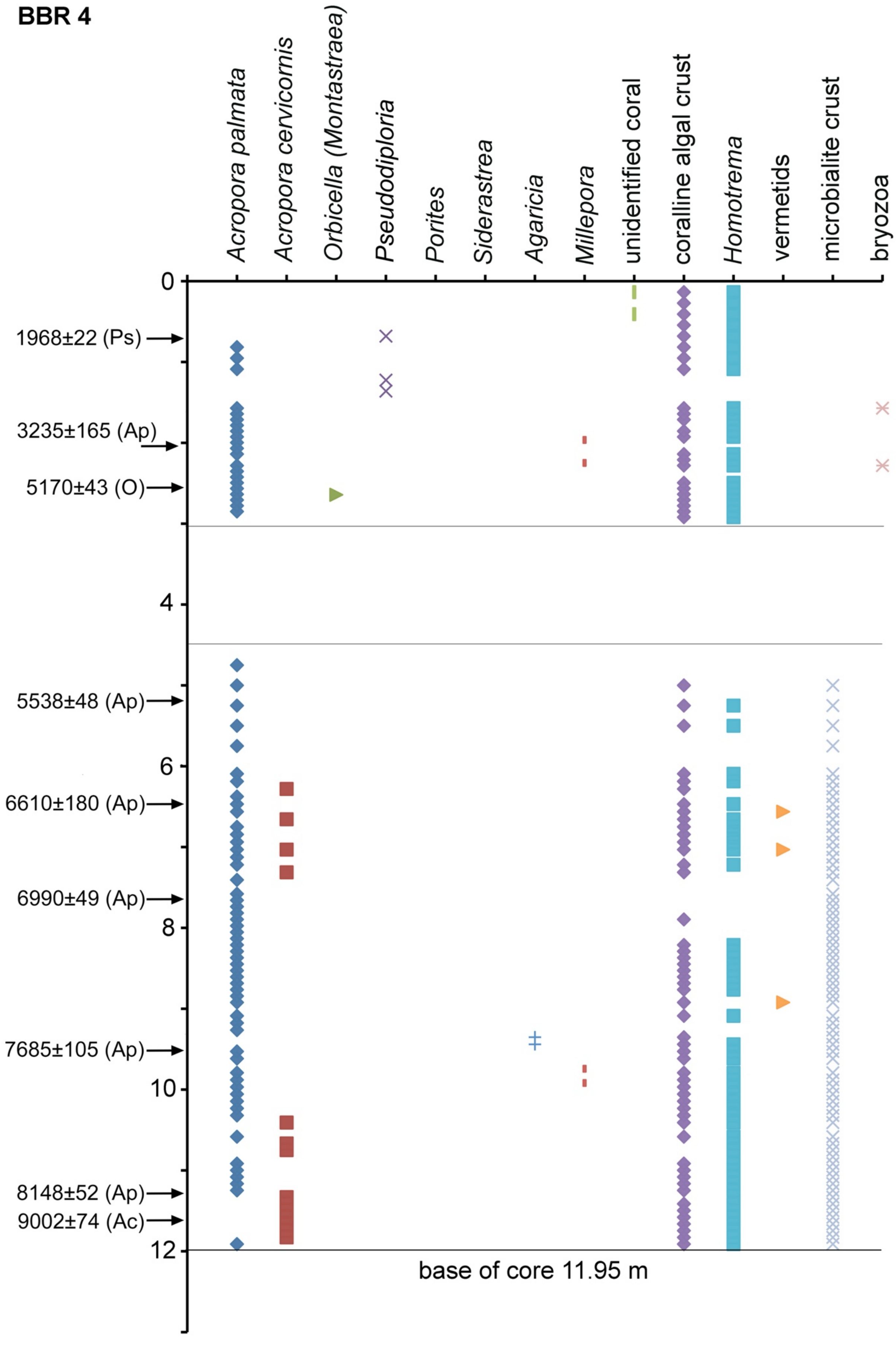

BBR 4
Acropora palmata
Acropora cervicornis
Orbicella (Montastraea)
Pseudodiploria
Porites
Siderastrea
Agaricia
Millepora
unidentified coral
coralline algal crust
Homotrema
vermetids
microbialite crust
bryozoa
0
4
6
8
10
12
1968±22 (Ps)
3235±165 (Ap)
5170±43 (O)
5538±48 (Ap)
6610±180 (Ap)
6990±49 (Ap)
7685±105 (Ap)
8148±52 (Ap)
9002±74 (Ac)
base of core 11.95 m

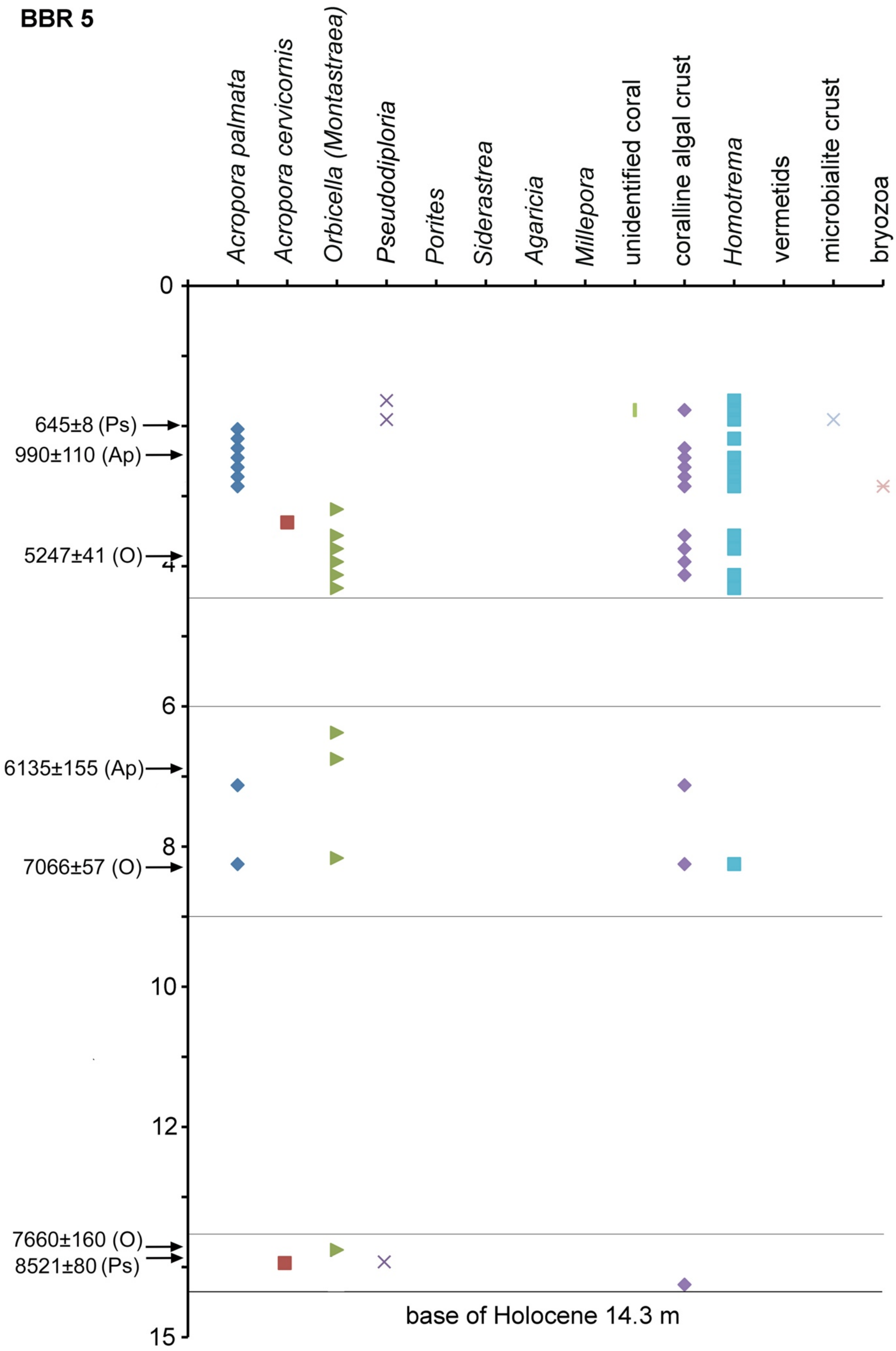
BBR 5
Acropora palmata
Acropora cervicornis
Orbicella (Montastraea)
Pseudodiploria
Porites
Siderastrea
Agaricia
Millepora
unidentified coral
coralline algal crust
Homotrema
vermetids
microbialite crust
bryozoa
0
4
6
8
10
12
15
645±8 (Ps)
990±110 (Ap)
5247±41 (O)
6135±155 (Ap)
7066±57 (O)
7660±160 (O)
8521±80 (Ps)
base of Holocene 14.3 m

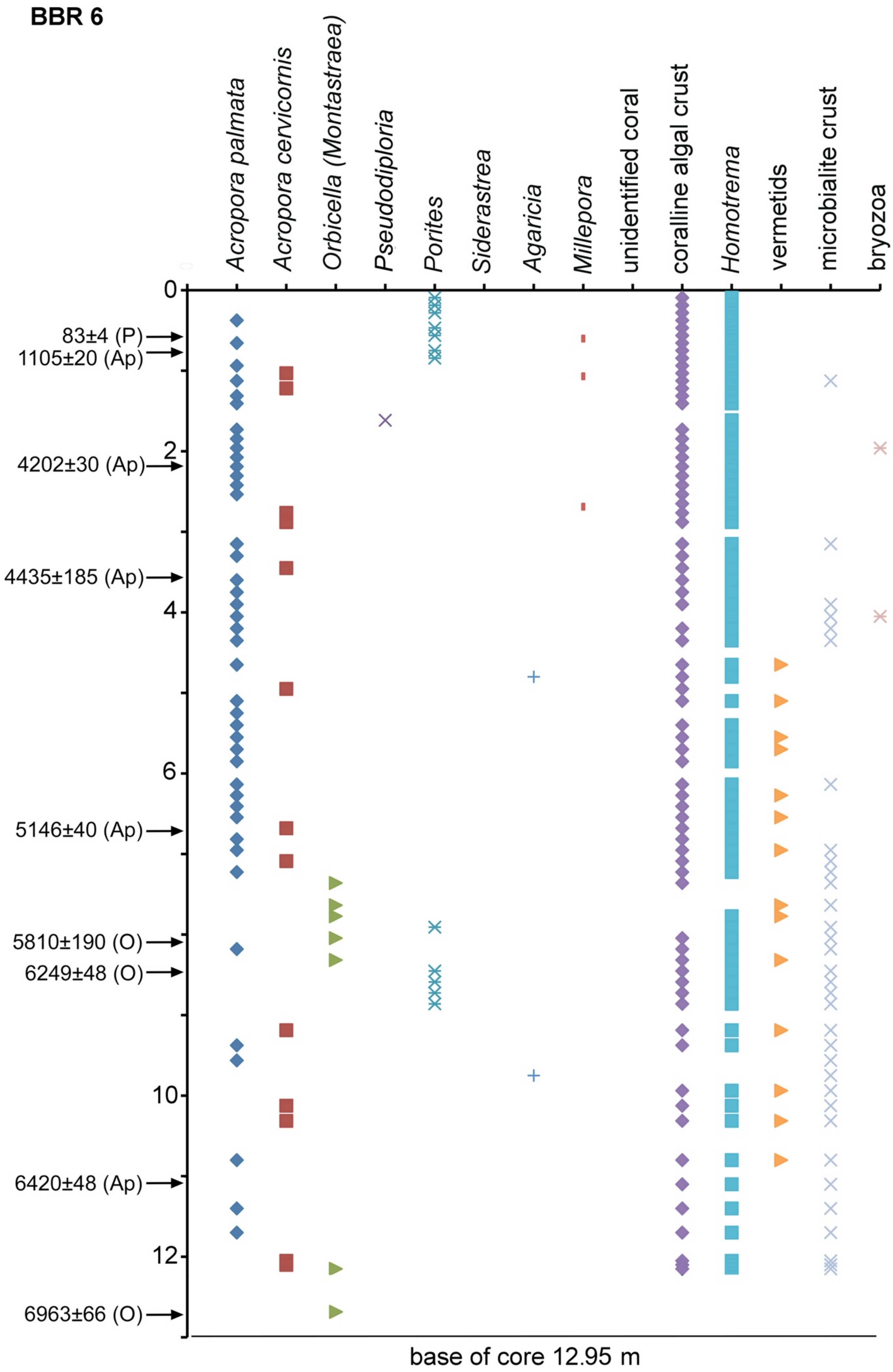

BBR 6
Acropora palmata
Acropora cervicornis
Orbicella (Montastraea)
Pseudodiploria
Porites
Siderastrea
Agaricia
Millepora
unidentified coral
coralline algal crust
Homotrema
vermetids
microbialite crust
bryozoa
0
2
4
6
10
12
83±4 (P)
1105±20 (Ap)
4202±30 (Ap)
4435±185 (Ap)
5146±40 (Ap)
5810±190 (O)
6249±48 (O)
6420±48 (Ap)
6963±66 (O)
base of core 12.95 m

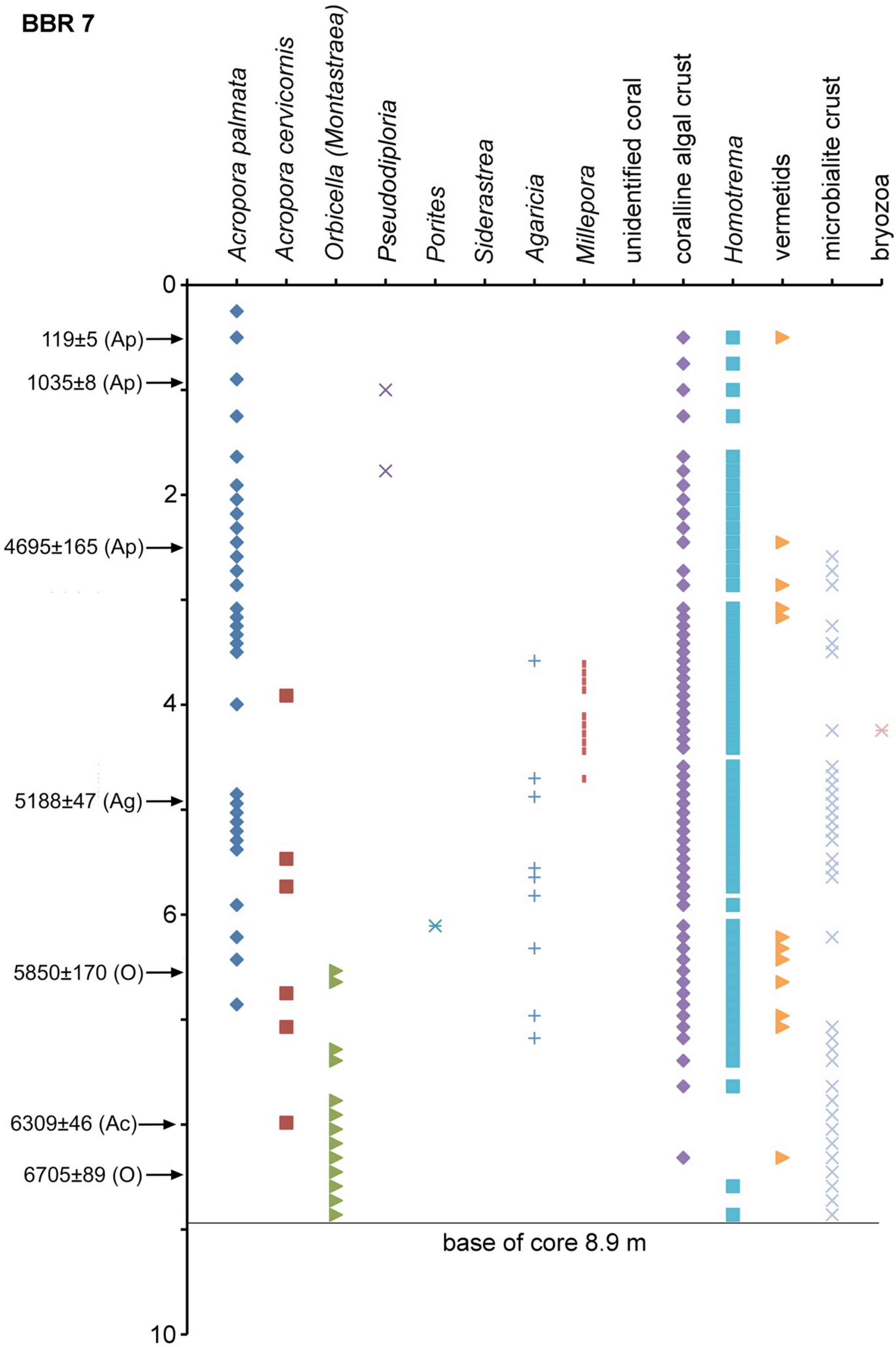

BBR 7
Acropora palmata
Acropora cervicornis
Orbicella (Montastraea)
Pseudodiploria
Porites
Siderastrea
Agaricia
Millepora
unidentified coral
coralline algal crust
Homotrema
vermetids
microbialite crust
bryozoa
0
2
4
6
10
119±5 (Ap)
1035±8 (Ap)
4695±165 (Ap)
5188±47 (Ag)
5850±170 (O)
6309±46 (Ac)
6705±89 (O)
base of core 8.9 m

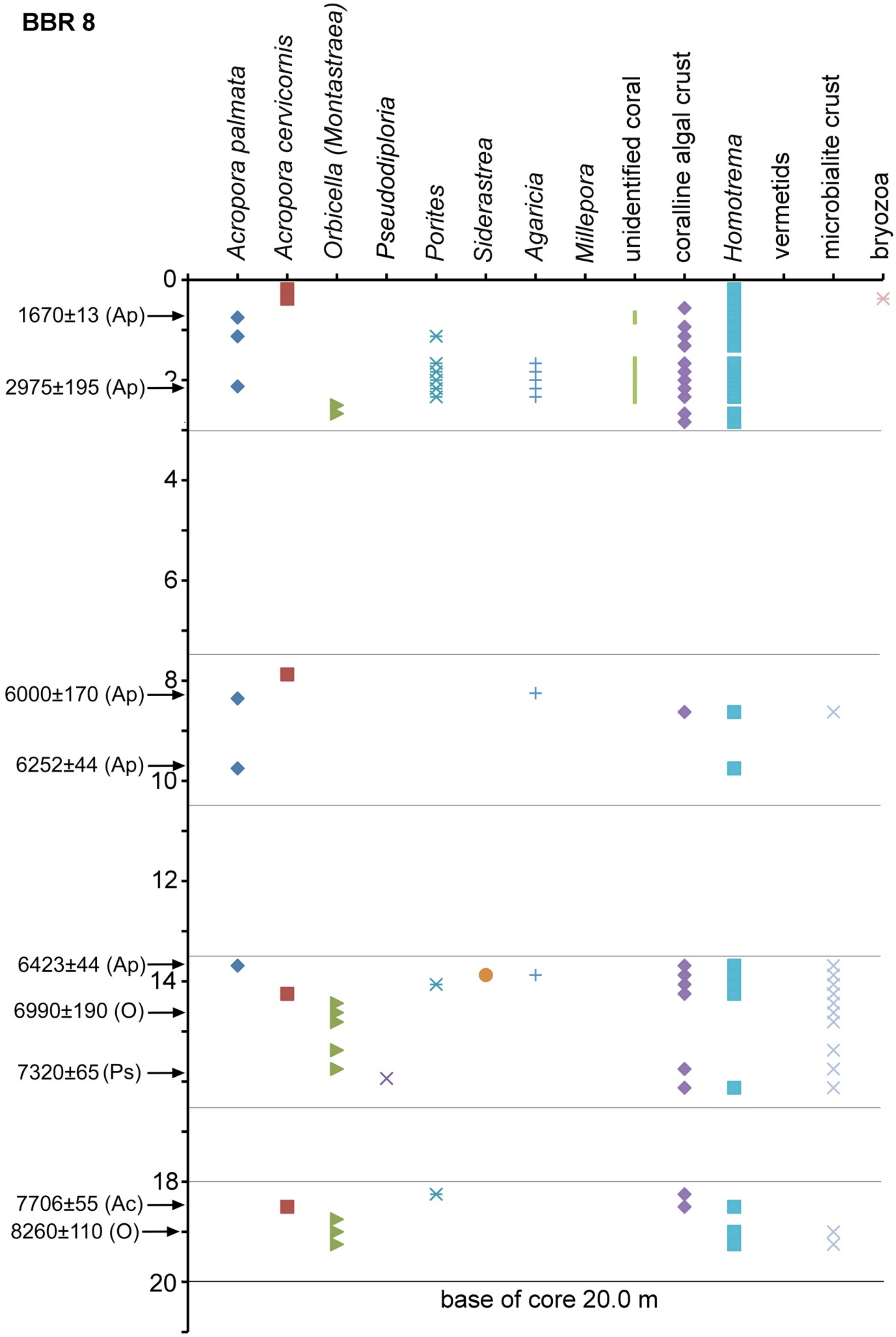
BBR 8
Acropora palmata
Acropora cervicornis
Orbicella (Montastraea)
Pseudodiploria
Porites
Siderastrea
Agaricia
Millepora
unidentified coral
coralline algal crust
Homotrema
vermetids
microbialite crust
bryozoa
1670±13 (Ap)
2975±195 (Ap)
6000±170 (Ap)
6252±44 (Ap)
6423±44 (Ap)
6990±190 (O)
7320±65 (Ps)
7706±55 (Ac)
8260±110 (O)
0
2
4
6
8
10
12
14
18
20
base of core 20.0 m

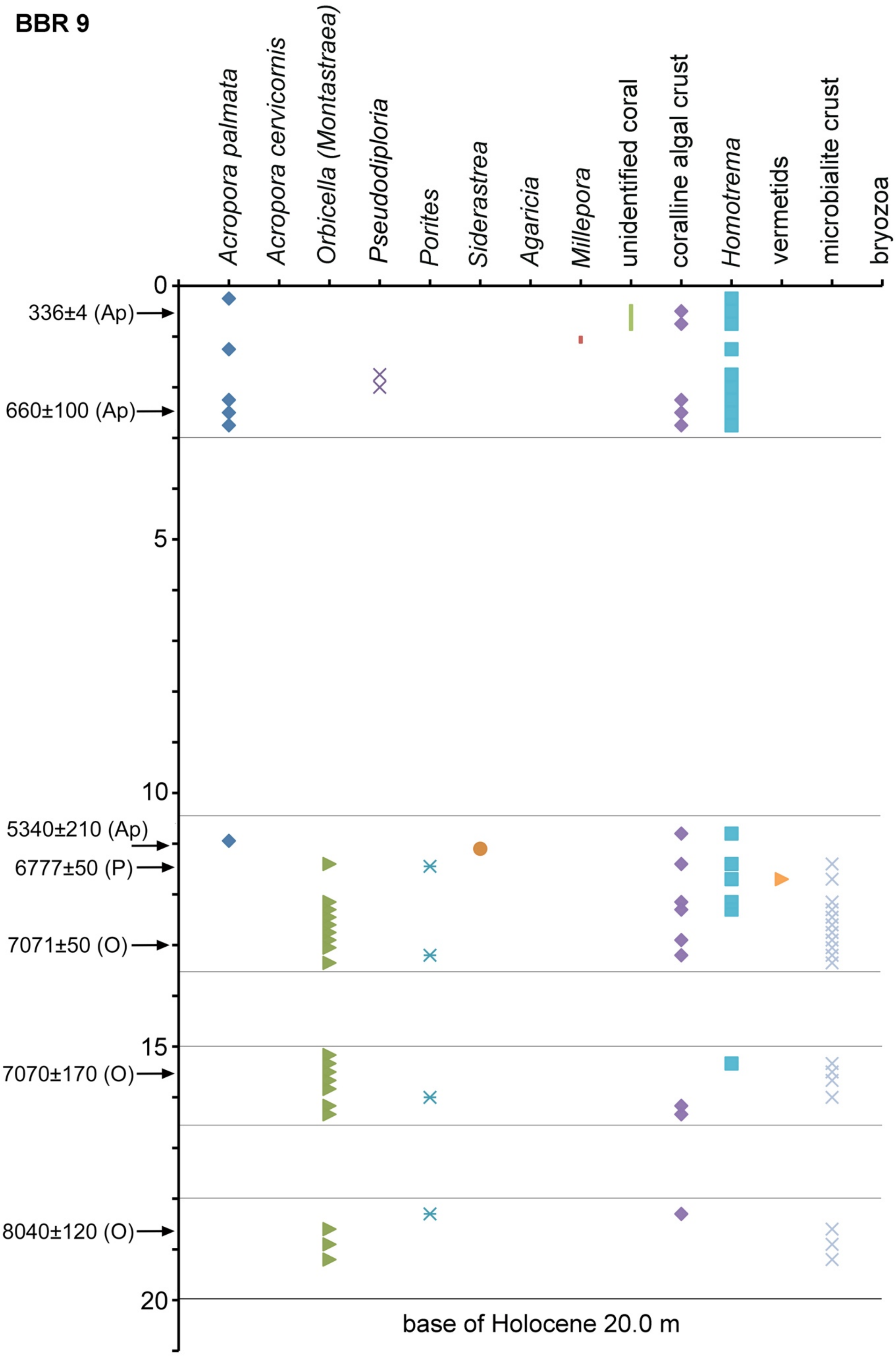
BBR 9
Acropora palmata
Acropora cervicornis
Orbicella (Montastraea)
Pseudodiploria
Porites
Siderastrea
Agaricia
Millepora
unidentified coral
coralline algal crust
Homotrema
vermetids
microbialite crust
bryozoa
0
5
10
15
20
336±4 (Ap)
660±100 (Ap)
5340±210 (Ap)
6777±50 (P)
7071±50 (O)
7070±170 (O)
8040±120 (O)
base of Holocene 20.0 m

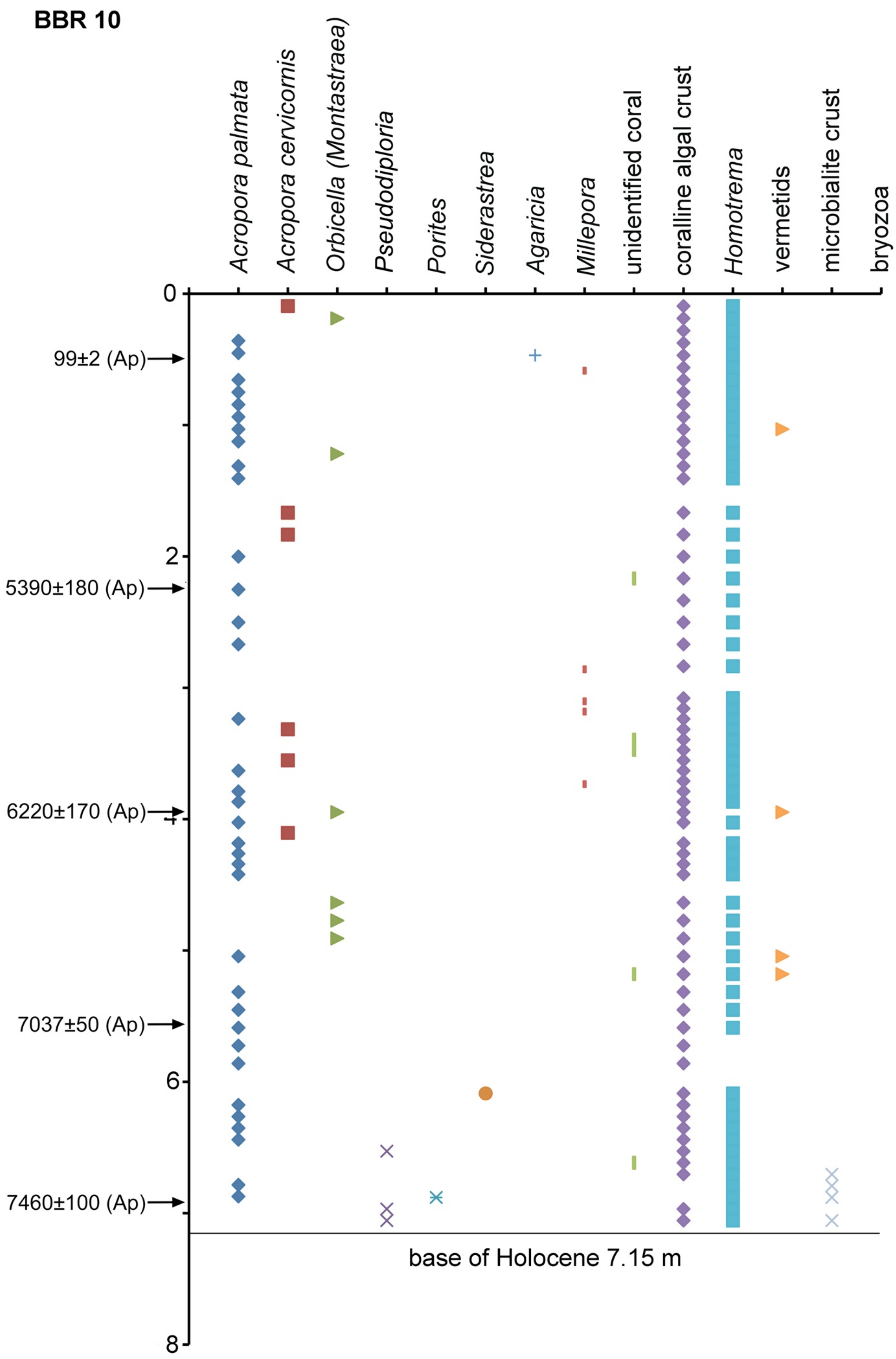
BBR 10
Acropora palmata
Acropora cervicornis
Orbicella (Montastraea)
Pseudodiploria
Porites
Siderastrea
Agaricia
Millepora
unidentified coral
coralline algal crust
Homotrema
vermetids
microbialite crust
bryozoa
0
2
6
8
99±2 (Ap)
5390±180 (Ap)
6220±170 (Ap)
7037±50 (Ap)
7460±100 (Ap)
base of Holocene 7.15 m

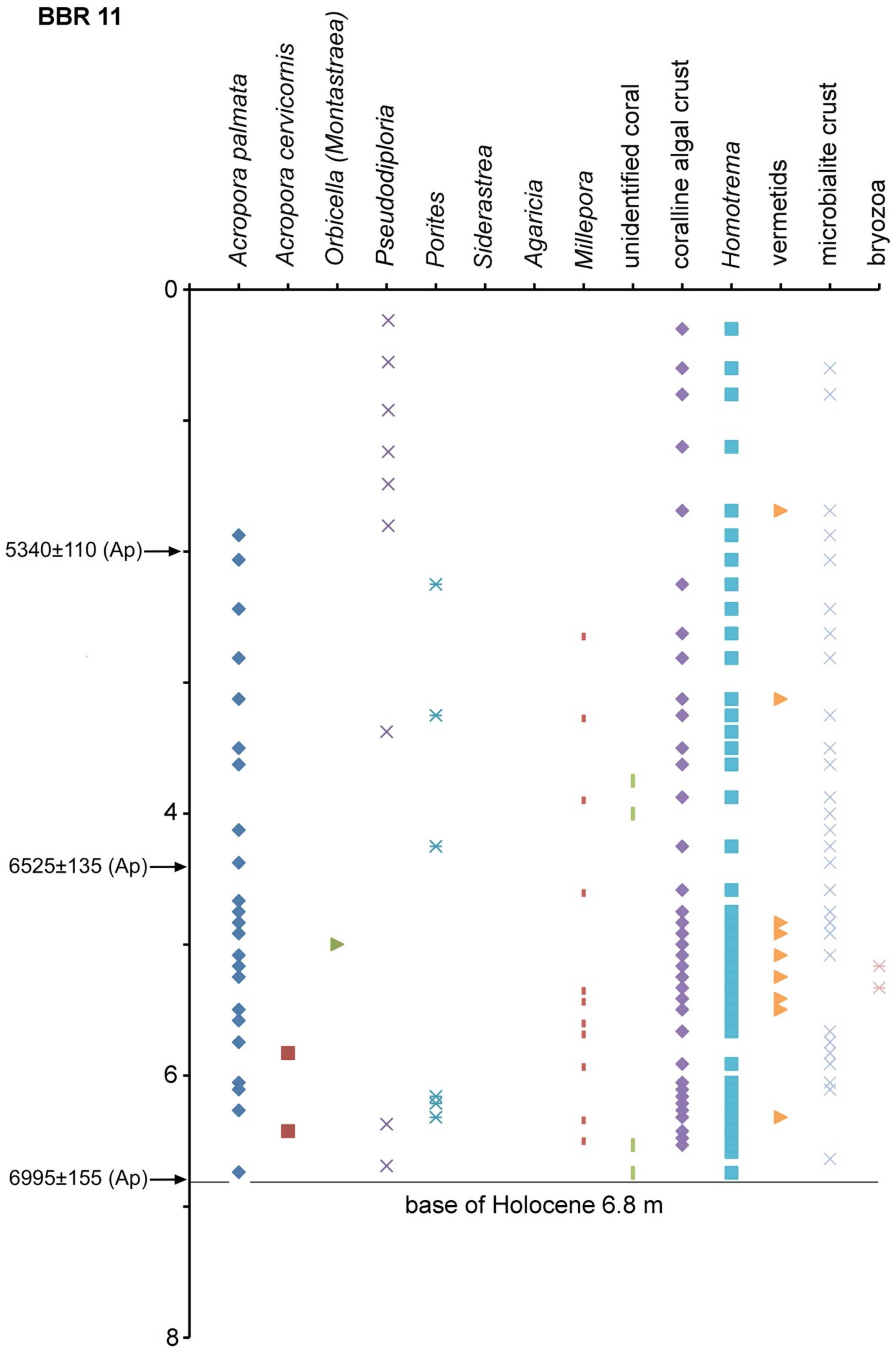
BBR 11
Acropora palmata
Acropora cervicornis
Orbicella (Montastraea)
Pseudodiploria
Porites
Siderastrea
Agaricia
Millepora
unidentified coral
coralline algal crust
Homotrema
vermetids
microbialite crust
bryozoa
0
4
6
8
5340±110 (Ap)
6525±135 (Ap)
6995±155 (Ap)
base of Holocene 6.8 m

GR 1

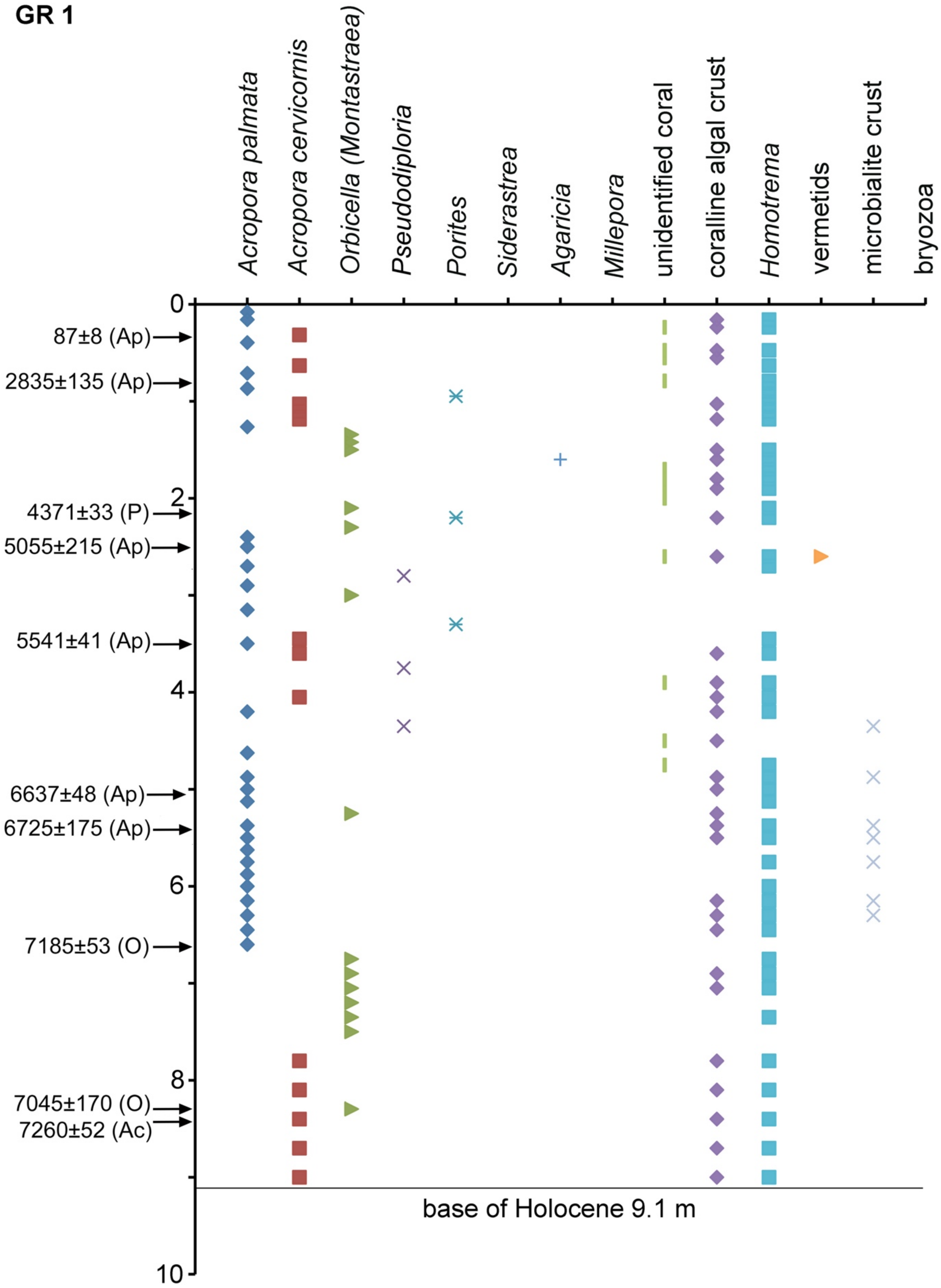

Acropora palmata
Acropora cervicornis
Orbicella (Montastraea)
Pseudodiploria
Porites
Siderastrea
Agaricia
Millepora
unidentified coral
coralline algal crust
Homotrema
vermetids
microbialite crust
bryozoa
0
2
4
6
8
10
87±8 (Ap)
2835±135 (Ap)
4371±33 (P)
5055±215 (Ap)
5541±41 (Ap)
6637±48 (Ap)
6725±175 (Ap)
7185±53 (O)
7045±170 (O)
7260±52 (Ac)
base of Holocene 9.1 m

GR 2

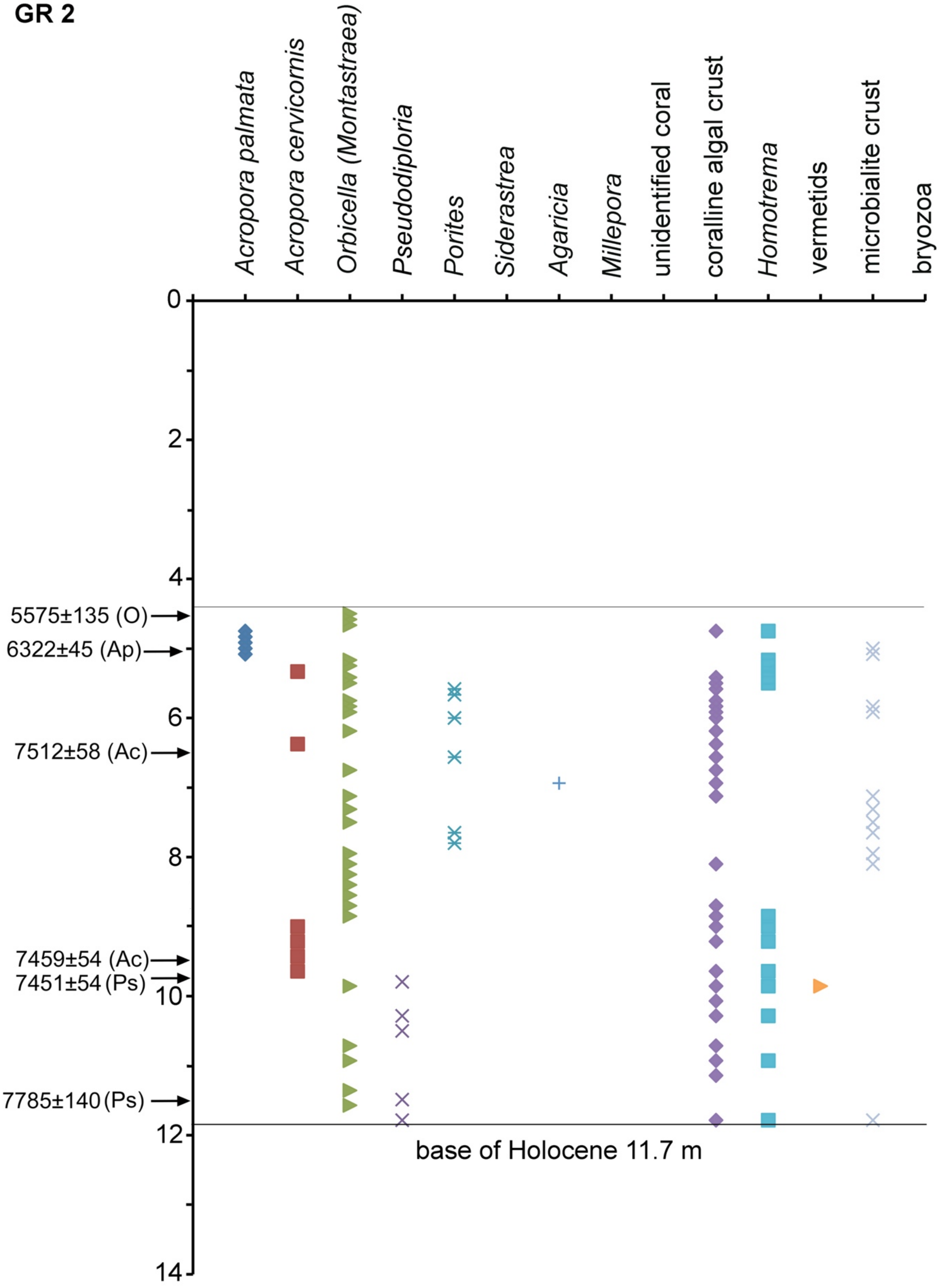

Acropora palmata
Acropora cervicornis
Orbicella (Montastraea)
Pseudodiploria
Porites
Siderastrea
Agaricia
Millepora
unidentified coral
coralline algal crust
Homotrema
vermetids
microbialite crust
bryozoa
0
2
4
6
8
10
12
14
5575±135 (O)
6322±45 (Ap)
7512±58 (Ac)
7459±54 (Ac)
7451±54 (Ps)
7785±140 (Ps)
base of Holocene 11.7 m

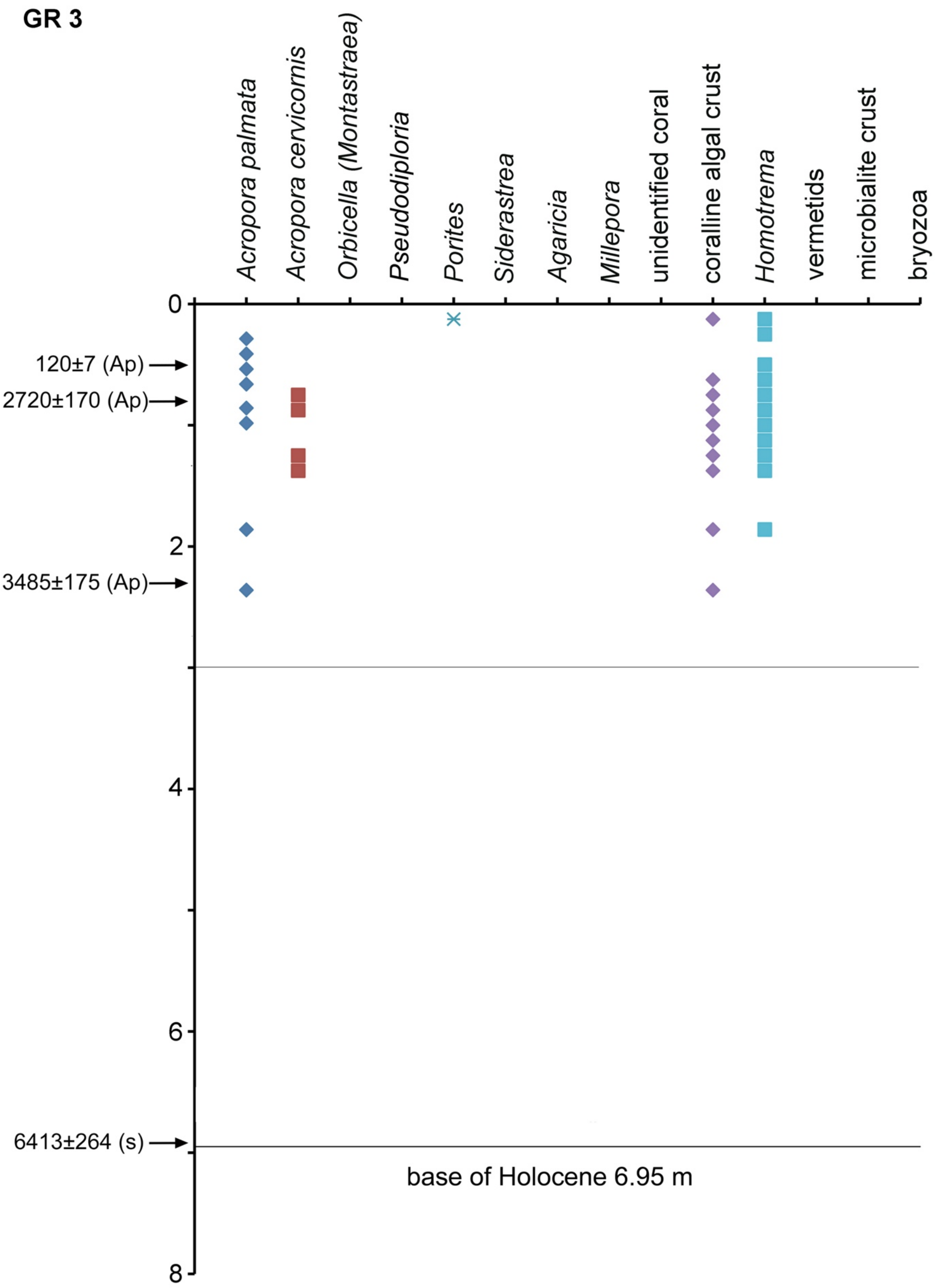
GR 3
Acropora palmata
Acropora cervicornis
Orbicella (Montastraea)
Pseudodiploria
Porites
Siderastrea
Agaricia
Millepora
unidentified coral
coralline algal crust
Homotrema
vermetids
microbialite crust
bryozoa
0
2
4
6
8
120±7 (Ap)
2720±170 (Ap)
3485±175 (Ap)
6413±264 (s)
base of Holocene 6.95 m

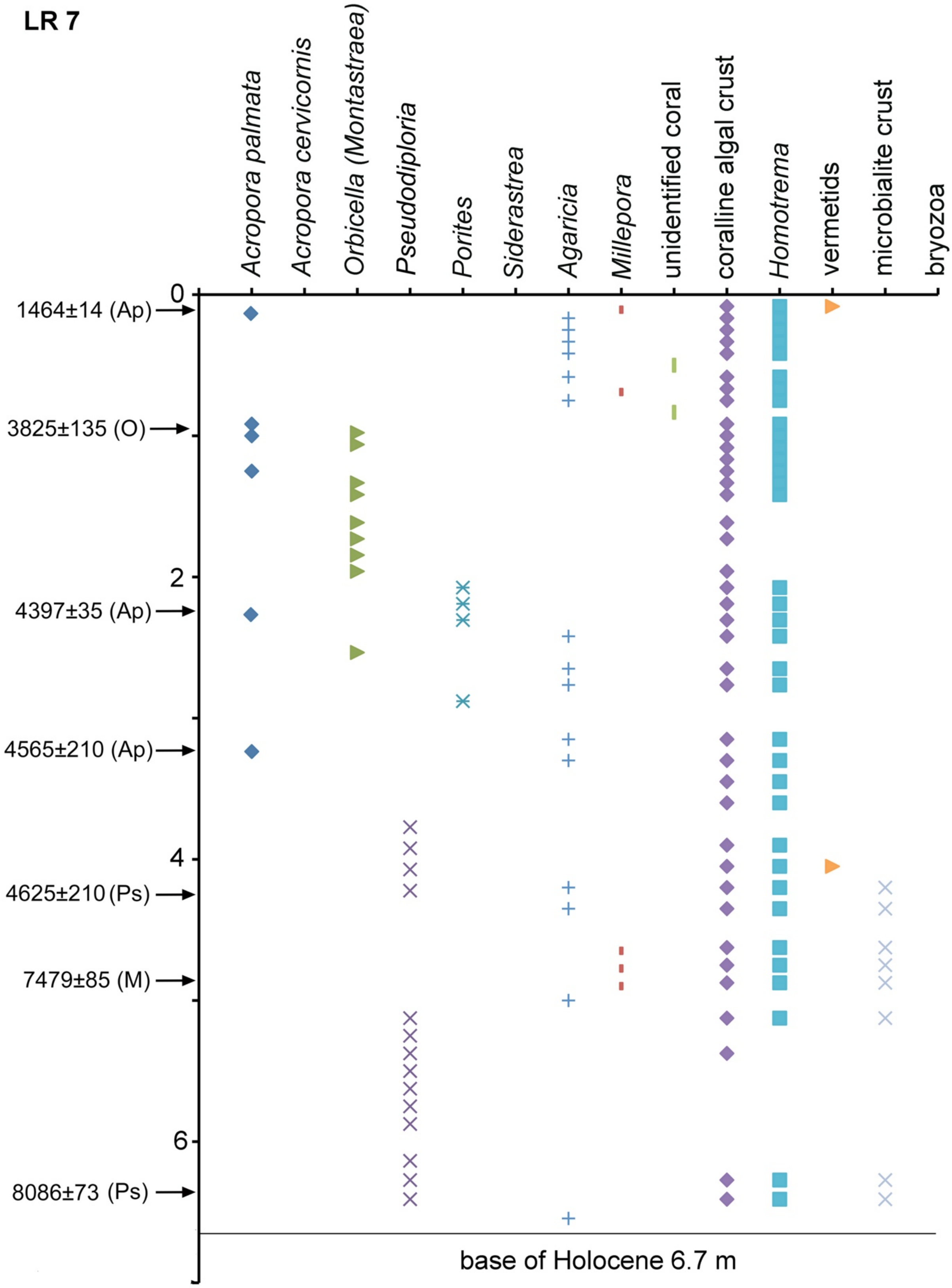
LR 7
Acropora palmata
Acropora cervicornis
Orbicella (Montastraea)
Pseudodiploria
Porites
Siderastrea
Agaricia
Millepora
unidentified coral
coralline algal crust
Homotrema
vermetids
microbialite crust
bryozoa
0
2
4
6
1464±14 (Ap)
3825±135 (O)
4397±35 (Ap)
4565±210 (Ap)
4625±210 (Ps)
7479±85 (M)
8086±73 (Ps)
base of Holocene 6.7 m

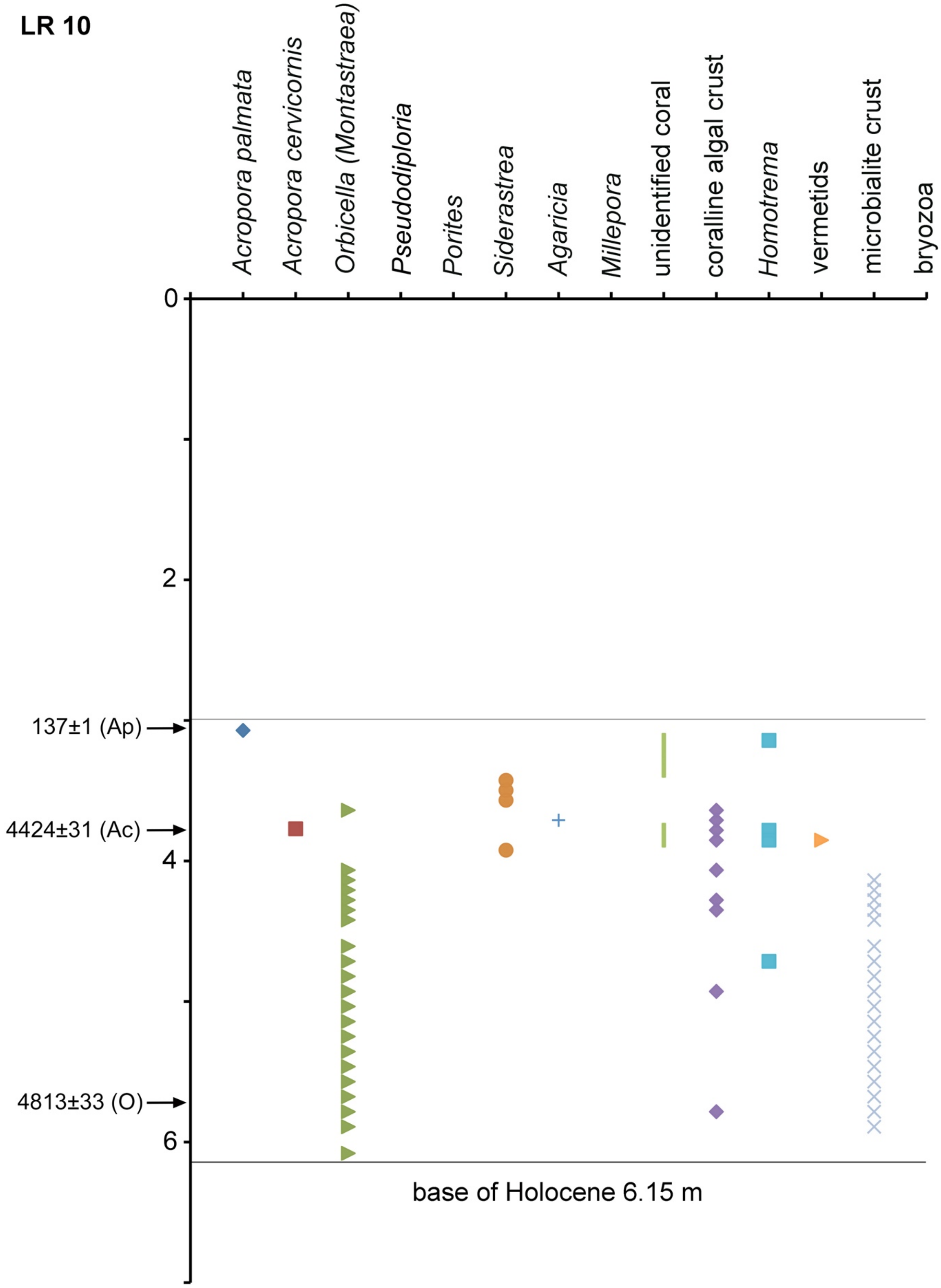

LR 10
Acropora palmata
Acropora cervicornis
Orbicella (Montastraea)
Pseudodiploria
Porites
Siderastrea
Agaricia
Millepora
unidentified coral
coralline algal crust
Homotrema
vermetids
microbialite crust
bryozoa
0
2
4
6
137±1 (Ap)
4424±31 (Ac)
4813±33 (O)
base of Holocene 6.15 m

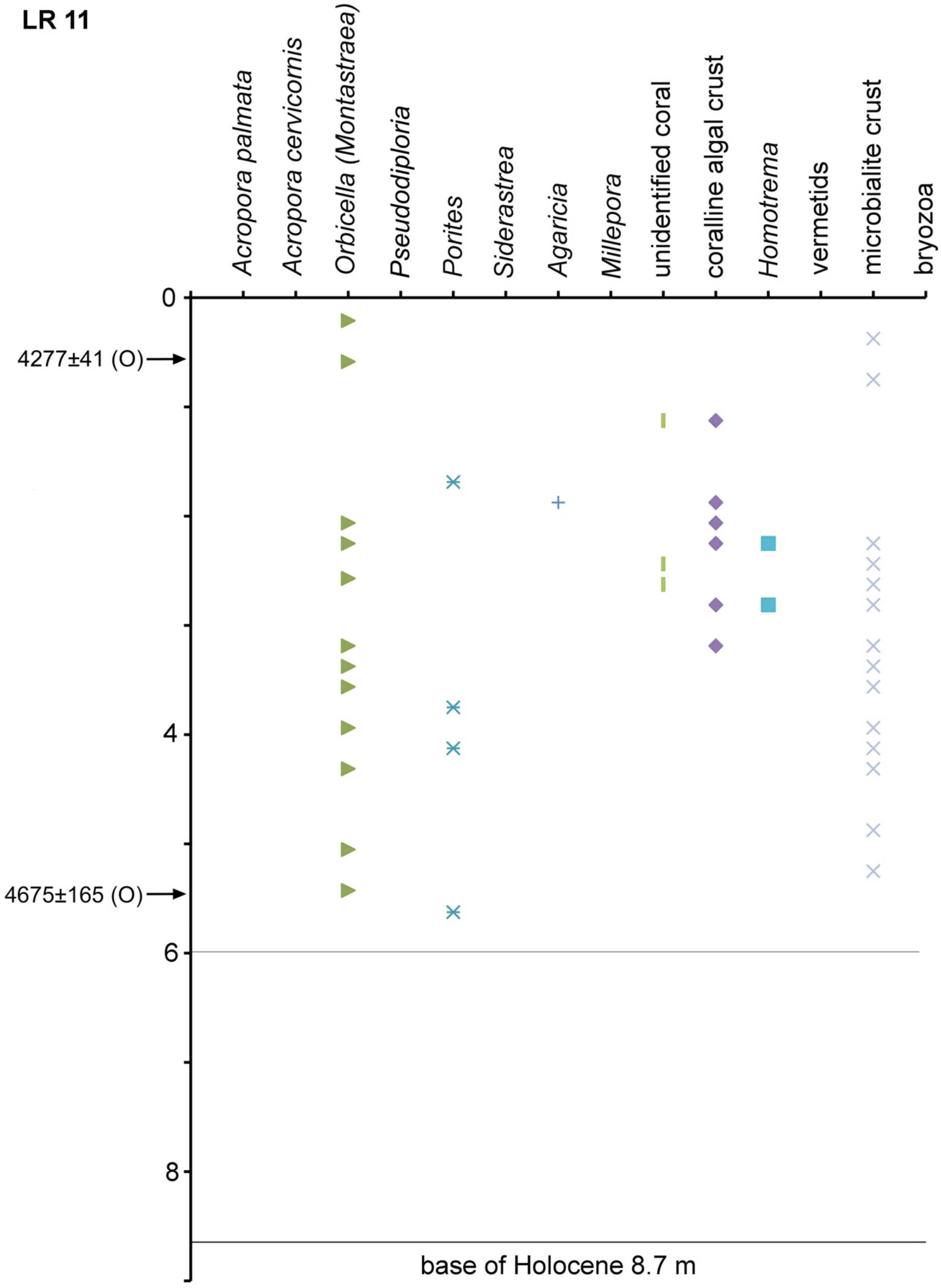
LR 11
Acropora palmata
Acropora cervicornis
Orbicella (Montastraea)
Pseudodiploria
Porites
Siderastrea
Agaricia
Millepora
unidentified coral
coralline algal crust
Homotrema
vermetids
microbialite crust
bryozoa
0
4
6
8
4277±41 (O)
4675±165 (O)
base of Holocene 8.7 m

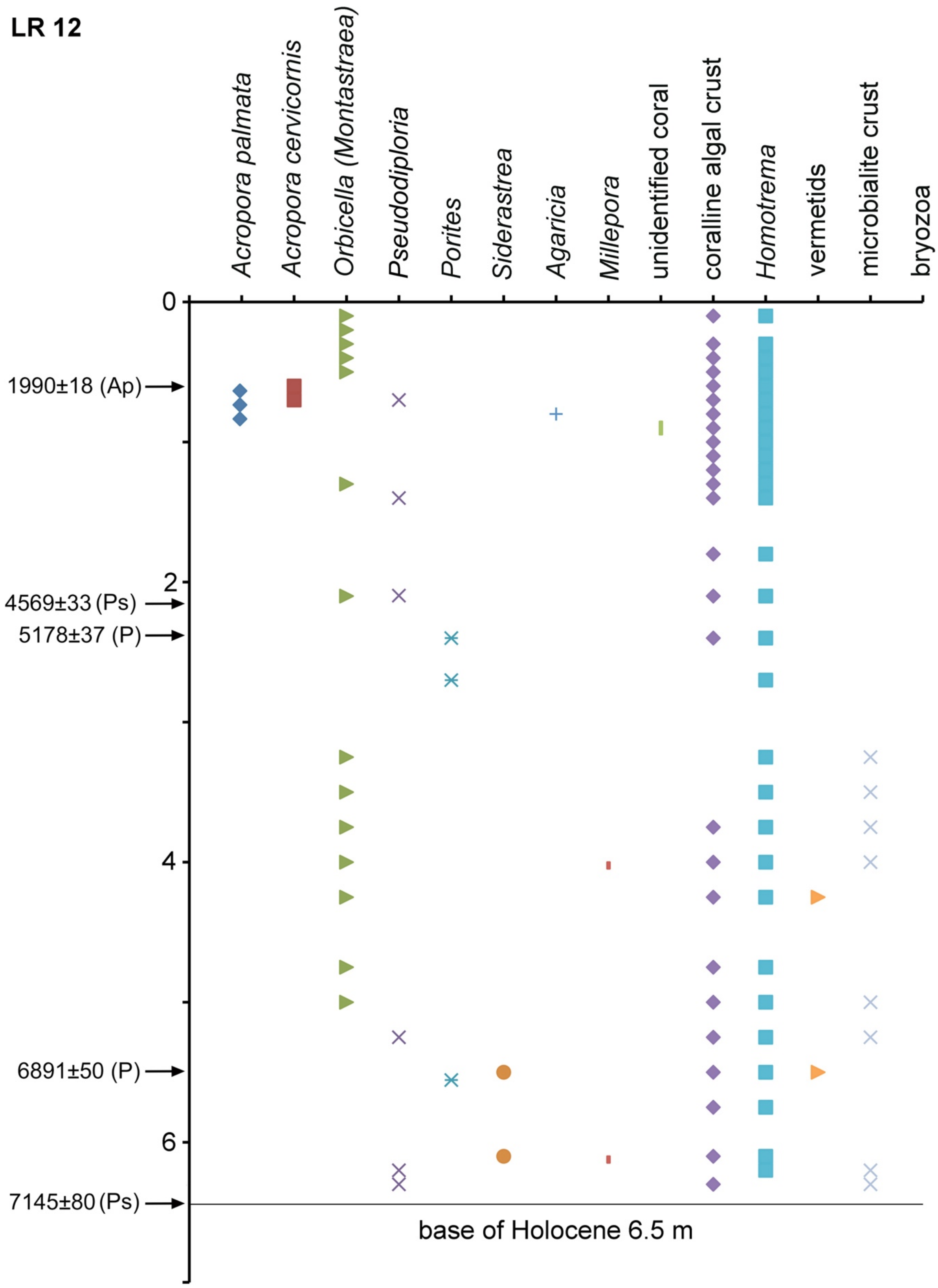
LR 12
Acropora palmata
Acropora cervicornis
Orbicella (Montastraea)
Pseudodiploria
Porites
Siderastrea
Agaricia
Millepora
unidentified coral
coralline algal crust
Homotrema
vermetids
microbialite crust
bryozoa
0
2
4
6
1990±18 (Ap)
4569±33 (Ps)
5178±37 (P)
6891±50 (P)
7145±80 (Ps)
base of Holocene 6.5 m

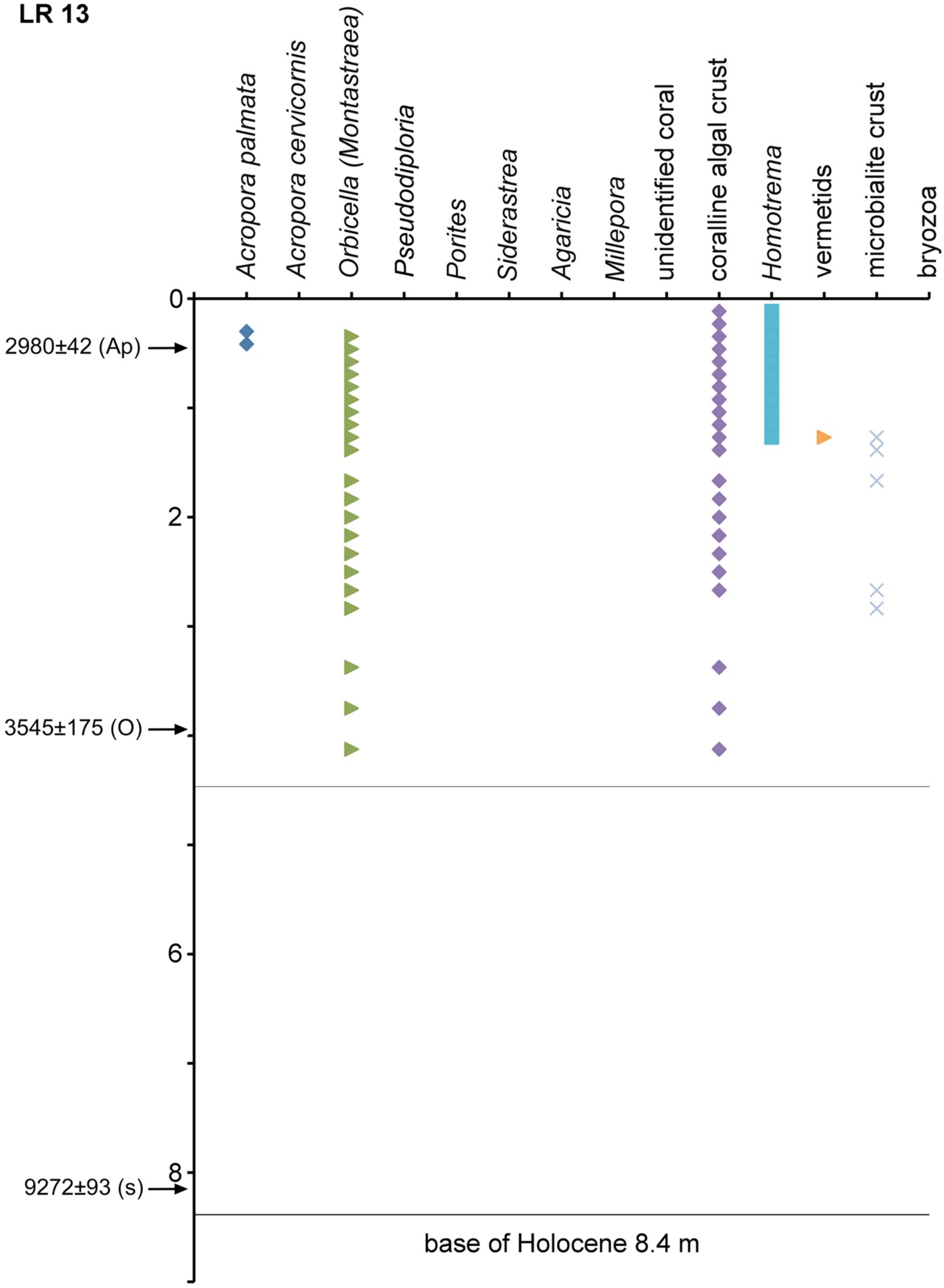
LR 13
Acropora palmata
Acropora cervicornis
Orbicella (Montastraea)
Pseudodiploria
Porites
Siderastrea
Agaricia
Millepora
unidentified coral
coralline algal crust
Homotrema
vermetids
microbialite crust
bryozoa
0
2
6
8
2980±42 (Ap)
3545±175 (O)
9272±93 (s)
base of Holocene 8.4 m

LR 14

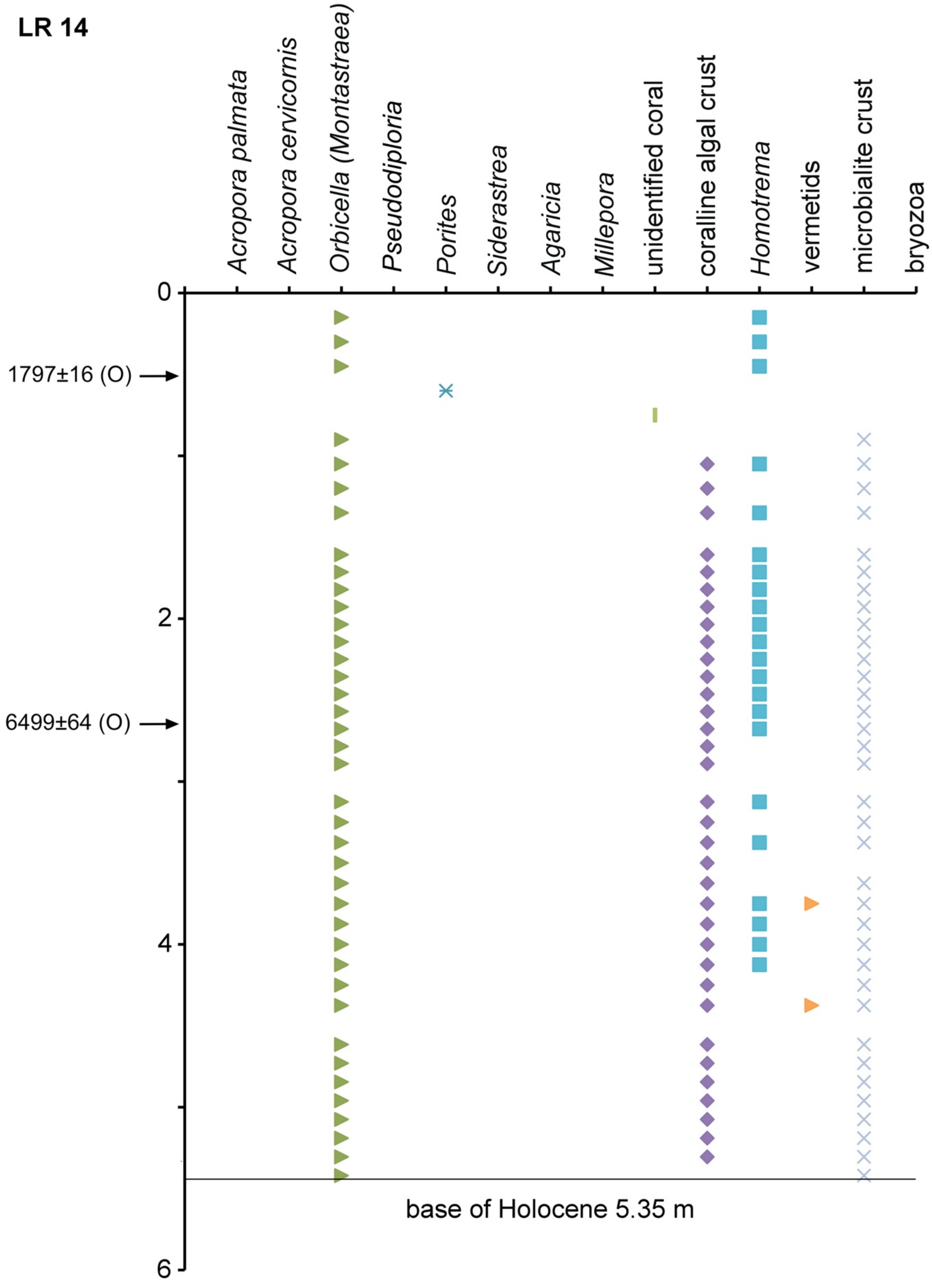
Acropora palmata
Acropora cervicornis
Orbicella (Montastraea)
Pseudodiploria
Porites
Siderastrea
Agaricia
Millepora
unidentified coral
coralline algal crust
Homotrema
vermetids
microbialite crust
bryozoa
0
2
4
6
1797±16 (O)
6499±64 (O)
base of Holocene 5.35 m

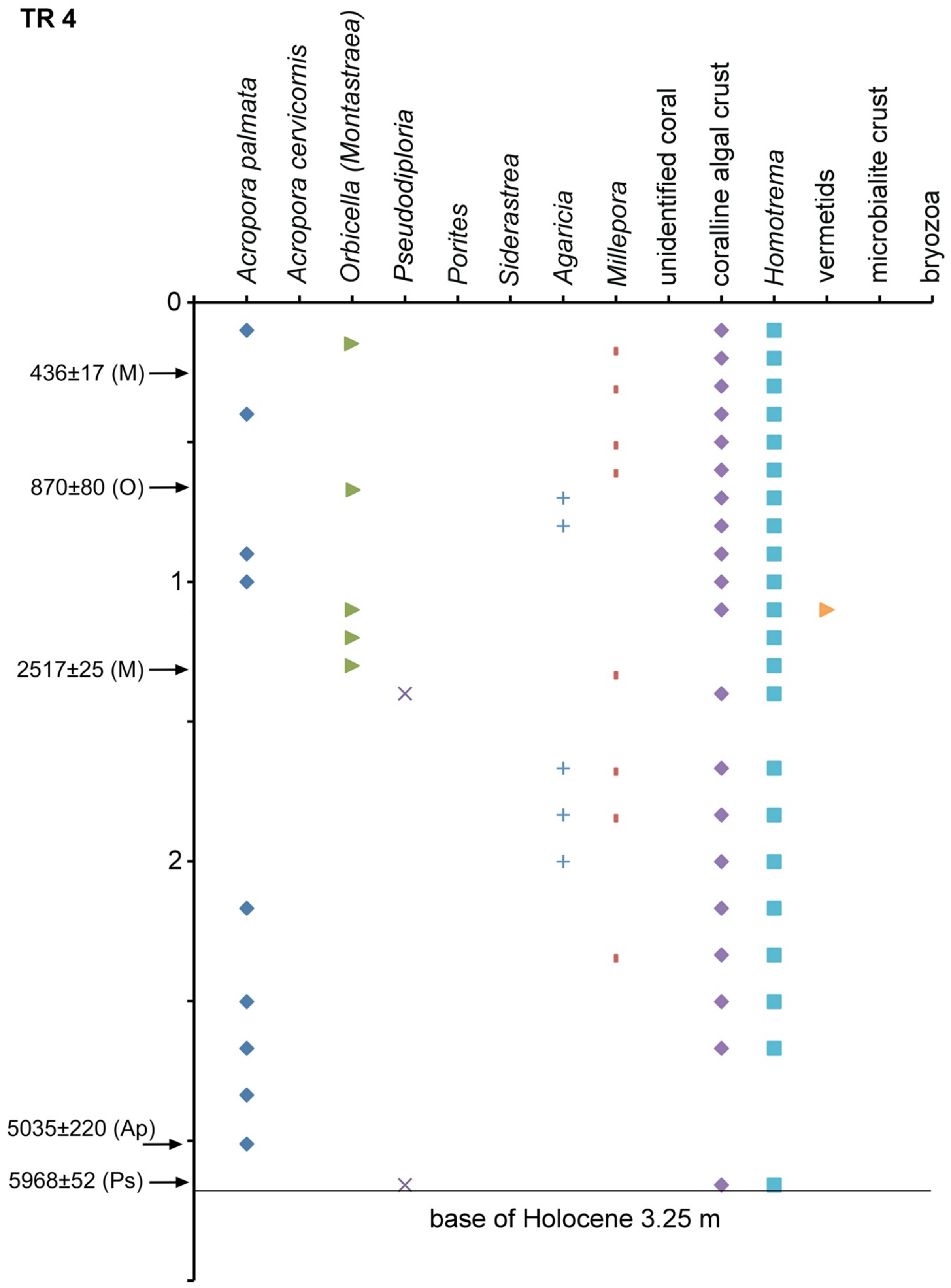
TR 4
Acropora palmata
Acropora cervicornis
Orbicella (Montastraea)
Pseudodiploria
Porites
Siderastrea
Agaricia
Millepora
unidentified coral
coralline algal crust
Homotrema
vermetids
microbialite crust
bryozoa
0
1
2
436±17 (M)
870±80 (O)
2517±25 (M)
5035±220 (Ap)
5968±52 (Ps)
base of Holocene 3.25 m

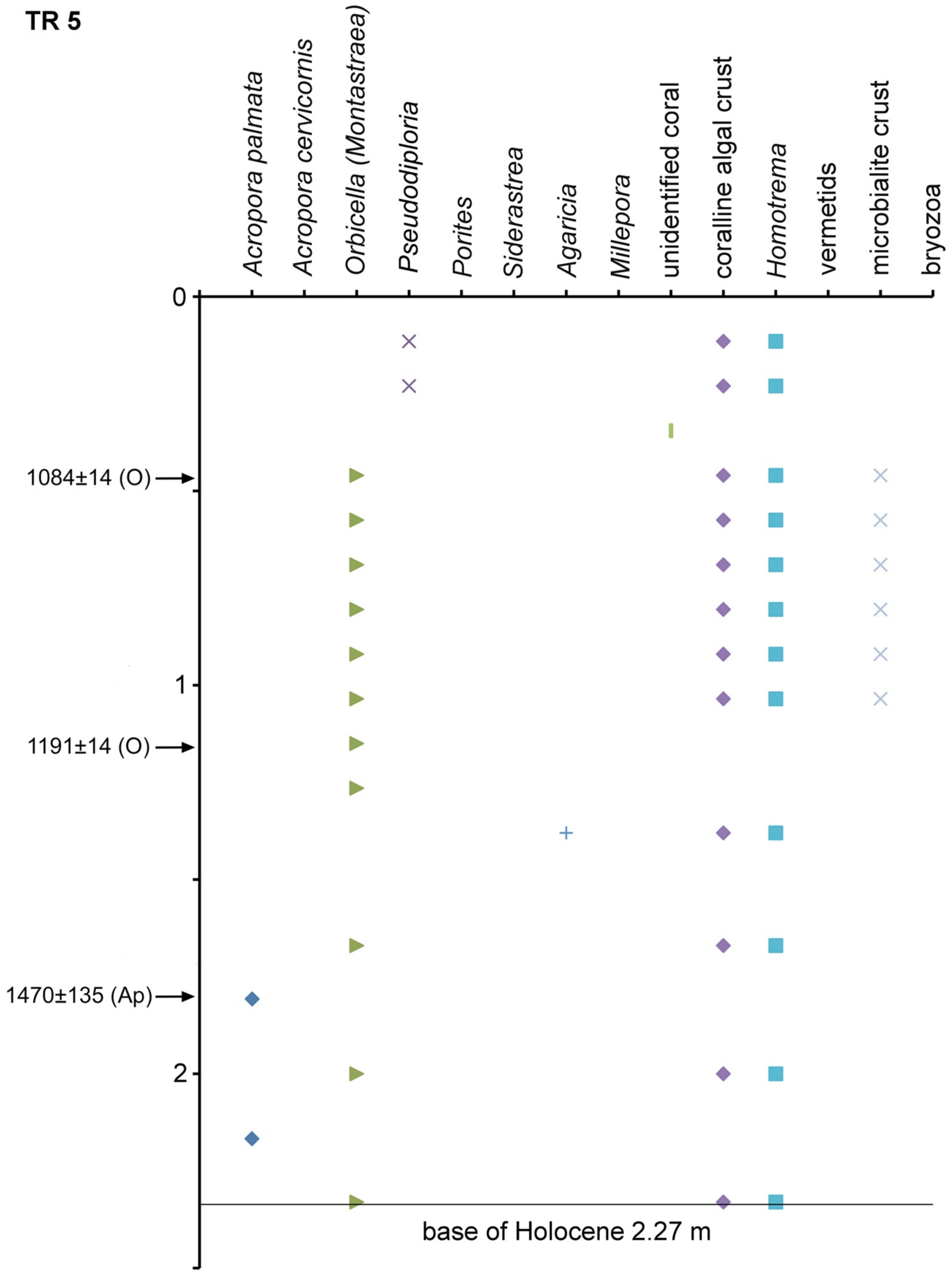
TR 5
Acropora palmata
Acropora cervicornis
Orbicella (Montastraea)
Pseudodiploria
Porites
Siderastrea
Agaricia
Millepora
unidentified coral
coralline algal crust
Homotrema
vermetids
microbialite crust
bryozoa
0
1
2
1084±14 (O)
1191±14 (O)
1470±135 (Ap)
base of Holocene 2.27 m
